# Parallel solid-phase synthesis of diaryltriazoles

**DOI:** 10.3762/bjoc.8.115

**Published:** 2012-07-06

**Authors:** Matthias Wrobel, Jeffrey Aubé, Burkhard König

**Affiliations:** 1 Fakultät für Chemie und Pharmazie, Universität Regensburg, D-93040 Regensburg, Germany, Fax: +49 9419431717; 2 Department of Medicinal Chemistry and the Chemical Methodology and Library Development Center, University of Kansas, Delbert M. Shankel Structural Biology Center, 2121 Simons Drive, West Campus, Lawrence, KS 66047, USA

**Keywords:** chemical diversity, Huisgen cycloaddition, library synthesis, peptidomimetics, solid phase synthesis, triazole

## Abstract

A series of substituted diaryltriazoles was prepared by a solid-phase-synthesis protocol using a modified Wang resin. The copper(I)- or ruthenium(II)-catalyzed 1,3-cycloaddition on the polymer bead allowed a rapid synthesis of the target compounds in a parallel fashion with in many cases good to excellent yields. Substituted diaryltriazoles resemble a molecular structure similar to established terphenyl-alpha-helix peptide mimics and have therefore the potential to act as selective inhibitors for protein–protein interactions.

## Introduction

**Scheme 1:**
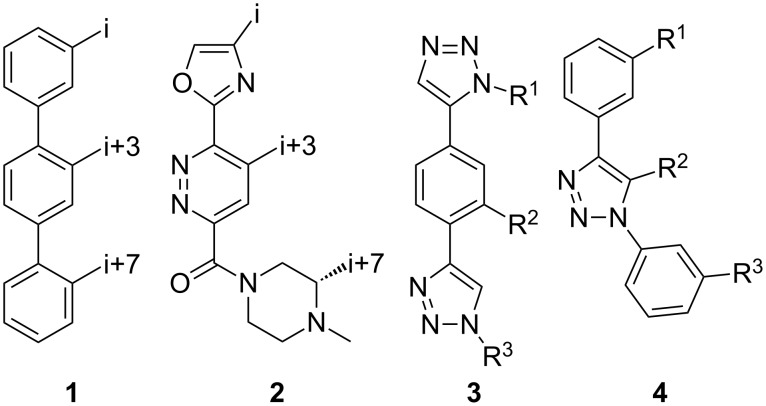
Terphenyl scaffold **1** [13–14]; oxazole-pyridazine-piperazine **2** [14–15] and aryl-triazoles **3** and **4** [15–16] as α-helix mimetics.

The α-helix was the first-described secondary structure of peptides discovered by Linus Pauling in 1951 [1]. With about 30% of the amino acids in proteins being part of α-helices [2], it is the most common secondary structure found in proteins [3]. Protein–protein as well as protein–DNA and protein–RNA interactions often involve α-helices as recognition motifs on protein surfaces [4]. These helices are important targets for new drugs, but stabilization of the helix folding for small structures with less than 15 residues still remains a challenge [5–6]. Thus, new attempts have been made to design low-molecular-weight ligands that disrupt protein–protein interactions [7]. For example, fast proteolytic degradation observed with small peptide-based compounds [8], can be overcome by compounds stabilized by non-natural amino acids [9] or cross-linked between side chains and the backbone [10]. Replacement of the complete backbone by a nonpeptidic scaffold, which positions side chains in the typical i, i+3 and i*+*7 arrangement of an α-helix is another successful strategy [11]. Horwell pioneered this type of peptidomimetics and showed that 1,6-disubstituted indanes can imitate the helix residues i and i*+*1 [12–13]. Hamilton reported a 3,2′,2″-substituted terphenyl scaffold with a spatial orientation that mimics the i, i+3 and i+7 moieties on the surface of an α-helical peptide [14]. Inspired by the terphenyl-based α-helix mimetics **1**, several related compounds containing three or more adjacent aryl rings (Scheme 1), such as **2**, were reported [15]. However, the synthesis of substituted triaryl compounds can be tedious, and the predictability of their potency and selectivity as inhibitors is still limited. We have recently reported the synthesis of triazole-based α-helix mimetics **3** and **4** [16], which are efficiently available through azide–alkyne cycloadditions [17]. We now report the use of this chemistry to prepare libraries of potential inhibitors of protein–protein interactions.

## Results and Discussion

### Synthesis of azide-functionalized Wang resins

**Scheme 2:**
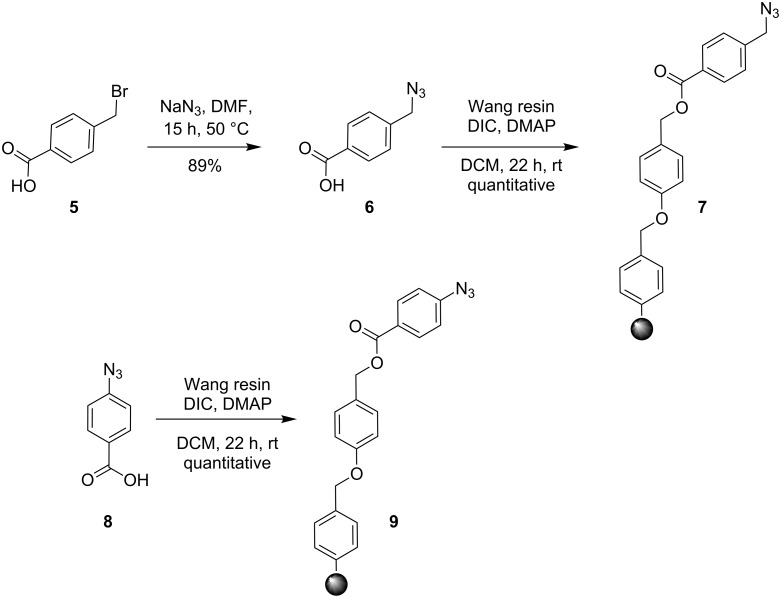
Synthesis of azido-functionalized resins **7** and **9**.

Two azide-functionalized resins were prepared for the solid-phase synthesis of diaryl-triazoles. The commercially available 4-(bromomethyl)benzoic acid (**5**) was converted into azide **6** in anhydrous DMF with sodium azide under heating. Coupling to Wang resin in dichloromethane, by using DIC and DMAP as coupling reagents, gave resin **7** in quantitative yield (Scheme 2) [18]. Commercially available 4-azidobenzoic acid (**8**) gave resin **9** in an analogous esterification of a Wang resin.

### Solid-phase synthesis of diaryltriazoles

The conditions for the solid-phase synthesis of diaryltriazoles on functionalized Wang resin **7** were optimized by using five different alkynes **10a**–**e**, containing acyclic or cyclic aliphatic moieties, simple arenes and 1-(but-3-yn-2-yl)-3-(4-chlorophenyl)-1-methylurea (**10c**) as an example of a more complex alkyne. The azide–alkyne [3 + 2] cycloaddition was catalyzed with copper(II) sulfate pentahydrate and L-ascorbic acid in DMF overnight at room temperature. A solution of EDTA was added to remove the remaining copper cations from the resin. Resin cleavage under acidic conditions with TFA in DCM gave compounds **11a**–**e** in moderate to excellent overall yields of 57 to 90% (Table 1). Due to the solid phase synthesis protocol the crude material purity was typically high, ranging from 70 to 90%. Alkyne **10d** and **10e** bearing hydroxy groups were converted quantitatively, but elimination of water occurred in the presence of TFA. The dehydrated products were obtained in 57 and 71%. The remaining material was the corresponding hydroxylated product.

**Table 1:** Copper-catalyzed [2 + 3] cycloadditions of resin-bound azide **7** with five terminal alkynes.

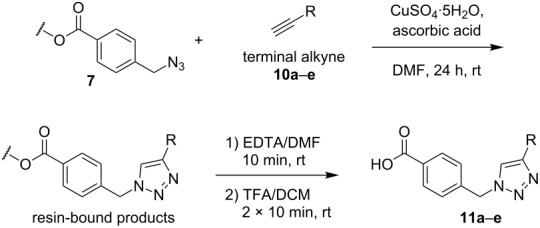		

terminal alkyne **10**	product **11**	yield [%]

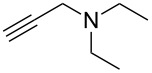 **10a**	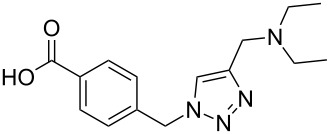 **11a**	63
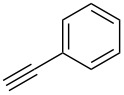 **10b**	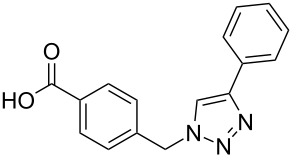 **11b**	90
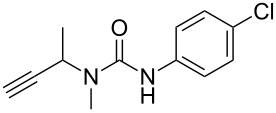 **10c**	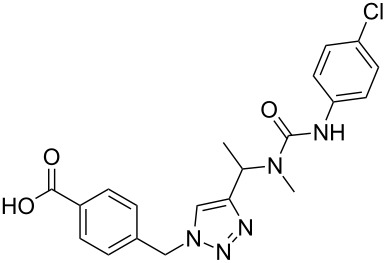 **11c**	81
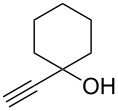 **10d**	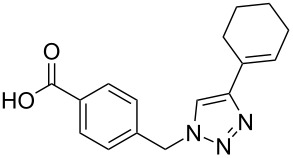 **11d**	57
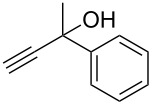 **10e**	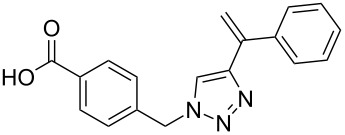 **11e**	71

### Parallel synthesis of a compound library

A larger compound library was prepared by using resins **7** and **9**, 15 different terminal alkynes **10f–t** and either copper or ruthenium-catalyzed [2 + 3] cycloadditions. The three reactions and the obtained products **11** (reaction 1), **12** (reaction 2) and **13** (reaction 3) are summarized in Table 2.

**Table 2:** Copper-catalyzed [2 + 3] cycloadditions of resin bound azide **7** with five terminal alkynes. Compounds **13**, with the exception of **13f**, were only characterized during compound library synthesis, by HPLC–MS analysis.

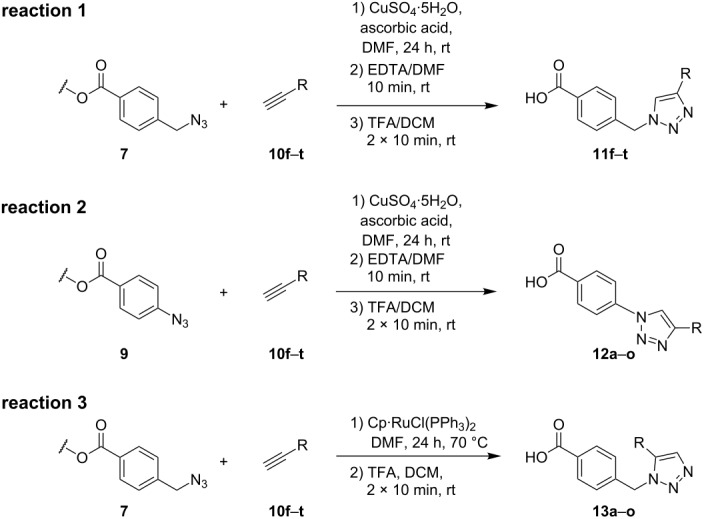			

alkyne **10**	product after cleavage from resin (yield)		

	**reaction 1** resin **7**, catalyst CuSO_4_	**reaction 2** resin **9**, catalyst CuSO_4_	**reaction 3** resin **7**, catalyst Cp·RuCl(PPh_3_)_2_

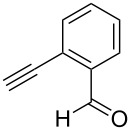 **10f**	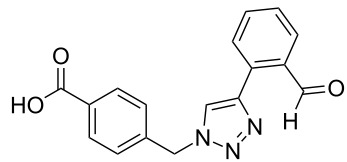 **11f** (95%)	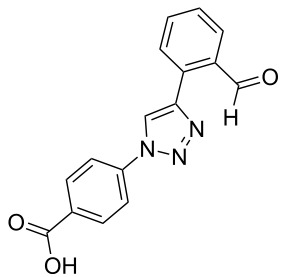 **12a** (49%)	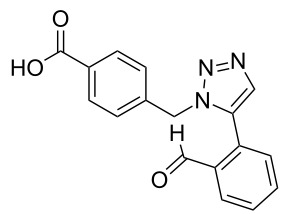 **13a** not obtained

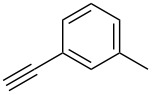 **10g**	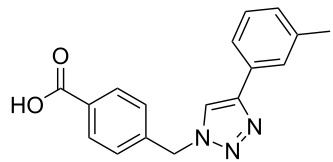 **11g** (99%)	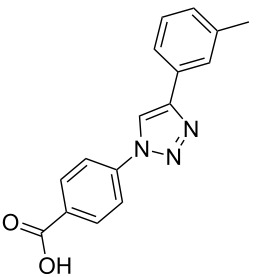 **12b** (44%)	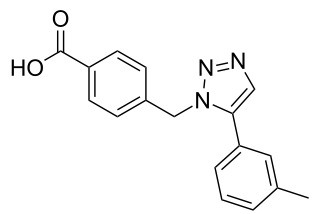 **13b** (43%)

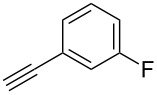 **10h**	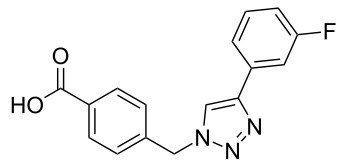 **11h** (97%)	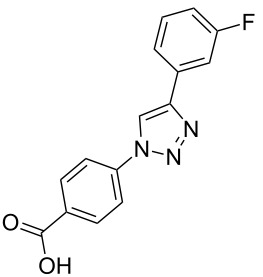 **12c** (45%)	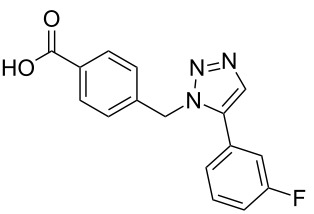 **13c** (89%)

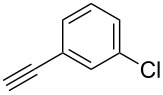 **10i**	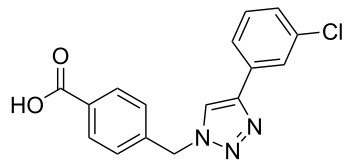 **11i** (98%)	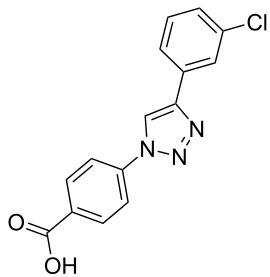 **12d** (44%)	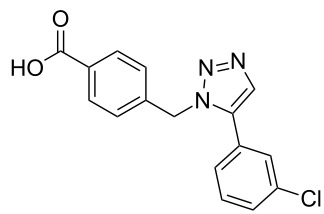 **13d** (89%)

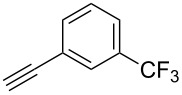 **10j**	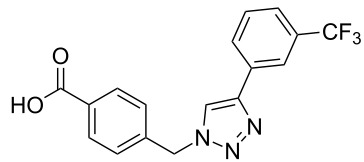 **11j** (98%)	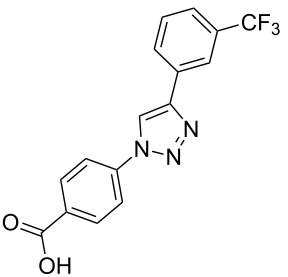 **12e** (41%)	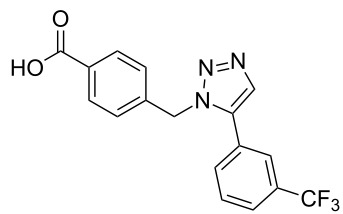 **13e** (90%)

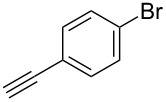 **10k**	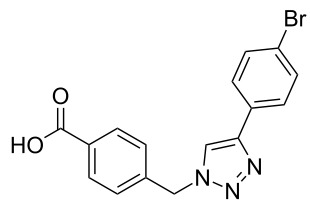 **11k** (97%)	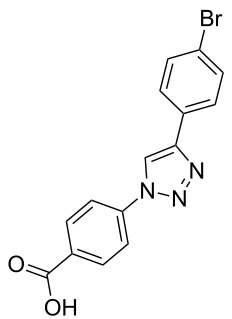 **12f** (21%)	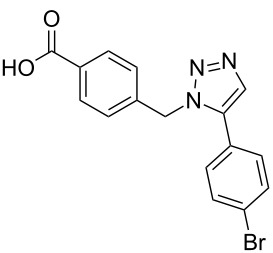 **13f** (48%)

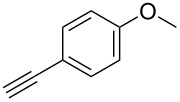 **10l**	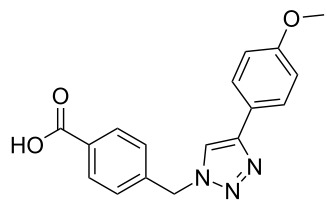 **11l** (98%)	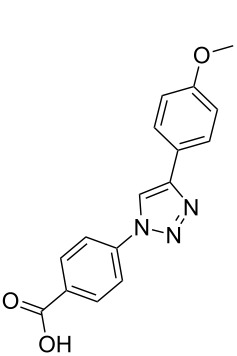 **12g** (52%)	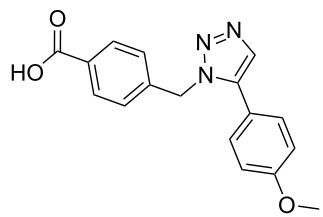 **13g** (94%)

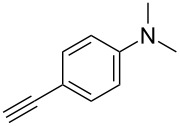 **10m**	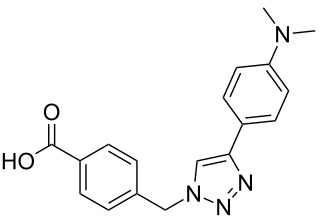 **11m** (88%)	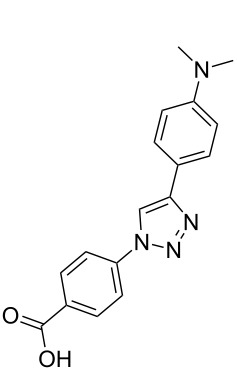 **12h** (38%)	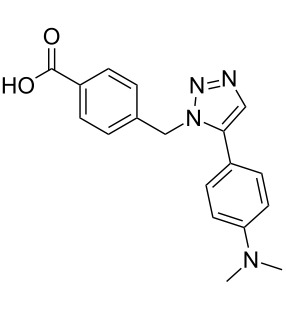 **13h** (96%)

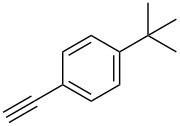 **10n**	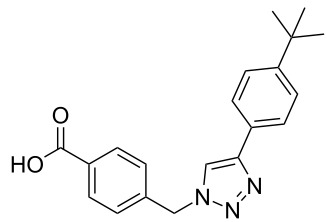 **11n** (98%)	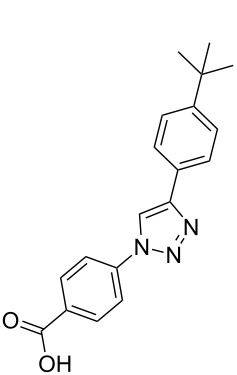 **12i** (41%)	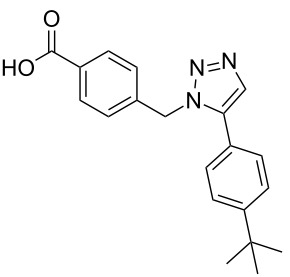 **13i** (80%)

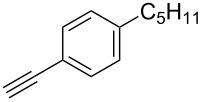 **10o**	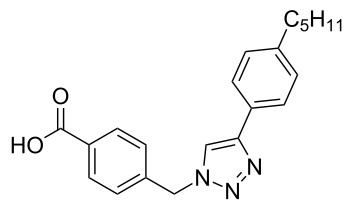 **11o** (97%)	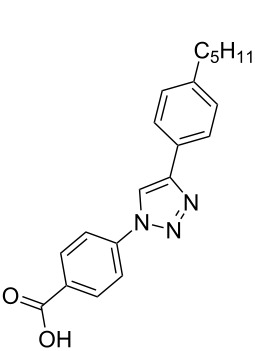 **12j** (63%)	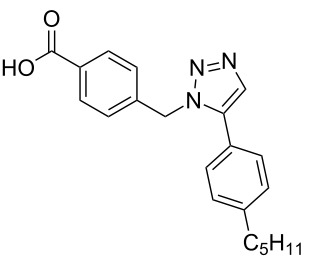 **13j** (82%)

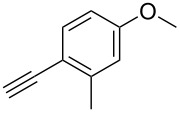 **10p**	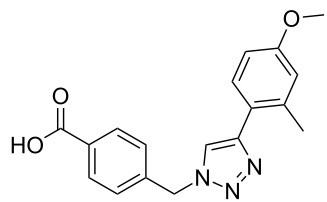 **11p** (99%)	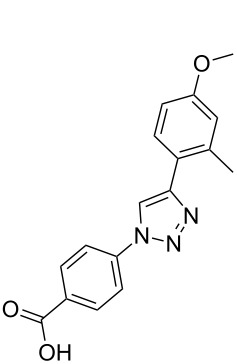 **12k** (47%)	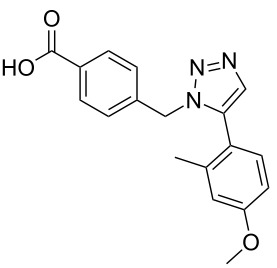 **13k** (89%)

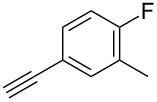 **10q**	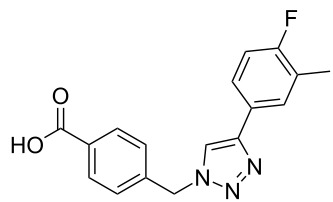 **11q** (98%)	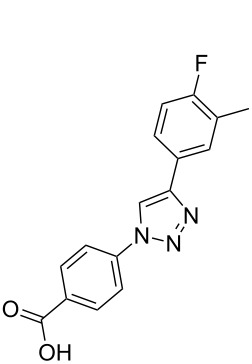 **12l** (40%)	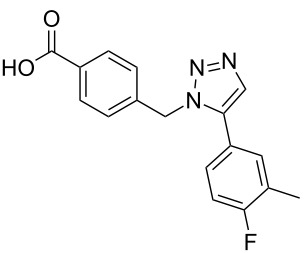 **13l** (89%)

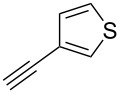 **10r**	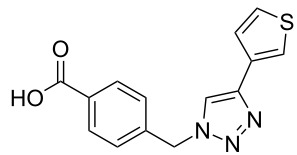 **11r** (98%)	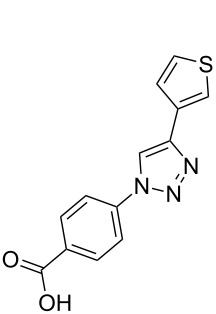 **12m** (61%)	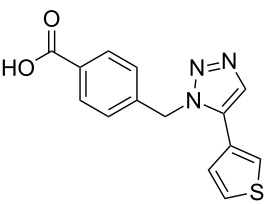 **13m** (92%)

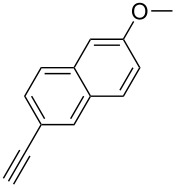 **10s**	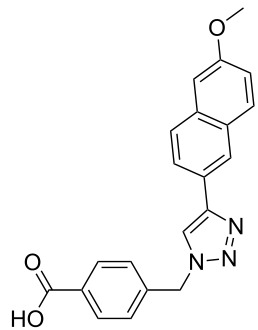 **11s** (94%)	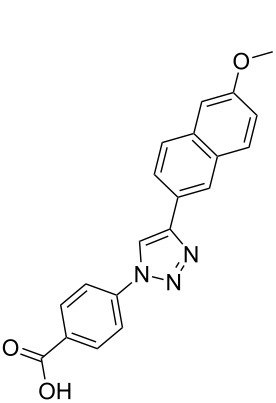 **12n** (26%)	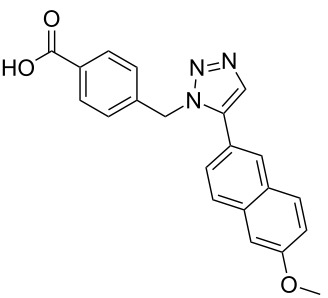 **13n** (95%)

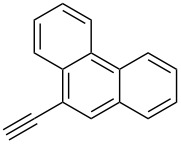 **10t**	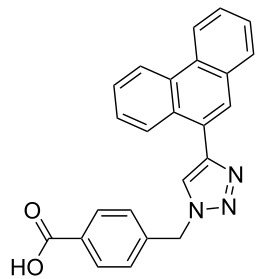 **11t** (96%)	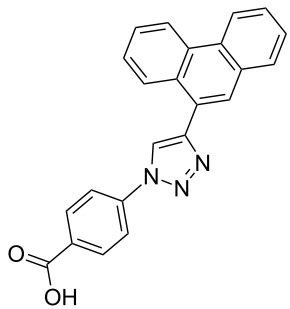 **12o** not obtained	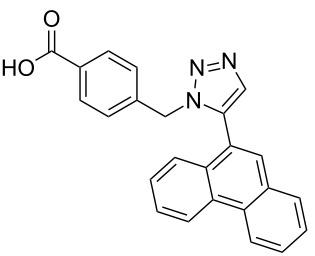 **13o** (74%)

Reaction 1 follows the established protocol and, gave after removal of the copper salts with a solution of EDTA and TFA cleavage, the corresponding products in good to quantitative yields (88–99%). Resin **9** was used in reaction 2 under otherwise identical reaction conditions. The use of an aromatic azide leads to more rigid products containing three adjacent aromatic rings: The central triazole and the phenyl ring of the benzoic acid as constant structural elements and the third ring consisting either of substituted benzenes, heteroarenes or a polycyclic aromatic compound. Lower product yields were obtained in this series of compounds, ranging from 21 to 63%. The lower reactivity of the aromatic azide and the increased steric demand may explain the decrease in yield in comparison to that of the former series of compounds. The formation of the compound **12o**, bearing a particularly bulky substituent, was not observed. Replacing the copper(I) catalyst by a ruthenium(II) complex allows the preparation of regioisomers in reaction 3. Instead of the 1,4-disubstituted triazoles obtained from copper(I) catalysis, the complex pentamethylcyclopentadienylbis(triphenylphosphine)ruthenium(II) chloride leads to 1,5-disubstituted triazole compounds [19]. 4-(Azidomethyl)benzoic acid functionalized Wang resin **7** and the alkynes **10f**–**t** were reacted in DMF at 70 °C overnight, and the products **13b**–**o** were obtained after TFA cleavage from the resin in moderate to good yields ranging from 43–96%. Only compound **13a** could not be obtained. The proton NMR analysis allows us to clearly distinguish between 1,4- and 1,5-disubstituted triazoles due to a characteristic shift of the triazole proton resonance. The triazole proton of the 1,4-disubstituted ring in compound **11k** shows a ^1^H NMR resonance at δ = 8.71 (400 MHz, DMSO-*d*_6_), while the resonance signal for the triazole proton of product **13f** is observed at δ = 8.00 (400 MHz, DMSO-*d*_6_). Compounds **13**, with the exception of compound **13f**, were only characterized by mass spectrometry during the synthesis of the compound library.

The copper-mediated [2 + 3] cycloadditions are restricted to terminal alkynes. However, the ruthenium-catalysis allows the use of internal alkynes. In preliminary work, resin **7** was therefore reacted with internal alkynes **14a**–**c** and pentamethylcyclopentadienylbis(triphenylphosphine)ruthenium(II) chloride as catalyst in DMF at 70 °C overnight followed by TFA cleavage [20]. LC–MS analysis of the crude product revealed the formation of compounds **15a**–**c** in high yields of 78–98% (Table 3).

**Table 3:** Ruthenium-catalyzed [2 + 3] cycloadditions of resin-bound azide **7** with three disubstituted alkynes.

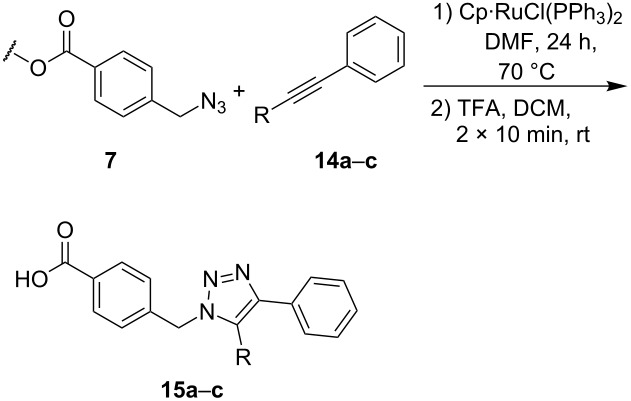	

alkyne	product **15** (yield)^a^

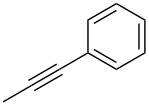 **14a**	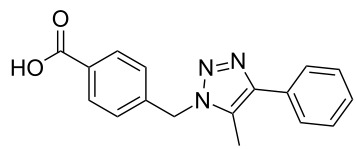 **15a** (78%)
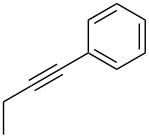 **14b**	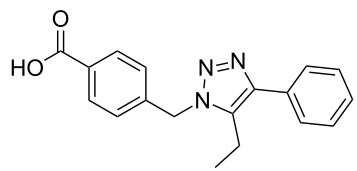 **15b** (84%)
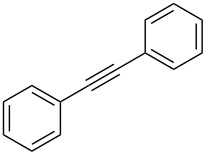 **14c**	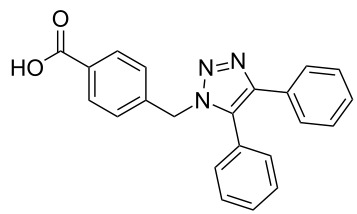 **15c** (98%)

^a^Yields based on LC–MS; compound **12b** was used as standard.

## Conclusion

Diaryltriazoles were obtained in an efficient three-step solid-phase procedure. Immobilization of aromatic azides on commercial Wang resin followed by copper(I)- or ruthenium(II)-catalyzed 1,3-cycloaddition and subsequent cleavage of the product from the resin gave the target structures in good to excellent yields with the possibility to introduce a wide variety of different substituents. The alternative use of copper or ruthenium catalysis for the on-bead cycloaddition gives regioisomeric products, which extends the diversity of the compound collection. The method may find application in the combinatorial search for selective protein–protein inhibitors. To that end, most of the compounds prepared herein were submitted to the Molecular Libraries Small Molecular Repository for ongoing inclusion in high-throughput screening activities.

## Experimental

### General procedures

**GP 1 – Coupling of benzoic acid derivatives 6 and 8 on Wang resin:** Wang resin (1 equiv) was preswollen in dichloromethane (0.8 mL/100 mg resin) for 2 h at room temperature. Subsequently, both coupling reagents *N*,*N′*-diisopropylcarbodiimide (3.5 equiv) and dimethylaminopyridine (0.5 equiv) were added. After the addition of the benzoic acid derivative **6** or **8** (2.5 equiv) the reaction mixture was stirred for 20 h at room temperature. The resin was first washed with dimethylformamide, methanol and dichloromethane (each solvent 3 × 0.8 mL/100 mg resin), and then dried in high vacuum for 3 h.

**GP 2 – Huisgen 1,3-dipolar cycloaddition of solid-phase-immobilized azides with terminal alkynes by copper(I) catalysis:** An azide-functionalized Wang resin **7** or **9** (1 equiv) was preswollen in dimethylformamide (1.5 mL/100 mg resin) for 2 h at room temperature. The copper(I) catalyst was prepared in situ by using L-ascorbic acid (0.5 equiv) as reducing agent and copper(II) sulfate pentahydrate (10 mol %). After the terminal alkyne (4 equiv) was added, the reaction mixture was stirred for 22 h at room temperature. The resin was washed with dimethylformamide, methanol and dichloromethane (each solvent 2 mL/100 mg resin). The remaining copper cations were complexed and removed by using a solution of ethylenediaminetetraacetic acid disodium salt. For this purpose, a 1:1 mixture of dimethylformamide and disodium EDTA (aq., sat.) was added to the resin and stirred for 10 min at room temperature. Again washing steps with water, dimethylformamide, methanol and dichloromethane (each solvent 3 × 2 mL/100 mg resin) were carried out.

**GP 3 – Huisgen 1,3-dipolar cycloaddition of solid-phase-immobilized azides with terminal or internal alkynes by ruthenium(II) catalysis:** The azide functionalized Wang resin (1 equiv) was preswollen in dimethylformamide (2 mL/100 mg resin) for 2 h at room temperature. Subsequently, the catalyst complex pentamethylcyclopentadienylbis(triphenylphosphine)ruthenium(II) chloride, Cp·RuCl(PPh_3_)_2_, (5 mol %) and either a terminal or an internal alkyne (4 equiv) were added. After the reaction mixture was stirred for 22 h at 70 °C, the resin was washed with dimethylformamide, methanol and dichloromethane (each solvent 3 × 2 mL/100 mg resin).

**GP 4 – Cleavage of solid-phase resin-bound molecules with TFA:** The swollen resin was treated with a 1:4 mixture of trifluoroacetic acid and dichloromethane (1 mL/100 mg resin). After being stirred for 10 min at room temperature, the cleaved product was rinsed out of the resin using dichloromethane (1.5 mL/100 mg resin). The resin was treated once more with the 20% trifluoroacetic acid solution (1 mL/100 mg resin), stirred for 10 min at room temperature and washed with dichloromethane (3 × 1 mL/100 mg resin). The solvent was evaporated and the product was dried in high vacuum for 4 h.

**4-(Azidomethyl)benzoic acid (6)** [21]**:** The synthetic procedure leading to this literature-known compound was improved. 4-(Bromomethyl)benzoic acid (**5**, 1.2 g, 5.58 mmol, 1.0 equiv) and sodium azide (907 mg, 13.95 mmol, 2.5 equiv) were suspended in 25 mL of anhydrous dimethylformamide under a nitrogen atmosphere. After the reaction mixture was stirred for 15 h at 50 °C the solvent was evaporated. The colorless residue was dissolved in 90 mL of water and the solution was treated with 17 mL of hydrochloric acid (*c* 1 mol/L). The precipitate was separated with a Büchner funnel, dissolved in dichloromethane and dried over potassium sulfate. After filtration, and evaporation of the solvent, 4-(azidomethyl)benzoic acid (**6**, 880 mg, 4.97 mmol, 89%) was yielded as a colorless solid and dried in high vacuum overnight; mp 135.6–136.6 °C; ^1^H NMR (300 MHz, CDCl_3_) δ 4.46 (s, 2 H, H-6), 7.44 (d, ^3^*J*_HH_ = 8.4 Hz, 2H, H-4), 8.14 (d, ^3^*J*_HH_ = 8.3 Hz, 2H, H-3); ^13^C NMR (75 MHz, CDCl_3_) δ 54.3 (−, 1C, C-6), 128.0 (+, 2C, C-4), 129.1 (C_q_, 1C, C-2), 130.8 (+, 2C, C-3), 141.5 (C_q_, 1 C, C-5), 171.4 (C_q_, 1C, C-1); IR (cm^−1^) 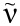: 2933 (w), 2880 (w), 2817 (w), 2656 (w), 2110 (m), 2086 (m), 1950 (w), 1682 (s), 1293 (s), 1239 (s), 707 (s), 545 (s); EIMS *m*/*z*: 177.0 (40) [M]^+^, 148.0 (80) [M − N_2_]^+^, 135.0 (100) [M − N_3_]^+^; Anal. calcd for C_8_H_7_N_3_O_2_: C, 54.24; H, 3.98; N, 23.72; found: C, 54.28; H, 4.25; N, 23.75.

## Supporting Information

File 1 Experimental details and spectra.
