# Supplementary material for: Parallel solid-phase synthesis of diaryltriazoles
Source: Beilstein J Org Chem. 2012 Jul 6;8:1027–36. doi: 10.3762/bjoc.8.115 (PMC3458720; doi:10.3762/bjoc.8.115)
Supplement: File 1 — Experimental details and spectra. [file Beilstein_J_Org_Chem-08-1027-s001.pdf]

Supporting Information

for

Parallel solid-phase synthesis of diaryltriazoles

Matthias Wrobel,[1] Jeffrey Aubé,[2] and Burkhard König*,[1]

Address: [1]Fakultät für Chemie und Pharmazie, Universität Regensburg, D-93040 Regensburg, Germany, Fax: +49 9419431717 and [2]Department of Medicinal Chemistry and the Chemical Methodology and Library Development Center, University of Kansas, Delbert M. Shankel Structural Biology Center, 2121 Simons Drive, West Campus, Lawrence, KS 66047, USA

Email: Burkhard König* - burkhard.koenig@chemie.uni-regensburg.de

* Corresponding author

Experimental details and spectra

Table of contents

Synthesis of new compounds	S2
General information and instruments	S2
Synthesis of functionalized Wang resins	S3
Synthesis of substituted triazoles	S4
Copies of spectra of new compounds	S27
Data on purity of crude material after resin cleavage	S40
References	S44

Synthesis of new compounds

General information and instruments

Nuclear magnetic resonance spectroscopy (NMR): Bruker Avance 300 (^{1}H: 300.1 MHz, ^{13}C: 75.5 MHz); Bruker Avance 400 (^{1}H: 400.1 MHz, ^{13}C: 100.6 MHz); Bruker Avance 600 Kryo (^{1}H: 600.3 MHz, ^{13}C: 150.9 MHz). The measurements were performed at 300 Kelvin [K] if not stated otherwise. The chemical shifts are in δ-values (ppm) relative to the internal or external standard TMS. The spectra were analyzed by first order and coupling constants *J* are given in hertz [Hz]. Abbreviations for the characterization of the signals: s = singlet, d = doublet, t = triplet, q = quartet, m = multiplet, bs = broad singlet, dd = doublet of doublets, dt = doublet of triplets, td = triplet of doublets, tt = triplet of triplets, ddd = doublet of doublets of doublets, dddd = doublet of doublets of doublets of doublets. Integration is determined as the relative number of protons. Error of reported values: chemical shift 0.01 ppm for ^{1}H NMR, 0.1 ppm for ^{13}C NMR; coupling constant: 0.1 Hz. The used solvent for each spectrum is reported. Infrared spectroscopy (IR): Bio-Rad Excalibur FT-IR-Spectrometer “FTS 3000 MX”; Perkin Elmer Precisely FTIR-Spectrometer “Spectrum 100”; abbreviations of the signals: s = strong, m = medium, w = weak; bs, bm, bw are broad signals with the corresponding intensity of the signal. Mass spectrometry (MS): Varian CH-5 (EI); Finnigan MAT 95 (EI); Finnigan MAT TSQ 7000 (ESI); Waters LCT Premier Micromass (ESI). Melting points (mp): Stanford Research System OptiMelt melting point apparatus; Thomas Hoover capillary melting point apparatus; all values are given in °C and are uncorrected. Elementary analysis: Microanalytical Laboratory of the University of Regensburg. Thin layer chromatography: Analytical thin layer chromatography (TLC) was performed on silica gel coated alumina plates (Merck TLC Aluminum sheets Si 60 F_{254}, layer thickness 175–225 µm). Visualization was done by UV-light (λ = 254 and 366 nm). Preparative thin layer chromatography (PTLC): Preparative thin layer chromatography (PTLC) was carried out on home-made glass plates (20 × 20 cm) coated with silica gel (60 M, 0.04–0.063 mm, 230–400 mesh ASTM purchased from Merck). Visualization was done by UV-light (λ = 254 and 366 nm). Column chromatography: Column chromatography was performed on silica gel (60 M, 0.04–0.063 mm/230–400 mesh ASTM purchased from Merck) and/or by using prepared solid phase extraction tubes from Mettler-Toledo Autochem (SPE-C18/18% octadecyl, 1000 mg capacity/tube, particle size: 40 µm, mean pore diameter: 6 nm). High pressure liquid chromatography (HPLC): The preparative HPLC purification was performed on an “Agilent system 1100 series” with a Phenomenex Luna 10 µm C18 (2) 100A 250 × 21.2 mm column. For detection a DAD detector was used. Column temperature: 25 °C; injection volume: 300 µL; flow: 21mL/min; gradient: 0 min 5% MeCN/H_2O [0.0059% TFA w/w]; 8 min 98% MeCN/H_2O [0.0059% TFA w/w]; maximum pressure: 200 bar. Chemicals: All chemicals were purchased from the Sigma-Aldrich

Corporation, except: (A) Riedel de Haën GmbH: 1-(But-3-yn-2-yl)-3-(4-chlorophenyl)-1-methylurea (Buturon), CAS 3766-60-7; (B) Brenntag Schweizerhall AG: 4-Azidobenzoic acid, CAS 6427-66-3. All chemicals were of analytical grade and no further purification was needed. Solvents: Commercially available solvents were used if not stated otherwise. Dry solvents were prepared by common procedures. Solid phase chemistry: All reported reactions on solid phase were carried out using a Wang Resin (Advanced ChemTech, Wang Resin SS, Bead Size: 100–200 Mesh, Polystyrene; 1% DVB, substitution: 0.9 mmol/g, Catalog#: SA5009, Lot#: 27481). Miniblock system: A Miniblock station from Mettler-Toledo Autochem was used (Bohdan 2080, New Brunswick Scientific, “Compact Shaking and Washing Station”, Shaking frequency: 450 rpm).

Synthesis of functionalized Wang resins

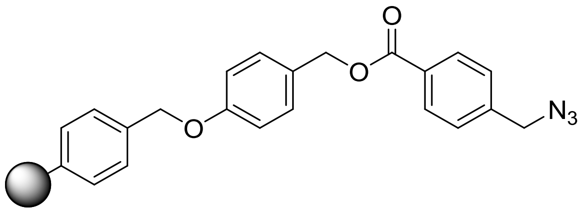

4-(Azidomethyl)benzoic acid functionalized Wang resin (7):

Resin **7** was synthesized according to **GP1**, using Wang resin (2.2 g, 1.98 mmol, 1.0 equiv), DIC (875 mg, 6.93 mmol, 3.5 equiv), DMAP (121 mg, 0.99 mmol, 0.5 equiv) and 4-(azidomethyl)benzoic acid (**6**, 877 mg, 4.95 mmol, 2.5 equiv), yielding the beige colored, quantitatively functionalized Wang resin **7** (2.55 g, 1.98 mmol, quantitative). IR (cm^{-1}) $\tilde{\nu}$: 3025 (w), 2921 (w), 2096 (m), 1716 (m), 1603 (w), 1511 (m), 1452 (m), 1269 (m), 696 (s).

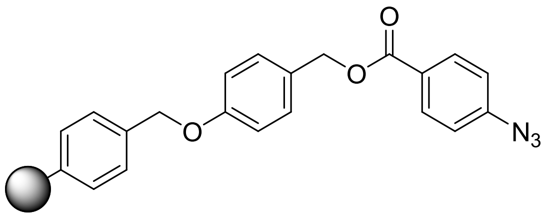

4-Azidobenzoic acid functionalized Wang resin (9):

Compound **9** was synthesized according to **GP1**, using Wang resin (2.5 g, 2.25 mmol, 1.0 equiv), DIC (994 mg, 7.88 mmol, 3.5 equiv), DMAP (137 mg, 1.13 mmol, 0.5 equiv) and 4-azidobenzoic acid (**8**, 918 mg, 5.63 mmol, 2.5 equiv), yielding the orange colored, quantitatively functionalized Wang resin **9** (2.86 g, 2.25 mmol, quantitative). IR (cm^{-1}) $\tilde{\nu}$: 3026 (w), 2920 (w), 2114 (m), 1716 (m), 1602 (m), 1512 (m), 1452 (m), 1268 (m), 697 (s).

Synthesis of substituted triazoles

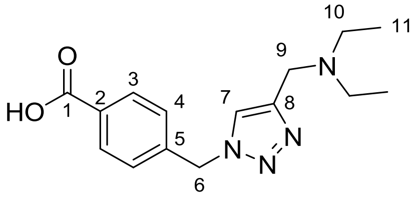

4-{[4-((Diethylamino)methyl)-1*H*-1,2,3-triazol-1-yl]methyl}benzoic acid (11a):

The compound was synthesized according to **GP 2** using 4-(azidomethyl)benzoic acid functionalized Wang resin **7** (120 mg, 93 µmol, 1.0 equiv), L-ascorbic acid (8.2 mg, 47 µmol, 0.5 equiv), copper(II) sulfate pentahydrate (2.3 mg, 9 µmol, 0.1 equiv) and *N,N*-diethylpropargylamine (**10a**, 52 µL, 373 µmol, 4.0 equiv). Cleavage according to **GP 4** yielded compound **11a** (17 mg, 59 µmol, 63%) as a light green oil.

^{1}H NMR (600 MHz, MeOD) δ (ppm) 1.35 (t, $^{3}J_{HH}$ = 7.0 Hz, 6 H, H-11), 3.18 (bs, 4 H, H-10), 4.44 (s, 2 H, H-9), 5.70 (s, 2 H, H-6); 7.38 (d, $^{3}J_{HH}$ = 7.9 Hz, 2 H, H-4), 7.79 (d, $^{3}J_{HH}$ = 7.9 Hz, 2 H, H-3), 8.25 (s, 1 H, H-7); ^{13}C NMR (150 MHz, MeOD) δ (ppm) 9.4 (+, 2 C, C-11), 46.7 (–, 2 C, C-10), 54.7 (–, 2 C, C-6, C-9), 128.1 (C_q, 1 C, C-2), 129.0 (+, 2 C, C-4), 131.3 (+, 2 C, C-3), 140.3 (C_q, 1 C, C-5), 163.2 (C_q, 1 C, C-1); HRMS–EI (m/z): [$M^{+\cdot}$] calcd for $C_{15}H_{20}N_4O_2$, 288.1586; found, 288.1586.

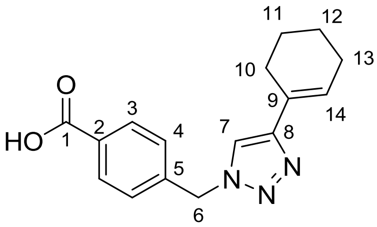

4-[Cyclohex-1-en-1-yl-1*H*-1,2,3-triazol-1-yl]methylbenzoic acid (11d):

According to **GP 2**, the resin-bound molecule **11d** was synthesized, using 4-(azidomethyl)benzoic acid functionalized Wang resin (300 mg, 233 µmol, 1.0 equiv), L-ascorbic acid (21 mg, 116 µmol, 0.5 equiv), copper(II) sulfate pentahydrate (6 mg, 23 µmol, 0.1 equiv) and 1-ethynyl-1-cyclohexanol (**10d**, 116 mg, 932 µmol, 4.0 equiv). The molecule was cleaved according to **GP 4**. The purification was done by preparative HPLC yielding compound **11d** (38 mg, 133 µmol, 57%) as a colorless solid.

Mp: 154 °C (decomposition); ^{1}H NMR (400 MHz, DMSO-d_6) δ (ppm) 1.54–1.62 (m, 2 H, H-12), 1.63–1.72 (m, 2 H, H-11), 2.09–2.18 (m, 2 H, H-13), 2.26–2.35 (m, 2 H, H-10), 5.64 (s, 2 H, H-6), 6.35–6.44 (m, 1 H, H-14), 7.35 (d, $^{3}J_{HH}$ = 8.2 Hz, 2 H, H-4), 7.92 (d, $^{3}J_{HH}$ = 8.2 Hz, 2 H, H-3), 8.16 (s, 1 H, H-7), 12.97 (bs, 1 H, COOH); ^{13}C NMR (100 MHz, DMSO-d_6) δ (ppm) 21.8 (–, 1 C, C-12), 21.9 (–, 1 C, C-11), 24.6 (–, 1 C, C-13), 25.7 (–, 1 C, C-10), 52.2 (–, 1 C,

C-6), 120.2 (+, 1 C, C-7), 123.5 (+, 1 C, C-14), 127.3 (C_q, 1 C, C-9), 127.6 (+, 2 C, C-4), 129.6 (+, 2 C, C-3), 130.4 (C_q, 1 C, C-2), 140.8 (C_q, 1 C, C-5), 148.3 (C_q, 1 C, C-8), 166.8 (C_q, 1 C, C-1); IR (cm^{-1}) $\tilde{\nu}$: 3128 (w), 2939 (w), 2875 (w), 2659 (w), 2535 (w), 1670 (s), 1424 (m), 1274 (m), 1186 (m), 1021 (m), 846 (m), 744 (s); ESIMS: m/z (%): 567.2 (30) [$2MH^+$], 325.1 (80) [MH^+ + MeCN], 284.0 (100) [MH^+].

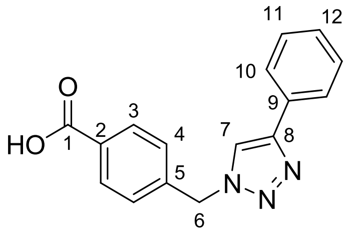

4-[(4-Phenyl-1*H*-1,2,3-triazol-1-yl)methyl]benzoic acid (11b):

Literature known compound; improved procedure [1].

According to **GP 2**, the resin-bound molecule **11b** was synthesized, using 4-(azido-methyl)benzoic acid functionalized Wang resin (180 mg, 140 µmol, 1.0 equiv), L-ascorbic acid (12 mg, 70 µmol, 0.5 equiv), copper(II) sulfate pentahydrate (3.5 mg, 14 µmol, 0.1 equiv) and phenylacetylene (**10b**, 61 µL, 559 µmol, 4.0 equiv). The molecule was cleaved according to **GP 4** and after evaporation of the solvent, compound **11b** (35 mg, 126 µmol, 90%) was obtained as a colorless solid.

Mp: 222 °C (decomposition); ^{1}H NMR (400 MHz, acetone-d_6, HSQC, HMBC): δ (ppm) 5.79 (s, 2 H, H-6), 7.31 (tt, $^3J_{HH}$ = 7.4 Hz, $^4J_{HH}$ = 1.3 Hz, 1 H, H-12), 7.41 (t, $^3J_{HH}$ = 7.6 Hz, 2 H, H-11), 7.50 (d, $^3J_{HH}$ = 8.3 Hz, 2 H, H-4), 7.88 (dt, $^3J_{HH}$ = 8.2 Hz, $^4J_{HH}$ = 1.6 Hz, 2 H, H-10), 8.05 (d, $^3J_{HH}$ = 8.3 Hz, 2 H, H-3), 8.41 (s, 1 H, H-7); ^{13}C NMR (100 MHz, acetone-d_6, HSQC, HMBC): δ (ppm) 53.9 (–, 1 C, C-6), 121.7 (+, 1 C, C-7), 126.2 (+, 2 C, C-10), 128.7 (+, 1 C, C-12), 128.8 (+, 2 C, C-4), 129.6 (+, 2 C, C-11), 131.0 (+, 2 C, C-3), 132.1 (C_q, 1 C, C-9), 141.9 (C_q, 1 C, C-5), 148.3 (C_q, 1 C, C-8), 166.9 (C_q, 1 C, C-1); IR (cm^{-1}) $\tilde{\nu}$: 3067 (w), 2920 (w), 2850 (w), 2670 (w), 2554 (w), 1681 (s), 1291 (s), 732 (s); ESIMS: m/z (%): 559.2 (25) [$2MH^+$], 321.1 (100) [MH^+ + MeCN], 280.1 (65) [MH^+].

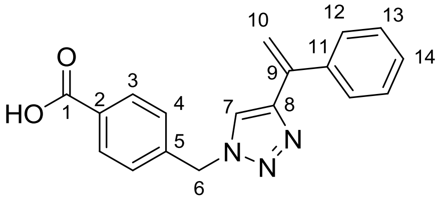

4-[1-Phenylvinyl-1*H*-1,2,3-triazol-1-yl]methylbenzoic acid (11e):

According to **GP 2**, the resin-bound molecule **11e** was synthesized, using 4-(azidomethyl)benzoic acid functionalized Wang resin (300 mg, 233 µmol, 1.0 equiv), L-ascorbic acid (21 mg, 116 µmol, 0.5 equiv), copper(II) sulfate pentahydrate (6 mg, 23 µmol,

0.1 equiv) and 2-phenyl-3-butin-2-ol (**10e**,136 mg, 932 µmol, 4.0 equiv). The molecule was cleaved according to **GP 4**. The purification was done by preparative HPLC, yielding compound **11e** (51 mg, 165 µmol, 71%) as a colorless solid.

Mp: 155 °C (decomposition); ^{1}H NMR (600 MHz, MeOD, COSY, HSQC, HMBC): δ (ppm) 5.46 (d, $^{2}J_{HH}$ = 1.1 Hz, 1 H, H-10), 5.67 (s, 2 H, H-6), 5.88 (d, $^{2}J_{HH}$ = 1.0 Hz, 1 H, H-10), 7.28–7.44 (m, 7 H, H-4, H-12, H-13, H-14), 7.91 (s, 1 H, H-7), 8.02 (d, $^{3}J_{HH}$ = 8.4 Hz, 2 H, H-3); ^{13}C NMR (150 MHz, MeOD, COSY, HSQC, HMBC): δ (ppm) 54.4 (–, 1 C, C-6), 115.6 (–, 1 C, C-10), 124.5 (+, 1 C, C-7), 128.7 (+, 2 C, C-12), 128.9 (+, 2 C, C-4), 129.3 (+, 1 C, C-14), 129.6 (+, 2 C, C-13), 131.4 (+, 2 C, C-3), 132.1 (C_q, 1 C, C-2), 140.5 (C_q, 1 C, C-11), 141.1 (C_q, 1 C, C-9), 141.8 (C_q, 1 C, C-5), 149.3 (C_q, 1 C, C-8), 169.2 (C_q, 1 C, C-1); IR (cm^{-1}) $\tilde{\nu}$: 3131 (w), 2915 (w), 2848 (w), 2673 (w), 2565 (w), 1686 (s), 1430 (m), 1294 (s), 1049 (m), 730 (s), 698 (s); HRMS–EI (M/Z): $[M]^{+}$ calcd for $C_{18}H_{15}N_3O_2$, 305.1164; found, 305.1165.

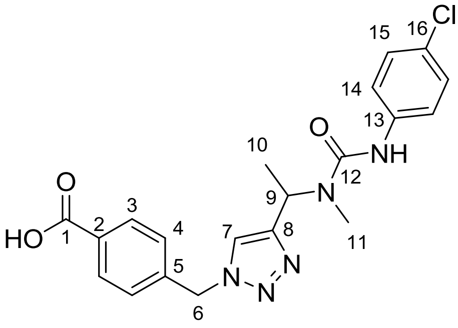

4-{[4-(1-(3-(4-Chlorophenyl)-1-methylureido)ethyl)-1*H*-1,2,3-triazol-1-yl]methyl}benzoic acid (11c):

According to **GP 2**, the resin-bound molecule **11c** was synthesized, using 4-(azidomethyl)benzoic acid functionalized Wang resin (150 mg, 116 µmol, 1.0 equiv), L-ascorbic acid (10 mg, 58 µmol, 0.5 equiv), copper(II) sulfate pentahydrate (3 mg, 12 µmol, 0.1 equiv) and 1-(but-3-yn-2-yl)-3-(4-chlorophenyl)-1-methylurea (**10c**, 110 mg, 466 µmol, 4.0 equiv). The molecule was cleaved according to **GP 4**. The purification was done by preparative HPLC, yielding compound **11c** (39 mg, 94 µmol, 81%) as a colorless solid.

Mp: 183–189 °C; ^{1}H NMR (400 MHz, DMSO-d_6) δ (ppm) 1.47 (d, $^{3}J_{HH}$ = 7.0 Hz, 3 H, H-10), 2.72 (s, 3 H, H-11), 5.66 (q, $^{3}J_{HH}$ = 7.0 Hz, 1 H, H-9), 5.67 (s, 2 H, H-6), 7.28 (d, $^{3}J_{HH}$ = 8.9 Hz, 2 H, H-15), 7.39 (d, $^{3}J_{HH}$ = 8.0 Hz, 2 H, H-4), 7.54 (d, $^{3}J_{HH}$ = 8.9 Hz, 2 H, H-14), 7.94 (d, $^{3}J_{HH}$ = 8.0 Hz, 2 H, H-3), 8.14 (s, 1 H, NH), 8.46 (s, 1 H, H-7), 12.99 (bs, 1 H, COOH); ^{13}C NMR (100 MHz, DMSO-d_6) δ (ppm) 16.8 (+, 1 C, C-10), 28.7 (+, 1 C, C-11), 45.7 (+, 1 C, C-9), 52.3 (–, 1 C, C-6), 121.2 (+, 2 C, C-14), 123.1 (+, 1 C, C-7), 125.2 (C_q, 1 C, C-16), 127.8 (+, 2 C, C-4), 127.9 (+, 2 C, C-15), 128.0 (+, 2 C, C-3), 139.5 (C_q, 1 C, C-13),

140.8 (C_q, 1 C, C-5), 147.7 (C_q, 1 C, C-8), 155.1 (C_q, 1 C, C-12), 166.8 (C_q, 1 C, C-1); IR (cm^{-1}) $\tilde{\nu}$: 3112 (w), 2983 (w), 2922 (w), 2255 (w), 1647 (m), 1493 (m), 1402 (m), 1242 (m), 1023 (s), 824 (m), 647 (m); ESIMS: m/z (%): 827.4 (20) [$2MH^+$], 414.0 (100) [MH^+].

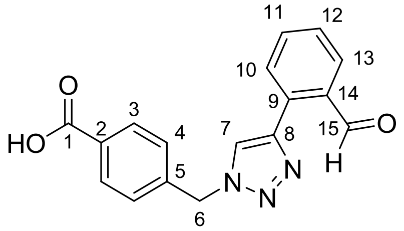

4-{[4-(2-Formylphenyl)-1*H*-1,2,3-triazol-1-yl]methyl}benzoic acid (11f):

According to **GP 2**, the resin-bound molecule **11f** was synthesized, using 4-(azidomethyl)benzoic acid functionalized Wang resin (100 mg, 78 µmol, 1.0 equiv), L-ascorbic acid (6.9 mg, 39 µmol, 0.5 equiv), copper(II) sulfate pentahydrate (2.0 mg, 8 µmol, 0.1 equiv) and 2-ethynylbenzaldehyde (**10f**, 40 mg, 311 µmol, 4.0 equiv). Cleavage was done according to **GP 4**, followed by purification using a SPE tube. Subsequently, the solvent was evaporated, yielding compound **11f** (23 mg, 74 µmol, 95%) as a light yellow solid.

Mp: 144 °C (decomposition); ^{1}H NMR (400 MHz, DMSO-d_6) δ (ppm) 5.80 (s, 2 H, H-6), 7.47 (d, $^3J_{HH}$ = 8.2 Hz, 2 H, H-4), 7.56 (td, $^3J_{HH}$ = 7.2 Hz, $^4J_{HH}$ = 1.0 Hz, 1 H, H-12), 7.74 (td, $^3J_{HH}$ = 7.7 Hz, $^4J_{HH}$ = 1.0 Hz, 1 H, H-11), 7.78 (dd, $^3J_{HH}$ = 7.7 Hz, $^4J_{HH}$ = 1.3 Hz, 1 H, H-13), 7.89 (dd, $^3J_{HH}$ = 7.7 Hz, $^4J_{HH}$ = 1.0 Hz, 2 H, H-10), 7.96 (d, $^3J_{HH}$ = 8.2 Hz, 2 H, H-3), 8.78 (s, 1 H, H-7), 10.34 (s, 1 H, H-15), 13.03 (bs, 1 H, COOH); ^{13}C NMR (100 MHz, DMSO-d_6) δ (ppm) 52.5 (–, 1 C, C-6), 124.9 (+, 1 C, C-7), 127.4 (+, 1 C, C-10), 127.8 (+, 2 C, C-4), 128.3 (+, 1 C, C-13), 128.5 (+, 1 C, C-12), 129.6 (+, 2 H, C-3), 132.7 (C_q, 1 C, C-2), 133.2 (C_q, 1 C, C-9), 133.7 (+, 1 C, C-11), 138.1 (C_q, 1 C, C-14), 140.3 (C_q, 1 C, C-5), 144.0 (C_q, 1 C, C-8), 166.7 (C_q, 1 C, C-1), 192.3 (+, 1 C, C-15); IR (cm^{-1}) $\tilde{\nu}$: 3141 (w), 3014 (w), 2884 (w), 2809 (w), 1675 (s), 1602 (m), 1400 (m), 1198 (m), 759 (s), 739 (s); HRMS (ESI+): [MH^+] calcd for $C_{17}H_{14}N_3O_3$, 308.1030; found, 308.1027.

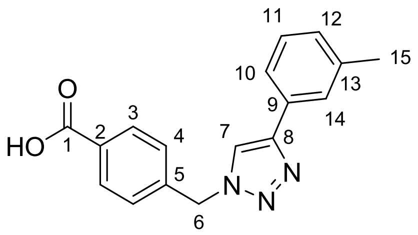

4-[(4-m-Tolyl-1*H*-1,2,3-triazol-1-yl)methyl]benzoic acid (11g):

According to **GP 2**, the resin-bound molecule **11g** was synthesized, using 4-(azidomethyl)benzoic acid functionalized Wang resin (100 mg, 78 µmol, 1.0 equiv), L-ascorbic acid (6.9 mg, 39 µmol, 0.5 equiv), copper(II) sulfate pentahydrate (2.0 mg, 8 µmol, 0.1 equiv) and 3-ethynyltoluene (**10g**, 40 µL, 311 µmol, 4.0 equiv). Cleavage was done

according to **GP 4**, followed by purification using a SPE tube. Subsequently, the solvent was evaporated, yielding compound **11g** (23 mg, 77 µmol, 99%) as a colorless solid.

Mp: 138 °C (decomposition); ^{1}H NMR (400 MHz, $CDCl_3$, HSQC, HMBC) δ (ppm) 2.37 (s, 3 H, H-15), 5.65 (s, 2 H, H-6), 7.14 (d, $^{3}J_{HH}$ = 7.6 Hz, 1 H, H-12), 7.29 (t, $^{3}J_{HH}$ = 7.7 Hz, 1 H, H-11), 7.36 (d, $^{3}J_{HH}$ = 8.3 Hz, 2 H, H-4), 7.57 (d, $^{3}J_{HH}$ = 7.7 Hz, 1 H, H-10), 7.65 (s, 1 H, H-14), 7.70 (s, 1 H, H-7), 8.09 (d, $^{3}J_{HH}$ = 8.3 Hz, 2 H, H-3); ^{13}C NMR (100 MHz, $CDCl_3$, HSQC, HMBC) δ (ppm) 21.4 (+, 1 C, C-15), 53.7 (–, 1 C, C-6), 119.6 (+, 1 C, C-7), 122.8 (+, 1 C, C-10), 126.4 (+, 1 C, C-14), 127.8 (+, 2 C, C-4), 128.7 (+, 1 C, C-11), 129.1 (+, 1 C, C-12), 130.0 (C_q, 1 C, C-2), 130.9 (+, 2 C, C-3), 138.5 (C_q, 1 C, C-13), 140.1 (C_q, 1 C, C-5), 169.5 (C_q, 1 C, C-1); IR (cm^{-1}) ṽ: 3134 (w), 2949 (w), 2884 (w), 2675 (w), 1687 (m), 1282 (m), 779 (s), 725 (s), 690 (s); HRMS (ESI+): [MH^{+}] calcd for $C_{17}H_{16}N_3O_2$, 294.1237; found, 294.1245.

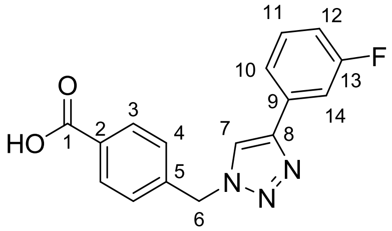

4-{[4-(3-Fluorophenyl)-1*H*-1,2,3-triazol-1-yl]methyl}benzoic acid (11h):

According to **GP 2**, the resin-bound molecule **11h** was synthesized, using 4-(azidomethyl)benzoic acid functionalized Wang resin (100 mg, 78 µmol, 1.0 equiv), L-ascorbic acid (6.9 mg, 39 µmol, 0.5 equiv), copper(II) sulfate pentahydrate (2.0 mg, 8 µmol, 0.1 equiv) and 1-ethynyl-3-fluorobenzene (**10h**, 36 µL, 311 µmol, 4.0 equiv). Cleavage was done according to **GP 4**, followed by purification using a SPE tube. Subsequently, the solvent was evaporated, yielding compound **11h** (22 mg, 75 µmol, 97%) as a colorless solid.

Mp: 174 °C (decomposition); ^{1}H NMR (400 MHz, DMSO-d_6) δ (ppm) 5.76 (s, 2 H, H-6), 7.16 (dddd, $^{3}J_{HF}$ = 8.9 Hz, $^{3}J_{HH}$ = 8.0 Hz, $^{4}J_{HH}$ = 2.6 Hz, $^{4}J_{HH}$ = 0.8 Hz, 1 H, H-12), 7.43 (d, $^{3}J_{HH}$ = 8.4 Hz, 2 H, H-4), 7.49 (ddd, $^{3}J_{HH}$ = 8.0 Hz, $^{3}J_{HH}$ = 7.9 Hz, $^{4}J_{HF}$ = 6.2 Hz, 1 H, H-11), 7.66 (ddd, $^{3}J_{HF}$ = 10.4 Hz, $^{4}J_{HH}$ = 2.5 Hz, $^{4}J_{HH}$ = 1.5 Hz, 1 H, H-14), 7.71 (ddd, $^{3}J_{HH}$ = 7.8 Hz, $^{4}J_{HH}$ = 1.1 Hz, $^{4}J_{HH}$ = 0.9 Hz, 1 H, H-10), 7.96 (d, $^{3}J_{HH}$ = 8.3 Hz, 2 H, H-3), 8.74 (s, 1 H, H-7), 12.92 (bs, 1 H, COOH); ^{13}C NMR (100 MHz, DMSO-d_6) δ (ppm) 52.8 (–, 1 C, C-6), 111.9 (+, d, $^{2}J_{CF}$ = 23.0 Hz, 1 C, C-12), 114.8 (+, d, $^{2}J_{CF}$ = 21.0 Hz, 1 C, C-14), 121.3 (+, d, $^{4}J_{CF}$ = 2.6 Hz, 1 C, C-10), 122.7 (+, 1 C, C-7), 128.1 (+, 2 C, C-4), 129.9 (+, 2 H, C-3), 130.7 (C_q, 1 C, C-2), 131.2 (+, d, $^{3}J_{CF}$ = 8.4 Hz, 1 C, C-11), 133.1 (C_q, d, $^{3}J_{CF}$ = 8.4 Hz, 1 C, C-9), 140.7 (C_q, 1 C, C-5), 145.8 (C_q, d, $^{4}J_{CF}$ = 2.9 Hz, 1 C, C-8), 162.7 (C_q, d, $^{1}J_{CF}$ = 242.9 Hz, 1 C, C-13), 167.0 (C_q, 1 C, C-1); IR (cm^{-1}) ṽ: 3134 (w), 2956 (w), 2922 (w), 2849 (w), 2680 (w),

1682 (s), 1428 (m), 1295 (s), 862 (s), 784 (s), 730 (s); HRMS (ESI+): [MH^+] calcd for $C_{16}H_{13}FN_3O_2$, 298.0986; found, 298.0990.

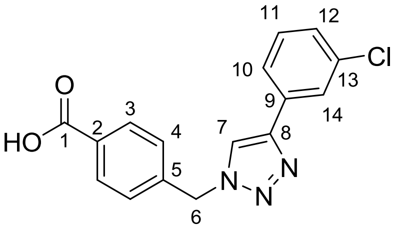

4-{[4-(3-Chlorophenyl)-1*H*-1,2,3-triazol-1-yl]methyl}benzoic acid (11i):

According to **GP 2**, the resin-bound molecule **11i** was synthesized, using 4-(azidomethyl)benzoic acid functionalized Wang resin (100 mg, 78 µmol, 1.0 equiv), L-ascorbic acid (6.9 mg, 39 µmol, 0.5 equiv), copper(II) sulfate pentahydrate (2.0 mg, 8 µmol, 0.1 equiv) and 3-chloro-1-ethynylbenzene (**10i**, 38 µL, 311 µmol, 4.0 equiv). Cleavage was done according to **GP 4**, followed by purification using a SPE tube. Subsequently, the solvent was evaporated, yielding compound **11i** (24 mg, 76 µmol, 98%) as a light-beige solid.

Mp: 168 °C (decomposition); ^{1}H NMR (400 MHz, DMSO-d_6, HSQC, HMBC) δ (ppm) 5.76 (s, 2 H, H-6), 7.39 (ddd, $^3J_{HH}$ = 8.0 Hz, $^4J_{HH}$ = 2.1 Hz, $^4J_{HH}$ = 1.0 Hz, 1 H, H-12), 7.44 (d, $^3J_{HH}$ = 8.3 Hz, 2 H, H-4), 7.47 (t, $^3J_{HH}$ = 7.9 Hz, 1 H, H-11), 7.84 (ddd, $^3J_{HH}$ = 7.8 Hz, $^4J_{HH}$ = 1.3 Hz, $^4J_{HH}$ = 1.2 Hz, 1 H, H-10), 7.91 (t, $^4J_{HH}$ = 1.8 Hz, 1 H, H-14), 7.96 (d, $^3J_{HH}$ = 8.3 Hz, 2 H, H-3), 8.77 (s, 1 H, H-7), 13.04 (bs, 1 H, COOH); ^{13}C NMR (100 MHz, DMSO-d_6, HSQC, HMBC) δ (ppm) 52.6 (–, 1 C, C-6), 122.5 (+, 1 C, C-7), 123.6 (+, 1 C, C-10), 124.7 (+, 1 C, C-14), 127.6 (+, 1 C, C-12), 127.9 (+, 2 C, C-4), 129.7 (+, 2 C, C-3), 130.5 (C_q, 1 C, C-2), 130.8 (+, 1 C, C-11), 132.6 (C_q, 1 C, C-9), 133.6 (C_q, 1 C, C-13), 140.4 (C_q, 1 C, C-5), 145.3 (C_q, 1 C, C-8), 166.8 (C_q, 1 C, C-1); IR (cm^{-1}) $\tilde{\nu}$: 3134 (w), 3081 (w), 2953 (w), 1687 (m), 1281 (m), 871 (m), 779 (s), 725 (s), 690 (s); HRMS (ESI+): [MH^+] calcd for $C_{16}H_{13}ClN_3O_2$, 314.0691; found, 314.0672; MF: $C_{16}H_{12}ClN_3O_2$; MW: 313.74

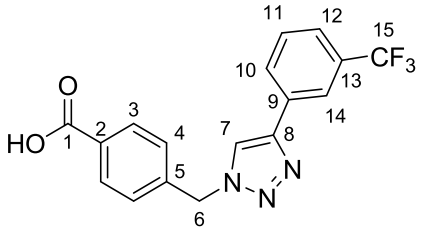

4-{[4-(3-(Trifluoromethyl)phenyl)-1*H*-1,2,3-triazol-1-yl]methyl}benzoic acid (11j):

According to **GP 2**, the resin-bound molecule **11j** was synthesized, using 4-(azidomethyl)benzoic acid functionalized Wang resin (100 mg, 78 µmol, 1.0 equiv), L-ascorbic acid (6.9 mg, 39 µmol, 0.5 equiv), copper(II) sulfate pentahydrate (2.0 mg, 8 µmol, 0.1 equiv) and 3-ethynyl-α,α,α-trifluorotoluene (**10j**, 45 µL, 311 µmol, 4.0 equiv). Cleavage

was done according to **GP 4**, followed by purification using a SPE tube. Subsequently, the solvent was evaporated, yielding compound **11j** (26 mg, 76 µmol, 98%) as a colorless solid.

Mp: 124 °C; ^{1}H NMR (400 MHz, DMSO-d_6) δ (ppm) 5.78 (s, 2 H, H-6), 7.45 (d, $^{3}J_{HH}$ = 8.4 Hz, 2 H, H-4), 7.65–7.73 (m, 2 H, H-11, H-12), 7.96 (d, $^{3}J_{HH}$ = 8.3 Hz, 2 H, H-3), 8.14–8.22 (m, 2 H, H-10, H-14), 8.87 (s, 1 H, H-7), 12.96 (bs, 1 H, COOH); ^{13}C NMR (100 MHz, DMSO-d_6) δ (ppm) 52.7 (–, 1 C, C-6), 121.5 (+, d, $^{3}J_{CF}$ = 3.7 Hz, 1 C, C-12), 122.7 (+, 1 C, C-7), 124.3 (+, d, $^{3}J_{CF}$ = 3.5 Hz, 1 C, C-14), 125.4 (C_q, 1 C, C-15), 127.9 (+, 2 C, C-4), 128.9 (+, 1 C, C-11), 129.7 (C_q, d, $^{2}J_{CF}$ = 31.7 Hz, 1 C, C-13), 129.8 (+, 2 C, C-3), 130.1 (+, 1 C, C-10), 130.5 (C_q, 1 C, C-2), 131.6 (C_q, 1 C, C-9), 140.4 (C_q, 1 C, C-5), 145.3 (C_q, 1 C, C-8), 166.8 (C_q, 1 C, C-1); IR (cm^{-1}) ṽ: 3080 (w), 2850 (w), 2556 (w), 1682 (s), 1429 (m), 1294 (s), 1175 (m), 1126 (s), 1069 (m), 807 (m), 729 (s), 697 (s); HRMS (ESI+): [MH^+] calcd for $C_{17}H_{13}F_3N_3O_2$, 348.0954; found, 348.0968.

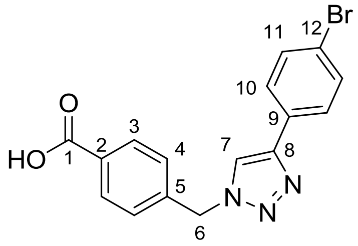

4-{[4-(4-Bromophenyl)-1*H*-1,2,3-triazol-1-yl]methyl}benzoic acid (11k):

According to **GP 2**, the resin-bound molecule **11k** was synthesized, using 4-(azidomethyl)benzoic acid functionalized Wang resin (100 mg, 78 µmol, 1.0 equiv), L-ascorbic acid (6.9 mg, 39 µmol, 0.5 equiv), copper(II) sulfate pentahydrate (2.0 mg, 8 µmol, 0.1 equiv) and 1-bromo-4-ethynylbenzene (**10k**, 56 mg, 311 µmol, 4.0 equiv). Cleavage was done according to **GP 4**, followed by purification using a SPE tube. Subsequently, the solvent was evaporated, yielding compound **11k** (27 mg, 75 µmol, 97%) as a colorless solid.

Mp: 245 °C; ^{1}H NMR (400 MHz, DMSO-d_6) δ (ppm) 5.75 (s, 2 H, H-6), 7.43 (d, $^{3}J_{HH}$ = 8.2 Hz, 2 H, H-4), 7.63 (d, $^{3}J_{HH}$ = 8.5 Hz, 2 H, H-11), 7.81 (d, $^{3}J_{HH}$ = 8.5 Hz, 2 H, H-10), 7.95 (d, $^{3}J_{HH}$ = 8.2 Hz, 2 H, H-3), 8.71 (s, 1 H, H-7); ^{13}C NMR (100 MHz, DMSO-d_6) δ (ppm) 52.5 (–, 1 C, C-6), 120.7 (C_q, 1 C, C-12), 122.0 (+, 1 C, C-7), 127.0 (+, 2 C, C-10), 127.8 (+, 2 C, C-4), 129.6 (+, 2 C, C-3), 129.7 (C_q, 1 C, C-9), 130.4 (C_q, 1 C, C-2), 131.7 (+, 2 C, C-11), 140.4 (C_q, 1 C, C-5), 145.5 (C_q, 1 C, C-8), 166.7 (C_q, 1 C, C-1); IR (cm^{-1}) ṽ: 3114 (w), 3083 (w), 2857 (w), 2664 (w), 2544 (w), 1673 (s), 1423 (m), 1283 (s), 1185 (m), 818 (s), 714 (s); HRMS (ESI+): [MH^+] calcd for $C_{16}H_{13}BrN_3O_2$, 359.0186; found, 359.0198.

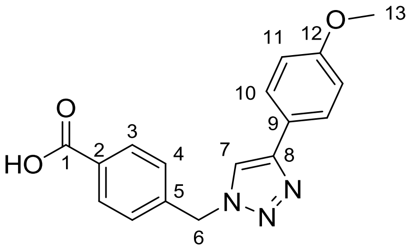

4-{[4-(4-Methoxyphenyl)-1*H*-1,2,3-triazol-1-yl]methyl}benzoic acid (11l):
According to **GP 2**, the resin-bound molecule **11l** was synthesized, using 4-(azidomethyl)benzoic acid functionalized Wang resin (100 mg, 78 µmol, 1.0 equiv), L-ascorbic acid (6.9 mg, 39 µmol, 0.5 equiv), copper(II) sulfate pentahydrate (2.0 mg, 8 µmol, 0.1 equiv) and 4-ethynylanisole (**10l**, 40 µL, 311 µmol, 4.0 equiv). Cleavage was done according to **GP 4**, followed by purification using a SPE tube. After evaporation of the solvent, compound **11l** (24 mg, 76 µmol, 98%) was yielded as a colorless solid.

Mp: 238 °C (decomposition); ^{1}H NMR (400 MHz, DMSO-d_6) δ (ppm) 3.77 (s, 3 H, H-13), 5.72 (s, 2 H, H-6), 7.00 (d, $^{3}J_{HH}$ = 8.8 Hz, 2 H, H-11), 7.42 (d, $^{3}J_{HH}$ = 8.3 Hz, 2 H, H-4), 7.77 (d, $^{3}J_{HH}$ = 8.8 Hz, 2 H, H-10), 7.95 (d, $^{3}J_{HH}$ = 8.3 Hz, 2 H, H-3), 8.54 (s, 1 H, H-7); ^{13}C NMR (100 MHz, DMSO-d_6) δ (ppm) 52.5 (–, 1 C, C-6), 55.1 (+, 1 C, C-13), 114.3 (+, 2 C, C-11), 120.8 (+, 1 C, C-7), 123.1 (C_q, 1 C, C-9), 126.5 (+, 2 C, C-10), 127.9 (+, 2 C, C-4), 129.8 (+, 2 C, C-3), 130.5 (C_q, 1 C, C-2), 140.8 (C_q, 1 C, C-5), 146.7 (C_q, 1 C, C-8), 159.0 (C_q, 1 C, C-12), 166.9 (C_q, 1 C, C-1); IR (cm^{-1}) $\tilde{\nu}$: 3103 (w), 2847 (w), 2672 (w), 2545 (w), 1682 (s), 1498 (m), 1428 (m), 1245 (s), 1173 (m), 1024 (s), 818 (s), 726 (s); HRMS (ESI+): [MH$^+$] calcd for $C_{17}H_{16}N_3O_3$, 310.1186; found, 310.1165.

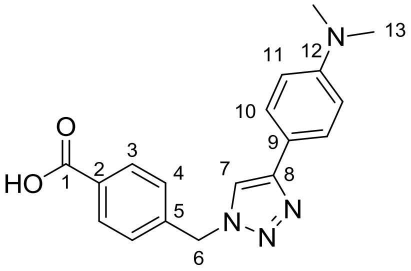

4-{[4-(4-(Dimethylamino)phenyl)-1*H*-1,2,3-triazol-1-yl]methyl}benzoic acid (11m):
According to **GP 2**, the resin-bound molecule **11m** was synthesized, using 4-(azidomethyl)benzoic acid functionalized Wang resin (100 mg, 78 µmol, 1.0 equiv), L-ascorbic acid (6.9 mg, 39 µmol, 0.5 equiv), copper(II) sulfate pentahydrate (2.0 mg, 8 µmol, 0.1 equiv) and 4-ethynyl-*N*,*N*-dimethylaniline (**10m**, 45 mg, 311 µmol, 4.0 equiv). Cleavage was done according to **GP 4**, followed by purification using a SPE tube. After evaporation of the solvent, compound **11m** (22 mg, 68 µmol, 88%) was yielded as a dark red solid.

Mp: 154 °C (decomposition); ^{1}H NMR (400 MHz, DMSO-d_6) δ (ppm) 2.96 (s, 6 H, H-13), 5.70 (s, 2 H, H-6), 6.90 (d, $^3J_{HH}$ = 8.4 Hz, 2 H, H-11), 7.41 (d, $^3J_{HH}$ = 8.1 Hz, 2 H, H-4), 7.70 (d, $^3J_{HH}$ = 8.5 Hz, 2 H, H-10), 7.94 (d, $^3J_{HH}$ = 8.1 Hz, 2 H, H-3), 8.48 (s, 1 H, H-7); ^{13}C NMR (100 MHz, DMSO-d_6) δ (ppm) 40.6 (+, 2 C, C-13), 52.3 (–, 1 C, C-6), 113.4 (+, 2 C, C-11), 120.1 (+, 1 C, C-7), 126.0 (+, 2 C, C-10), 127.7 (+, 2 C, C-4), 128.8 (C_q, 1 C, C-9), 129.6 (+, 2 C, C-3), 130.3 (C_q, 1 C, C-2), 140.7 (C_q, 1 C, C-5), 148.8 (C_q, 1 C, C-8), 166.7 (C_q, 1 C, C-1); IR (cm^{-1}) $\tilde{\nu}$: 3131 (w), 3077 (w), 2884 (w), 2676 (w), 1687 (m), 1573 (m), 1282 (m), 1098 (m), 780 (s), 691 (s); MS (ESI+, TOF) m/z (%) 323.1 (100) [MH$^+$].

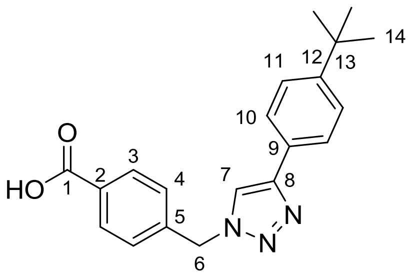

4-{[4-(4-*tert*-Butylphenyl)-1*H*-1,2,3-triazol-1-yl]methyl}benzoic acid (11n):

According to **GP 2**, the resin-bound molecule **11n** was synthesized, using 4-(azidomethyl)benzoic acid functionalized Wang resin (100 mg, 78 µmol, 1.0 equiv), L-ascorbic acid (6.9 mg, 39 µmol, 0.5 equiv), copper(II) sulfate pentahydrate (2.0 mg, 8 µmol, 0.1 equiv) and 4-*tert*-butylphenylacetylene (**10n**, 56 µL, 311 µmol, 4.0 equiv). Cleavage was done according to **GP 4**, followed by purification using a SPE tube. After evaporation of the solvent, compound **11n** (26 mg, 76 µmol, 98%) was yielded as a colorless solid.

Mp: 243 °C (decomposition); ^{1}H NMR (400 MHz, $CDCl_3$, HSQC, HMBC) δ (ppm) 1.31 (s, 9 H, H-14), 5.62 (s, 2 H, H-6), 7.33 (d, $^3J_{HH}$ = 8.2 Hz, 2 H, H-4), 7.42 (d, $^3J_{HH}$ = 8.4 Hz, 2 H, H-11), 7.69 (s, 1 H, H-7), 7.71 (d, $^3J_{HH}$ = 8.4 Hz, 2 H, H-10), 8.06 (d, $^3J_{HH}$ = 8.2 Hz, 2 H, H-3), 11.84 (bs, 1 H, COOH); ^{13}C NMR (100 MHz, $CDCl_3$, HSQC, HMBC) δ (ppm) 31.2 (+, 3 C, C-14), 34.6 (C_q, 1 C, C-13), 53.8 (–, 1 C, C-6), 119.6 (+, 1 C, C-7), 125.5 (+, 2 C, C-10), 125.8 (+, 2 C, C-11), 127.1 (C_q, 1 C, C-9), 127.7 (+, 2 C, C-4), 130.7 (+, 2 C, C-3), 130.9 (C_q, 1 C, C-2), 139.5 (C_q, 1 C, C-5), 148.4 (C_q, 1 C, C-8), 151.5 (C_q, 1 C, C-12), 168.6 (C_q, 1 C, C-1); IR (cm^{-1}) $\tilde{\nu}$: 2960 (w), 2927 (w), 2671 (w), 2545 (w), 1685 (s), 1424 (m), 1283 (m), 1188 (m), 1021 (m), 835 (m), 745 (s); MS (ESI+, TOF) m/z (%) 671.3 (15) [2MH$^+$], 336.1 (100) [MH$^+$].

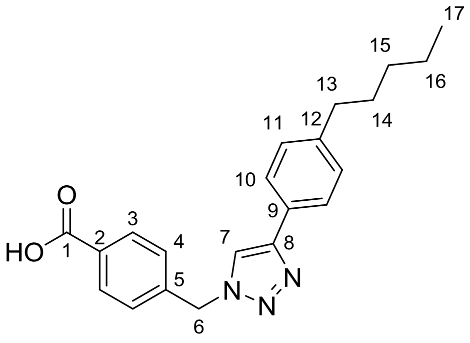

4-{[4-(4-Pentylphenyl)-1*H*-1,2,3-triazol-1-yl]methyl}benzoic acid (11o):

According to **GP 2**, the resin-bound molecule **11o** was synthesized, using 4-(azidomethyl)benzoic acid functionalized Wang resin (100 mg, 78 µmol, 1.0 equiv), L-ascorbic acid (6.9 mg, 39 µmol, 0.5 equiv), copper(II) sulfate pentahydrate (2.0 mg, 8 µmol, 0.1 equiv) and 1-ethynyl-4-pentylbenzene (**10o**, 61 µL, 311 µmol, 4.0 equiv). Cleavage was done according to **GP 4**, followed by purification using a SPE tube. After evaporation of the solvent, compound **11o** (26 mg, 75 µmol, 97%) was yielded as a colorless solid.

Mp: 169 °C (decomposition); ^{1}H NMR (400 MHz, DMSO-d_6, HSQC, HMBC) δ (ppm) 0.84 (t, $^{3}J_{HH}$ = 7.0 Hz, 3 H, H-17), 1.20–1.33 (m, 4 H, H-15, H-16), 1.56 (quintet, $^{3}J_{HH}$ = 7.5 Hz, 2 H, H-14), 2.57 (t, $^{3}J_{HH}$ = 7.8 Hz, 2 H, H-13), 5.73 (s, 2 H, H-6), 7.24 (d, $^{3}J_{HH}$ = 8.2 Hz, 2 H, H-11), 7.42 (d, $^{3}J_{HH}$ = 8.3 Hz, 2 H, H-4), 7.74 (d, $^{3}J_{HH}$ = 8.2 Hz, 2 H, H-10), 7.95 (d, $^{3}J_{HH}$ = 8.3 Hz, 2 H, H-3), 8.59 (s, 1 H, H-7); ^{13}C NMR (100 MHz, DMSO-d_6, HSQC, HMBC) δ (ppm) 13.9 (+, 1 C, C-17), 21.9 (–, 1 C, C-16), 30.5 (–, 1 C, C-15), 30.8 (–, 1 C, C-14), 34.8 (–, 1 C, C-13), 52.6 (–, 1 C, C-6), 121.4 (+, 1 C, C-7), 125.1 (+, 2 C, C-10), 127.9 (+, 2 C, C-4), 128.0 (C_q, 1 C, C-9), 128.8 (+, 2 C, C-11), 129.8 (+, 2 C, C-3), 130.5 (C_q, 1 C, C-2), 140.8 (C_q, 1 C, C-5), 142.2 (C_q, 1 C, C-12), 146.8 (C_q, 1 C, C-8), 166.9 (C_q, 1 C, C-1); IR (cm^{-1}) $\tilde{\nu}$: 2959 (m), 2927 (m), 2857 (m), 2671 (w), 2555 (w), 1685 (s), 1424 (m), 1284 (m), 1188 (m), 1049 (m), 836 (m), 745 (s); MS MS (ESI+, TOF) m/z (%) 699.4 (15) [2MH^{+}], 350.2 (100) [MH^{+}].

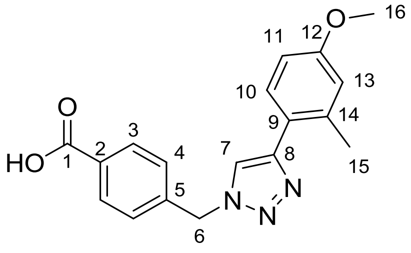

4-{[4-(4-Methoxy-2-methylphenyl)-1*H*-1,2,3-triazol-1-yl]methyl}benzoic acid (11p):

According to **GP 2**, the resin-bound molecule **11p** was synthesized, using 4-(azidomethyl)benzoic acid functionalized Wang resin (100 mg, 78 µmol, 1.0 equiv), L-ascorbic acid (6.9 mg, 39 µmol, 0.5 equiv), copper(II) sulfate pentahydrate (2.0 mg, 8 µmol,

0.1 equiv) and 1-ethynyl-4-methoxy-2-methylbenzene (**10p**, 45 mg, 311 µmol, 4.0 equiv). Cleavage was done according to **GP 4**, followed by purification using a SPE tube. Subsequently, the solvent was evaporated, yielding compound **11p** (25 mg, 77 µmol, 99%) as a colorless solid.

Mp: 146 °C (decomposition); ^{1}H NMR (400 MHz, DMSO-d_6) δ (ppm) 2.40 (s, 3 H, H-15), 3.77 (s, 3 H, H-16), 5.74 (s, 2 H, H-6), 6.85 (d, $^{3}J_{HH}$ = 8.4 Hz, $^{4}J_{HH}$ = 2.7 Hz, 1 H, H-11), 6.87 (d, $^{4}J_{HH}$ = 2.5 Hz, 1 H, H-13), 7.43 (d, $^{3}J_{HH}$ = 8.3 Hz, 2 H, H-4), 7.66 (d, $^{3}J_{HH}$ = 8.4 Hz, 1 H, H-10), 7.95 (d, $^{3}J_{HH}$ = 8.3 Hz, 2 H, H-3), 8.42 (s, 1 H, H-7), 13.02 (bs, 1 H, COOH); ^{13}C NMR (100 MHz, DMSO-d_6) δ (ppm) 21.2 (+, 1 C, C-15), 52.3 (–, 1 C, C-6), 55.0 (+, 1 C, C-16), 111.5 (+, 1 C, C-11), 115.9 (+, 1 C, C-13), 122.4 (C_q, 1 C, C-9), 122.9 (+, 1 C, C-7), 127.7 (+, 2 C, C-4), 129.4 (+, 1 C, C-10), 129.7 (+, 2 C, C-3), 130.4 (C_q, 1 C, C-2), 136.4 (C_q, 1 C, C-14), 140.9 (C_q, 1 C, C-5), 145.8 (C_q, 1 C, C-8), 158.7 (C_q, 1 C, C-12), 166.8 (C_q, 1 C, C-1); IR (cm^{-1}) ṽ: 3122 (w), 3090 (w), 3002 (w), 2836 (w), 2675 (w), 2552 (w), 1686 (s), 1425 (m), 1287 (m), 729 (s); MS (ESI+, TOF) m/z (%) 647.3 (5) [2MH$^+$], 324.1 (100) [MH$^+$].

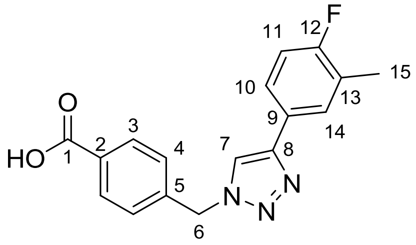

4-{[4-(4-Fluoro-3-methylphenyl)-1*H*-1,2,3-triazol-1-yl]methyl}benzoic acid (11q):
According to **GP 2**, the resin-bound molecule **11q** was synthesized, using 4-(azidomethyl)benzoic acid functionalized Wang resin (100 mg, 78 µmol, 1.0 equiv), L-ascorbic acid (6.9 mg, 39 µmol, 0.5 equiv), copper(II) sulfate pentahydrate (2.0 mg, 8 µmol, 0.1 equiv) and 4-ethynyl-1-fluoro-2-methylbenzene (**10q**, 41 µL, 311 µmol, 4.0 equiv). Cleavage was done according to **GP 4**, followed by purification using a SPE tube. Subsequently, the solvent was evaporated, yielding compound **11q** (24 mg, 76 µmol, 98%) as a brown solid.

Mp: 153 °C (decomposition); ^{1}H NMR (400 MHz, DMSO-d_6) δ (ppm) 2.27 (d, $^{4}J_{HF}$ = 1.6 Hz, 3 H, H-15), 5.73 (s, 2 H, H-6), 7.20 (dd, $^{3}J_{HF}$ = 8.9 Hz, $^{3}J_{HH}$ = 8.7 Hz, 1 H, H-11), 7.42 (d, $^{3}J_{HH}$ = 8.3 Hz, 2 H, H-4), 7.69 (ddd, $^{3}J_{HH}$ = 8.4 Hz, $^{4}J_{HF}$ = 5.0 Hz, $^{4}J_{HH}$ = 2.1 Hz, 1 H, H-10), 7.78 (dd, $^{4}J_{HF}$ = 7.6 Hz, $^{4}J_{HH}$ = 1.7 Hz, 1 H, H-14), 7.95 (d, $^{3}J_{HH}$ = 8.3 Hz, 2 H, H-3), 8.61 (s, 1 H, H-7); ^{13}C NMR (100 MHz, DMSO-d_6) δ (ppm) 14.1 (+, d, $^{3}J_{CF}$ = 3.0 Hz, 1 C, C-15), 52.5 (–, 1 C, C-6), 115.4 (+, d, $^{2}J_{CF}$ = 22.6 Hz, 1 C, C-11), 121.5 (+, 1 C, C-7), 124.5 (+, d,

$^{3}J_{CF}$ = 8.3 Hz, 1 C, C-10), 124.7 (C_q, d, $^{2}J_{CF}$ = 17.5 Hz, 1 C, C-13), 126.8 (C_q, d, $^{4}J_{CF}$ = 3.4 Hz, 1 C, C-9), 127.9 (+, 2 C, C-4), 128.4 (+, d, $^{3}J_{CF}$ = 5.1 Hz, 1 C, C-14), 129.7 (+, 2 C, C-3), 130.5 (C_q, 1 C, C-2), 140.6 (C_q, 1 C, C-5), 145.9 (C_q, 1 C, C-8), 160.3 (C_q, d, $^{1}J_{CF}$ = 243.5 Hz, 1 C, C-12), 166.9 (C_q, 1 C, C-1); IR (cm^{-1}) $\tilde{\nu}$: 3133 (w), 2673 (w), 1687 (s), 1426 (m), 1197 (m), 730 (s); HRMS (ESI+): [MH^+] calcd for $C_{17}H_{15}FN_3O_2$, 312.1143; found, 312.1146.

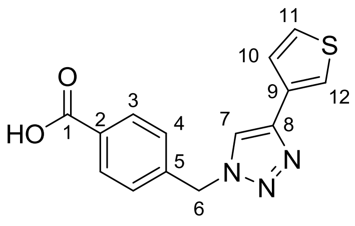

4-{[4-(Thiophen-3-yl)-1*H*-1,2,3-triazol-1-yl]methyl}benzoic acid (11r):

According to **GP 2**, the resin-bound molecule **11r** was synthesized, using 4-(azidomethyl)benzoic acid functionalized Wang resin (100 mg, 78 µmol, 1.0 equiv), L-ascorbic acid (6.9 mg, 39 µmol, 0.5 equiv), copper(II) sulfate pentahydrate (2.0 mg, 8 µmol, 0.1 equiv) and 3-ethynylthiophene (**10r**, 31 µL, 311 µmol, 4.0 equiv). Cleavage was done according to **GP 4**, followed by purification using a SPE tube. Subsequently, the solvent was evaporated, yielding compound **11r** (22 mg, 76 µmol, 98%) as a colorless solid.

Mp: 195 °C (decomposition); ^{1}H NMR (400 MHz, DMSO-d_6, HSQC) δ (ppm) 5.74 (s, 2 H, H-6), 7.41 (d, $^{3}J_{HH}$ = 8.4 Hz, 2 H, H-4), 7.52 (dd, $^{3}J_{HH}$ = 5.0 Hz, $^{4}J_{HH}$ = 1.2 Hz, 1 H, H-10), 7.63 (dd, $^{3}J_{HH}$ = 5.0 Hz, $^{4}J_{HH}$ = 3.0 Hz, 1 H, H-11), 7.86 (dd, $^{4}J_{HH}$ = 2.9 Hz, $^{4}J_{HH}$ = 1.2 Hz, 1 H, H-12), 7.95 (d, $^{3}J_{HH}$ = 8.4 Hz, 2 H, H-3), 8.51 (s, 1 H, H-7), 12.95 (s, 1 H, COOH); ^{13}C NMR (100 MHz, DMSO-d_6, HSQC) δ (ppm) 52.4 (–, 1 C, C-6), 120.9 (+, 1 C, C-12), 121.5 (+, 1 C, C-7), 125.7 (+, 1 C, C-10), 127.1 (+, 1 C, C-11), 127.8 (+, 2 C, C-4), 129.7 (+, 2 C, C-3), 130.5 (C_q, 1 C, C-2), 131.9 (C_q, 1 C, C-9), 140.7 (C_q, 1 C, C-5), 143.2 (C_q, 1 C, C-8), 166.9 (C_q, 1 C, C-1); IR (cm^{-1}) $\tilde{\nu}$: 3091 (w), 2920 (w), 2852 (w), 2668 (w), 2544 (w), 1681 (s), 1426 (m), 1283 (m), 779 (s), 730 (s); MS (TOF, ES+): m/z (%) = 571.1 (5) [$2MH^+$], 286.1 (100) [MH^+].

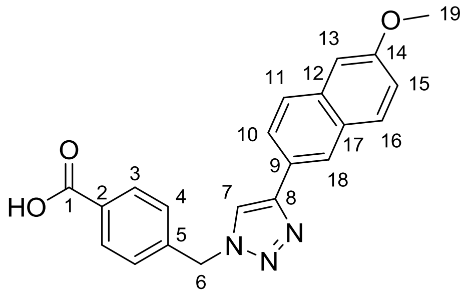

4-{[4-(6-Methoxynaphthalen-2-yl)-1*H*-1,2,3-triazol-1-yl]methyl}benzoic acid (11s):

According to **GP 2**, the resin-bound molecule **11s** was synthesized, using 4-(azidomethyl)benzoic acid functionalized Wang resin (100 mg, 78 µmol, 1.0 equiv), L-ascorbic acid (6.9 mg, 39 µmol, 0.5 equiv), copper(II) sulfate pentahydrate (2.0 mg, 8 µmol, 0.1 equiv) and 2-ethynyl-6-methoxynaphthalene (**10s**, 57 mg, 311 µmol, 4.0 equiv). Cleavage was done according to **GP 4**, followed by purification using a SPE tube. Subsequently, the solvent was evaporated, yielding compound **11s** (26 mg, 73 µmol, 94%) as a colorless solid.

Mp: 297 °C; ^{1}H NMR (400 MHz, DMSO-d_6, HSQC, HMBC) δ (ppm) 3.88 (s, 3 H, H-19), 5.77 (s, 2 H, H-6), 7.18 (dd, $^3J_{HH}$ = 8.9 Hz, $^4J_{HH}$ = 2.6 Hz, 1 H, H-15), 7.34 (d, $^4J_{HH}$ = 2.5 Hz, 1 H, H-13), 7.46 (d, $^3J_{HH}$ = 7.9 Hz, 2 H, H-4), 7.87 (d, $^3J_{HH}$ = 9.1 Hz, 1 H, H-16), 7.88 (d, $^3J_{HH}$ = 8.7 Hz, 1 H, H-11), 7.95 (dd, $^3J_{HH}$ = 8.6 Hz, $^4J_{HH}$ = 1.6 Hz, 1 H, H-10), 7.97 (d, $^3J_{HH}$ = 7.7 Hz, 2 H, H-3), 8.33 (d, $^4J_{HH}$ = 1.6 Hz, 1 H, H-18), 8.71 (s, 1 H, H-7), 13.03 (bs, 1 H, COOH); ^{13}C NMR (100 MHz, DMSO-d_6, HSQC, HMBC) δ (ppm) 52.5 (–, 1 C, C-6), 55.1 (+, 1 C, C-19), 105.9 (+, 1 C, C-13), 119.1 (+, 1 C, C-15), 121.6 (+, 1 C, C-7), 123.4 (+, 1 C, C-18), 124.0 (+, 1 C, C-10), 124.1 (C$_q$, 1 C, C-17), 125.7 (C$_q$, 1 C, C-9), 127.3 (+, 1 C, C-16), 127.9 (+, 2 C, C-4), 128.4 (C$_q$, 1 C, C-2), 129.4 (+, 1 C, C-11), 129.7 (+, 2 C, C-3), 133.8 (C$_q$, 1 C, C-12), 140.7 (C$_q$, 1 C, C-5), 146.9 (C$_q$, 1 C, C-8), 157.4 (C$_q$, 1 C, C-14), 170.4 (C$_q$, 1 C, C-1); IR (cm^{-1}) $\tilde{\nu}$: 3128 (w), 2920 (w), 2564 (w), 1690 (m), 1225 (m), 724 (s); HRMS (ESI+): [MH$^+$] calcd for $C_{21}H_{18}N_3O_3$, 360.1343; found, 360.1339.

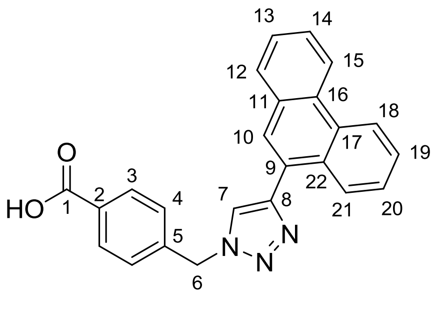

4-{[4-(Phenanthren-9-yl)-1*H*-1,2,3-triazol-1-yl]methyl}benzoic acid (11t):

According to **GP 2**, the resin-bound molecule **11t** was synthesized, using 4-(azidomethyl)benzoic acid functionalized Wang resin (100 mg, 78 µmol, 1.0 equiv), L-

ascorbic acid (6.9 mg, 39 µmol, 0.5 equiv), copper(II) sulfate pentahydrate (2.0 mg, 8 µmol, 0.1 equiv) and 9-ethynylphenanthrene (**10t**, 63 mg, 311 µmol, 4.0 equiv). Cleavage was done according to **GP 4**, followed by purification using a SPE tube. Subsequently, the solvent was evaporated, yielding compound **11t** (28 mg, 75 µmol, 96%) as a colorless solid.

Mp: 89–93 °C–^{1}H NMR (400 MHz, $CDCl_3$) δ (ppm) 5.74 (s, 2 H, H-6), 7.44 (d, $^3J_{HH}$ = 7.9 Hz, 2 H, H-4), 7.60 (t, $^3J_{HH}$ = 7.3 Hz, 2 H), 7.65–7.70 (m, 2 H), 7.89 (d, $^3J_{HH}$ = 7.2 Hz, 2 H), 7.98 (s, 1 H, H-7), 8.12 (d, $^3J_{HH}$ = 7.9 Hz, 2 H, H-3), 8.31 (d, $^3J_{HH}$ = 7.7 Hz, 1 H), 8.69 (d, $^3J_{HH}$ = 8.2 Hz, 1 H), 8.75 (d, $^3J_{HH}$ = 7.9 Hz, 1 H), 9.25 (bs, 1 H, COOH); ^{13}C NMR (100 MHz, $CDCl_3$) δ (ppm) 54.0 (–, 1 C, C-6), 122.5 (+, 1 C), 123.0 (+, 1 C), 125.9 (+, 1 C), 126.7 (+, 1 C), 126.9 (+, 1 C), 127.0 (+, 1 C), 127.2 (+, 1 C), 127.9 (+, 2 C, C-4), 128.5 (+, 1 C), 128.9 (+, 1 C), 129.9 (C_q, 1 C), 130.4 (C_q, 1 C), 130.7 (C_q, 1 C), 130.8 (+, 2 C, C-3), 131.2 (+, 1 C), 139.4 (C_q, 1 C), 177.6 (C_q, 1 C, C-1); IR (cm^{-1}) ṽ: 3129 (w), 2920 (w), 2854 (w), 1691 (m), 1427 (m), 1261 (m), 975 (m), 734 (s); MS (ESI+, TOF) m/z (%) 759.3 (50) [$2MH^+$], 380.1 (100) [MH^+].

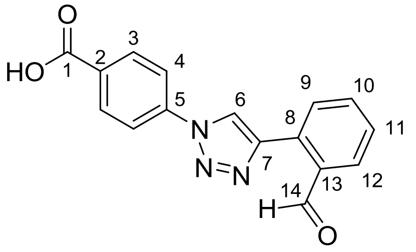

4-[4-(2-Formylphenyl)-1*H*-1,2,3-triazol-1-yl]benzoic acid (12a):

The synthesis of the resin-bound molecule **78** followed **GP 2**. The 4-azidobenzoic acid functionalized Wang resin **9** (100 mg, 79 µmol, 1.0 equiv) was treated with L-ascorbic acid (6.9 mg, 39 µmol, 0.5 equiv), copper(II) sulfate pentahydrate (2.0 mg, 8 µmol, 0.1 equiv) and 2-ethynylbenzaldehyde (**10f**, 41 mg, 314 µmol, 4.0 equiv). After the cleavage according to **GP 4**, the crude product was pre-purified using a SPE tube and then washed twice with 2 mL of a 1:1 mixture of dichloromethane/methanol, yielding compound **12a** (11 mg, 38 µmol, 49%) as a colorless solid.

^{1}H NMR (400 MHz, DMSO-d_6) δ (ppm) 7.64 (t, $^3J_{HH}$ = 7.5 Hz, 1 H, H-11), 7.83 (td, $^3J_{HH}$ = 7.5 Hz, $^4J_{HH}$ = 1.4 Hz, 1 H, H-10), 7.89 (dd, $^3J_{HH}$ = 7.7 Hz, $^4J_{HH}$ = 1.0 Hz, 1 H, H-12), 7.96 (dd, $^3J_{HH}$ = 7.8 Hz, $^4J_{HH}$ = 1.0 Hz, 1 H, H-9), 8.17 (d, $^3J_{HH}$ = 8.9 Hz, 2 H, H-4), 8.20 (d, $^3J_{HH}$ = 8.9 Hz, 2 H, H-3), 9.48 (s, 1 H, H-6), 10.43 (bs, 1 H, H-14), 13.25 (bs, 1 H, COOH); ^{13}C NMR (100 MHz, DMSO-d_6) δ (ppm) 119.9 (+, 2 C, C-4), 123.0 (+, 1 C, C-6), 127.6 (+, 1 C, C-9), 128.9 (+, 1 C, C-12), 129.8 (+, 1 C, C-11), 131.1 (+, 2 C, C-3), 132.3 (C_q, 1 C, C-2), 133.5 (C_q, 1 C, C-8), 134.0 (+, 1 C, C-10), 137.3 (C_q, 1 C, C-13), 139.3 (C_q, 1 C, C-5),

145.0 (C_q, 1 C, C-7), 167.0 (C_q, 1 C, C-1), 192.3 (+, 1 C, C-14); MS (ESI+, TOF) m/z (%) 294.1 (100) [MH^+].

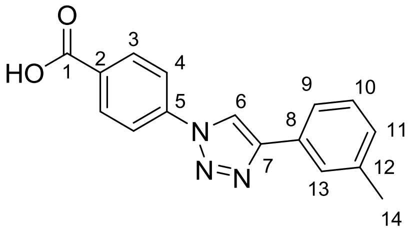

4-(4-m-Tolyl-1*H*-1,2,3-triazol-1-yl)benzoic acid (12b):

The synthesis of the resin-bound molecule **12b** followed **GP 2**. The 4-azidobenzoic acid functionalized Wang resin **9** (100 mg, 79 µmol, 1.0 equiv) was treated with L-ascorbic acid (6.9 mg, 39 µmol, 0.5 equiv), copper(II) sulfate pentahydrate (2.0 mg, 8 µmol, 0.1 equiv) and 3-ethynyltoluene (**10g**, 41 µL, 314 µmol, 4.0 equiv). After the cleavage according to **GP 4**, the crude product was pre-purified using a SPE tube and then washed twice with 2 mL of a 1:1 mixture of dichloromethane/methanol, yielding compound **12b** (10 mg, 35 µmol, 44%) as a colorless solid.

^{1}H NMR (400 MHz, DMSO-d_6) δ (ppm) 2.40 (s, 3 H, H-14), 7.22 (d, $^3J_{HH}$ = 7.9 Hz, 1 H, H-11), 7.40 (t, $^3J_{HH}$ = 7.7 Hz, 1 H, H-10), 7.76 (d, $^3J_{HH}$ = 7.7 Hz, 2 H, H-9), 7.80 (s, 1 H, H-13), 8.09 (d, $^3J_{HH}$ = 8.7 Hz, 2 H, H-4), 8.17 (d, $^3J_{HH}$ = 8.7 Hz, 2 H, H-3), 9.41 (s, 1 H, H-6), 13.09 (bs, 1 H, COOH); IR (cm^{-1}) $\tilde{\nu}$: 2682 (w), 2556 (w), 2114 (w), 1702 (s), 1604 (s), 1518 (m), 1379 (m), 1320 (m), 1294 (m), 1036 (s), 782 (s); HRMS (ESI+): [MH^+] calcd for $C_{16}H_{14}N_3O_2$, 280.1081; found, 280.1084.

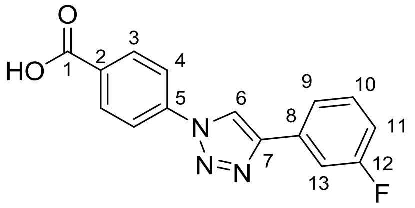

4-[4-(3-Fluorophenyl)-1*H*-1,2,3-triazol-1-yl]benzoic acid (12c):

The synthesis of the resin-bound molecule **12c** followed **GP 2**. The 4-azidobenzoic acid functionalized Wang resin **9** (100 mg, 79 µmol, 1.0 equiv) was treated with L-ascorbic acid (6.9 mg, 39 µmol, 0.5 equiv), copper(II) sulfate pentahydrate (2.0 mg, 8 µmol, 0.1 equiv) and 1-ethynyl-3-fluorobenzene (**10h**, 36 µL, 314 µmol, 4.0 equiv). After the cleavage according to **GP 4**, the crude product was pre-purified using a SPE tube and then washed twice with 2 mL of a 1:1 mixture of dichloromethane/methanol, yielding compound **12c** (10 mg, 35 µmol, 45%) as a colorless solid.

^{1}H NMR (400 MHz, DMSO-d_6) δ (ppm) 7.25 (dddd, $^{3}J_{HF}$ = 8.6 Hz, $^{3}J_{HH}$ = 8.6 Hz, $^{4}J_{HH}$ = 2.6 Hz, $^{4}J_{HH}$ = 0.7 Hz, 1 H, H-11), 7.58 (ddd, $^{3}J_{HH}$ = 8.0 Hz, $^{3}J_{HH}$ = 7.9 Hz, $^{4}J_{HF}$ = 6.2 Hz, 1 H, H-10), 7.76 (ddd, $^{3}J_{HF}$ = 10.2 Hz, $^{4}J_{HH}$ = 2.5 Hz, $^{4}J_{HH}$ = 1.5 Hz, 1 H, H-13), 7.82 (ddd, $^{3}J_{HH}$ = 7.8 Hz, $^{4}J_{HH}$ = 1.2 Hz, $^{4}J_{HH}$ = 1.0 Hz, 1 H, H-9), 8.10 (d, $^{3}J_{HH}$ = 8.8 Hz, 2 H, H-4), 8.19 (d, $^{3}J_{HH}$ = 8.8 Hz, 2 H, H-3), 9.52 (s, 1 H, H-6), 13.25 (bs, 1 H, COOH); ^{13}C NMR (100 MHz, DMSO-d_6) δ (ppm) 111.9 (+, d, $^{2}J_{CF}$ = 22.9 Hz, 1 C, C-11), 115.0 (+, d, $^{2}J_{CF}$ = 20.9 Hz, 1 C, C-13), 119.7 (+, 2 C, C-4), 120.4 (+, 1 C, C-6), 121.3 (+, d, $^{4}J_{CF}$ = 2.8 Hz, 1 C, C-9), 130.7 (C_q, 1 C, C-2), 131.1 (+, 2 C, C-3), 131.2 (+, d, $^{3}J_{CF}$ = 8.8 Hz, 1 C, C-10), 132.3 (C_q, d, $^{3}J_{CF}$ = 8.5 Hz, 1 C, C-8), 139.5 (C_q, 1 C, C-5), 146.4 (C_q, d, $^{4}J_{CF}$ = 3.1 Hz, 1 C, C-7), 162.5 (C_q, d, $^{1}J_{CF}$ = 243.1 Hz, 1 C, C-12), 166.3 (C_q, 1 C, C-1); IR (cm^{-1}) ṽ: 2918 (w), 2849 (w), 2559 (w), 2112 (w), 1682 (s), 1606 (s), 1293 (m), 1229 (m), 1034 (m), 860 (s), 769 (s); HRMS (ESI+): [MH^{+}] calcd for $C_{15}H_{11}FN_3O_2$, 284.0830; found, 284.0841.

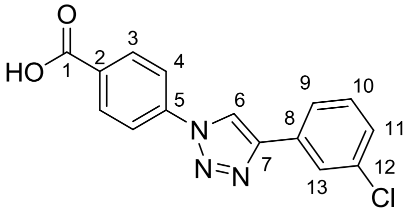

4-[4-(3-Chlorophenyl)-1*H*-1,2,3-triazol-1-yl]benzoic acid (12d):

The synthesis of the resin-bound molecule **12d** followed **GP 2**. The 4-azidobenzoic acid functionalized Wang resin **9** (100 mg, 79 µmol, 1.0 equiv) was treated with L-ascorbic acid (6.9 mg, 39 µmol, 0.5 equiv), copper(II) sulfate pentahydrate (2.0 mg, 8 µmol, 0.1 equiv) and 3-chloro-1-ethynylbenzene (**10i**, 38 µL, 314 µmol, 4.0 equiv). After the cleavage according to **GP 4**, the crude product was pre-purified using a SPE tube and then washed twice with 2 mL of a 1:1 mixture of dichloromethane/methanol, yielding compound **12d** (10 mg, 35 µmol, 44%) as a colorless solid.

^{1}H NMR (400 MHz, DMSO-d_6) δ (ppm) 7.47 (ddd, $^{3}J_{HH}$ = 8.0 Hz, $^{4}J_{HH}$ = 2.1 Hz, $^{4}J_{HH}$ = 1.0 Hz, 1 H, H-11), 7.56 (t, $^{3}J_{HH}$ = 7.9 Hz, 1 H, H-10), 7.95 (dt, $^{3}J_{HH}$ = 7.8 Hz, $^{4}J_{HH}$ = 1.2 Hz, 1 H, H-9), 8.01 (t, $^{4}J_{HH}$ = 1.8 Hz, 1 H, H-13), 8.10 (d, $^{3}J_{HH}$ = 8.7 Hz, 2 H, H-4), 8.19 (d, $^{3}J_{HH}$ = 8.7 Hz, 2 H, H-3), 9.55 (s, 1 H, H-6), 13.23 (bs, 1 H, COOH); ^{13}C NMR (100 MHz, DMSO-d_6) δ (ppm) 119.6 (+, 2 C, C-4), 120.5 (+, 1 C, C-6), 123.8 (+, 1 C, C-9), 124.9 (+, 1 C, C-13), 128.1 (+, 1 C, C-11), 131.0 (+, 1 C, C-10), 131.1 (+, 2 C, C-3), 132.0 (C_q, 1 C, C-8), 133.7 (C_q, 1 C, C-12), 139.3 (C_q, 1 C, C-5), 146.1 (C_q, 1 C, C-7); IR (cm^{-1}) ṽ: 2818 (w), 2549 (w), 2109 (w), 1681 (s), 1604 (s), 1519 (m), 1290 (s), 1226 (s), 1180 (m), 1031 (s), 940 (m), 767 (s); HRMS (ESI+): [MH^{+}] calcd for $C_{15}H_{11}ClN_3O_2$, 300.0534; found, 300.0565.

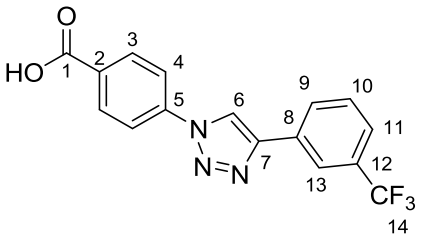

4-{4-[3-(Trifluoromethyl)phenyl]-1*H*-1,2,3-triazol-1-yl}benzoic acid (12e):

Literature known compound; improved procedure [2]

The synthesis of the resin-bound molecule **12e** followed **GP 2**. The 4-azidobenzoic acid functionalized Wang resin **9** (100 mg, 79 µmol, 1.0 equiv) was treated with L-ascorbic acid (6.9 mg, 39 µmol, 0.5 equiv), copper(II) sulfate pentahydrate (2.0 mg, 8 µmol, 0.1 equiv) and 3-ethynyl-α,α,α-trifluorotoluene (**10j**, 45 µL, 314 µmol, 4.0 equiv). After the cleavage according to **GP 4**, the crude product was pre-purified using a SPE tube and then washed twice with 2 mL of a 1:1 mixture of dichloromethane/methanol, yielding compound **12e** (11 mg, 32 µmol, 41%) as a colorless solid.

^{1}H NMR (400 MHz, DMSO-d_6) δ (ppm) 7.76–7.80 (m, 2 H, H-10, H-11), 8.11 (d, $^3J_{HH}$ = 8.6 Hz, 2 H, H-4), 8.20 (d, $^3J_{HH}$ = 8.7 Hz, 2 H, H-3), 8.26–8.32 (m, 2 H, H-9, H-13), 9.65 (s, 1 H, H-6), 13.09 (bs, 1 H, COOH); HRMS (ESI+): [MH$^+$] calcd for $C_{16}H_{11}F_3N_3O_2$, 334.0798; found, 334.0802.

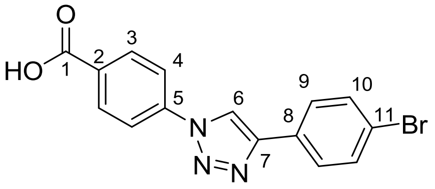

4-[4-(4-Bromophenyl)-1*H*-1,2,3-triazol-1-yl]benzoic acid (12f):

The synthesis of the resin-bound molecule **12f** followed **GP 2**. The 4-azidobenzoic acid functionalized Wang resin **9** (100 mg, 79 µmol, 1.0 equiv) was treated with L-ascorbic acid (6.9 mg, 39 µmol, 0.5 equiv), copper(II) sulfate pentahydrate (2.0 mg, 8 µmol, 0.1 equiv) and 1-bromo-4-ethynylbenzene (**10k**, 57 mg, 314 µmol, 4.0 equiv). After the cleavage according to **GP 4**, the crude product was pre-purified using a SPE tube before the purification was done by column chromatography on silica gel (ethyl acetate/petroleum ether 4:1, R_f 0.49), yielding compound **12f** (6 mg, 16 µmol, 21%) as a colorless solid.

^{1}H NMR (400 MHz, DMSO-d_6) δ (ppm) 7.71 (d, $^3J_{HH}$ = 8.5 Hz, 2 H, H-10), 7.82 (d, $^3J_{HH}$ = 8.6 Hz, 2 H, H-9), 8.01 (d, $^3J_{HH}$ = 8.8 Hz, 2 H, H-4), 8.12 (d, $^3J_{HH}$ = 8.8 Hz, 2 H, H-3), 9.34 (s, 1 H, H-6); MS (ESI+, TOF) m/z (%) 344.1, 346.0 (50) [MH$^+$].

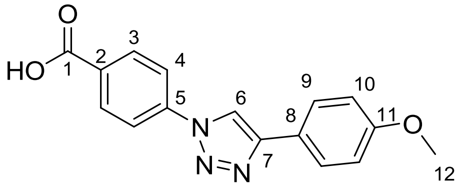

4-[4-(4-Methoxyphenyl)-1*H*-1,2,3-triazol-1-yl]benzoic acid (12g):

Literature known compound; improved procedure [3].

The synthesis of the resin-bound molecule **12g** followed **GP 2**. The 4-azidobenzoic acid functionalized Wang resin **9** (100 mg, 79 µmol, 1.0 equiv) was treated with L-ascorbic acid (6.9 mg, 39 µmol, 0.5 equiv), copper(II) sulfate pentahydrate (2.0 mg, 8 µmol, 0.1 equiv) and 4-ethynylanisole (**10l**, 41 µL, 314 µmol, 4.0 equiv). After the cleavage according to **GP 4**, the crude product was pre-purified using a SPE tube and then washed twice with 2 mL of a 1:1 mixture of dichloromethane/methanol, yielding compound **12g** (12 mg, 41 µmol, 52%) as a colorless solid.

^{1}H NMR (400 MHz, DMSO-d_6) δ (ppm) 3.82 (s, 3 H, H-12), 7.08 (d, $^3J_{HH}$ = 8.9 Hz, 2 H, H-10), 7.89 (d, $^3J_{HH}$ = 8.8 Hz, 2 H, H-9), 8.10 (d, $^3J_{HH}$ = 8.8 Hz, 2 H, H-4), 8.18 (d, $^3J_{HH}$ = 8.8 Hz, 2 H, H-3), 9.32 (s, 1 H, H-6), 13.25 (bs, 1 H, COOH); ^{13}C NMR (100 MHz, DMSO-d_6) δ (ppm) 55.1 (+, 1 C, C-12), 114.4 (+, 2 C, C-10), 118.6 (+, 1 C, C-6), 119.5 (+, 2 C, C-4), 122.4 (C_q, 1 C, C-8), 126.7 (+, 2 C, C-9), 130.5 (C_q, 1 C, C-2), 131.0 (+, 2 C, C-3), 139.5 (C_q, 1 C, C-5), 147.5 (C_q, 1 C, C-7), 159.3 (C_q, 1 C, C-11), 166.3 (C_q, 1 C, C-1); IR (cm^{-1}) $\tilde{\nu}$: 3115 (w), 2912 (w), 2777 (w), 1682 (m), 1409 (m), 1284 (m), 1226 (s), 1028 (m), 947 (m), 837 (s), 771 (s); HRMS (ESI+): [MH$^+$] calcd for $C_{16}H_{14}N_3O_3$, 296.1030; found, 296.1049.

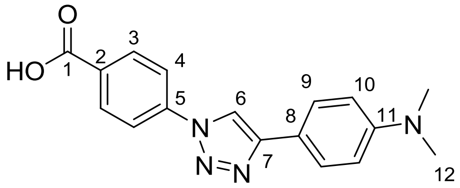

4-{4-[4-(Dimethylamino)phenyl]-1*H*-1,2,3-triazol-1-yl}benzoic acid (12h):

The synthesis of the resin-bound molecule **12h** followed **GP 2**. The 4-azidobenzoic acid functionalized Wang resin **9** (100 mg, 79 µmol, 1.0 equiv) was treated with L-ascorbic acid (6.9 mg, 39 µmol, 0.5 equiv), copper(II) sulfate pentahydrate (2.0 mg, 8 µmol, 0.1 equiv) and 4-ethynyl-*N*,*N*-dimethylaniline (**10m**, 46 mg, 314 µmol, 4.0 equiv). After the cleavage according to **GP 4**, the crude product was pre-purified using a SPE tube and then washed twice with 2 mL of a 1:1 mixture of DCM / methanol, yielding compound **12h** (9 mg, 30 µmol, 38%) as a colorless solid.

^{1}H NMR (400 MHz, DMSO-d_6) δ (ppm) 2.96 (s, 6 H, H-12), 6.84 (d, $^3J_{HH}$ = 9.0 Hz, 2 H, H-9), 7.77 (d, $^3J_{HH}$ = 8.9 Hz, 2 H, H-10), 8.10 (d, $^3J_{HH}$ = 8.8 Hz, 2 H, H-4), 8.17 (d, $^3J_{HH}$ = 8.8 Hz, 2 H, H-3), 9.22 (s, 1 H, H-6), 13.02 (bs, 1 H, COOH); HRMS (ESI+): [MH$^+$] calcd for $C_{17}H_{17}N_4O_2$, 309.1346; found, 309.1308.

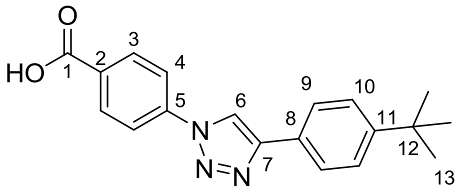

4-[4-(4-*tert*-Butylphenyl)-1*H*-1,2,3-triazol-1-yl]benzoic acid (12i):

The synthesis of the resin-bound molecule **12i** followed **GP 2**. The 4-azidobenzoic acid functionalized Wang resin **9** (100 mg, 79 µmol, 1.0 equiv) was treated with L-ascorbic acid (6.9 mg, 39 µmol, 0.5 equiv), copper(II) sulfate pentahydrate (2.0 mg, 8 µmol, 0.1 equiv) and 4-*tert*-butylphenylacetylene (**10n**, 57 µL, 314 µmol, 4.0 equiv). After the cleavage according to **GP 4**, the crude product was pre-purified using a SPE tube and then washed twice with 2 mL of a 1:1 mixture of dichloromethane/methanol, yielding compound **12i** (10 mg, 32 µmol, 41%) as a colorless solid.

^{1}H NMR (400 MHz, DMSO-d_6) δ (ppm) 1.33 (s, 9 H, H-13), 7.54 (d, $^3J_{HH}$ = 8.4 Hz, 2 H, H-10), 7.88 (d, $^3J_{HH}$ = 8.4 Hz, 2 H, H-9), 8.12 (d, $^3J_{HH}$ = 8.7 Hz, 2 H, H-4), 8.18 (d, $^3J_{HH}$ = 8.7 Hz, 2 H, H-3), 9.40 (s, 1 H, H-6), 13.04 (bs, 1 H, COOH); IR (cm^{-1}) ṽ: 2554 (w), 2108 (w), 1677 (s), 1601 (s), 1425 (m), 1284 (s), 1252 (s), 1176 (m), 1024 (m), 766 (s); MS (ESI+, TOF) m/z (%) 643.3 (5) [2MH$^+$], 322.2 (100) [MH$^+$].

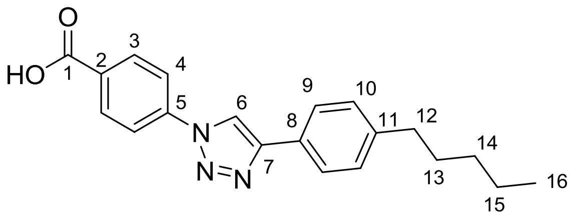

4-[4-(4-Pentylphenyl)-1*H*-1,2,3-triazol-1-yl]benzoic acid (12j):

The synthesis of the resin-bound molecule **12j** followed **GP 2**. The 4-azidobenzoic acid functionalized Wang resin **9** (100 mg, 79 µmol, 1.0 equiv) was treated with L-ascorbic acid (6.9 mg, 39 µmol, 0.5 equiv), copper(II) sulfate pentahydrate (2.0 mg, 8 µmol, 0.1 equiv) and 1-ethynyl-4-pentylbenzene (**10o**, 61 µL, 314 µmol, 4.0 equiv). After the cleavage according to **GP 4**, the crude product was pre-purified using a SPE tube and then washed twice with 2 mL

of a 1:1 mixture of dichloromethane/methanol, yielding compound **12j** (17 mg, 49 µmol, 63%) as a colorless solid.

^{1}H NMR (400 MHz, DMSO-d_6, HSQC) δ (ppm) 0.87 (t, $^3J_{HH}$ = 6.9 Hz, 3 H, H-16), 1.25–1.38 (m, 4 H, H-14, H-15), 1.61 (quintet, $^3J_{HH}$ = 7.5 Hz, 2 H, H-13), 2.62 (t, $^3J_{HH}$ = 7.6 Hz, 2 H, H-12), 7.33 (d, $^3J_{HH}$ = 8.1 Hz, 2 H, H-10), 7.86 (d, $^3J_{HH}$ = 8.1 Hz, 2 H, H-9), 8.11 (d, $^3J_{HH}$ = 8.8 Hz, 2 H, H-4), 8.18 (d, $^3J_{HH}$ = 8.7 Hz, 2 H, H-3), 9.38 (s, 1 H, H-6), 13.05 (bs, 1 H, COOH); ^{13}C NMR (100 MHz, DMSO-d_6, HSQC) δ (ppm) 13.8 (+, 1 C, C-16), 21.9 (–, 1 C, C-15), 30.4 (–, 1 C, C-14), 30.8 (–, 1 C, C-13), 34.8 (–, 1 C, C-12), 119.1 (+, 1 C, C-6), 119.5 (+, 2 C, C-4), 125.3 (+, 2 C, C-9), 127.9 (C_q, 1 C, C-8), 128.8 (+, 2 C, C-10), 130.5 (C_q, 1 C, C-2), 131.0 (+, 2 C, C-3), 139.5 (C_q, 1 C, C-5), 142.7 (C_q, 1 C, C-11), 147.6 (C_q, 1 C, C-7), 166.3 (C_q, 1 C, C-1); IR (cm^{-1}) ṽ: 2957 (w), 2924 (w), 2854 (w), 2108 (w), 1681 (s), 1604 (s), 1428 (m), 1288 (s), 1230 (s), 1037 (m), 860 (m), 769 (s); HRMS (ESI+): [MH$^+$] calcd for $C_{20}H_{22}N_3O_2$, 336.1707; found, 336.1706.

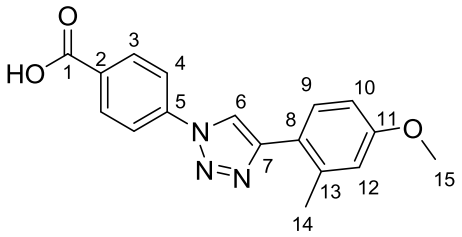

4-[4-(4-Methoxy-2-methylphenyl)-1*H*-1,2,3-triazol-1-yl]benzoic acid (12k):

The synthesis of the resin-bound molecule **12k** followed **GP 2**. The 4-azidobenzoic acid functionalized Wang resin **9** (100 mg, 79 µmol, 1.0 equiv) was treated with L-ascorbic acid (6.9 mg, 39 µmol, 0.5 equiv), copper(II) sulfate pentahydrate (2.0 mg, 8 µmol, 0.1 equiv) and 1-ethynyl-4-methoxy-2-methylbenzene (**10p**, 46 mg, 314 µmol, 4.0 equiv). After the cleavage according to **GP 4**, the crude product was pre-purified using a SPE tube before the purification was done by column chromatography on silica gel (ethyl acetate/petroleum ether 4:1, R_f 0.31), yielding compound **12k** (11 mg, 37 µmol, 47%) as a colorless solid.

^{1}H NMR (400 MHz, DMSO-d_6, HSQC, HMBC) δ (ppm) 2.49 (s, 3 H, H-14), 3.80 (s, 3 H, H-15), 6.90 (dd, $^3J_{HH}$ = 8.5 Hz, $^4J_{HH}$ = 2.5 Hz, 1 H, H-10), 6.93 (d, $^4J_{HH}$ = 2.5 Hz, 1 H, H-12), 7.72 (d, $^3J_{HH}$ = 8.4 Hz, 1 H, H-9), 7.99 (d, $^3J_{HH}$ = 8.4 Hz, 2 H, H-4), 8.15 (d, $^3J_{HH}$ = 8.4 Hz, 2 H, H-3), 8.98 (s, 1 H, H-6); ^{13}C NMR (100 MHz, DMSO-d_6, HSQC, HMBC) δ (ppm) 21.2 (+, 1 C, C-14), 55.0 (+, 1 C, C-15), 111.6 (+, 1 C, C-10), 116.0 (+, 1 C, C-12), 119.0 (+, 2 C, C-4), 120.4 (+, 1 C, C-6), 122.0 (C_q, 1 C, C-8), 129.8 (+, 1 C, C-9), 131.0 (+, 2 C, C-3), 136.9 (C_q, 1 C, C-13), 137.7 (C_q, 1 C, C-5), 146.7 (C_q, 1 C, C-7), 159.0 (C_q, 1 C, C-11), 167.4 (C_q, 1 C, C-1); HRMS (ESI+): [MH$^+$] calcd for $C_{17}H_{16}N_3O_3$, 310.1179; found, 310.1186.

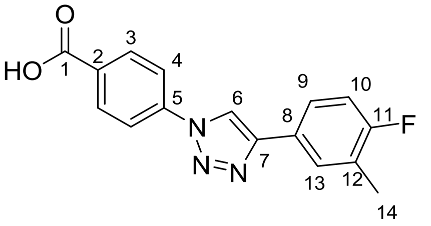

4-[4-(4-Fluoro-3-methylphenyl)-1*H*-1,2,3-triazol-1-yl]benzoic acid (12l):

The synthesis of the resin-bound molecule **12l** followed **GP 2**. The 4-azidobenzoic acid functionalized Wang resin **9** (100 mg, 79 µmol, 1.0 equiv) was treated with L-ascorbic acid (6.9 mg, 39 µmol, 0.5 equiv), copper(II) sulfate pentahydrate (2.0 mg, 8 µmol, 0.1 equiv) and 4-ethynyl-1-fluoro-2-methylbenzene (**10q**, 42 µL, 314 µmol, 4.0 equiv). After the cleavage according to **GP 4**, the crude product was pre-purified using a SPE tube and then washed twice with 2 mL of a 1:1 mixture of dichloromethane/methanol, yielding compound **12l** (9 mg, 31 µmol, 40%) as a colorless solid.

^{1}H NMR (400 MHz, DMSO-d_6) δ (ppm) 2.33 (d, $^4J_{HF}$ = 1.5 Hz, 3 H, H-14), 7.23 (dd, $^3J_{HF}$ = 9.8 Hz, $^3J_{HH}$ = 8.4 Hz, 1 H, H-10), 7.80 (ddd, $^3J_{HH}$ = 7.8 Hz, $^4J_{HF}$ = 4.8 Hz, $^4J_{HH}$ = 2.1 Hz, 1 H, H-9), 7.90 (dd, $^4J_{HF}$ = 7.5 Hz, $^4J_{HH}$ = 1.7 Hz, 1 H, H-13), 8.10 (d, $^3J_{HH}$ = 8.6 Hz, 2 H, H-4), 8.18 (d, $^3J_{HH}$ = 8.6 Hz, 2 H, H-3), 9.41 (s, 1 H, H-6), 13.15 (bs, 1 H, COOH); MS (ESI+, TOF) m/z (%) 298.1 (100) [MH$^+$].

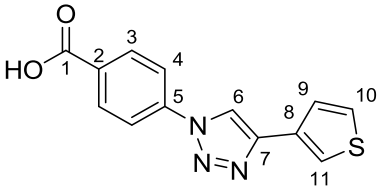

4-[4-(Thiophen-3-yl)-1*H*-1,2,3-triazol-1-yl]benzoic acid (12m):

Literature known compound, improved procedure [4]

The synthesis of the resin-bound molecule **12m** followed **GP 2**. The 4-azidobenzoic acid functionalized Wang resin **9** (100 mg, 79 µmol, 1.0 equiv) was treated with L-ascorbic acid (6.9 mg, 39 µmol, 0.5 equiv), copper(II) sulfate pentahydrate (2.0 mg, 8 µmol, 0.1 equiv) and 3-ethynylthiophene (**10r**, 31 µL, 314 µmol, 4.0 equiv). After the cleavage according to **GP 4**, the crude product was pre-purified using a SPE tube and then washed twice with 2 mL of a 1:1 mixture of dichloromethane/methanol, yielding compound **12m** (13 mg, 48 µmol, 61%) as a colorless solid.

^{1}H NMR (400 MHz, DMSO-d_6) δ (ppm) 7.60 (dd, $^3J_{HH}$ = 5.0 Hz, $^4J_{HH}$ = 1.2 Hz, 1 H, H-9), 7.73 (dd, $^3J_{HH}$ = 5.0 Hz, $^4J_{HH}$ = 3.0 Hz, 1 H, H-10), 7.97 (dd, $^4J_{HH}$ = 2.9 Hz, $^4J_{HH}$ = 1.2 Hz, 1 H, H-

11), 8.09 (d, $^{3}J_{HH}$ = 8.8 Hz, 2 H, H-4), 8.18 (d, $^{3}J_{HH}$ = 8.8 Hz, 2 H, H-3), 9.31 (s, 1 H, H-6), 13.20 (bs, 1 H, COOH); MS (ESI+, TOF) m/z (%) 272.1 (100) [MH^{+}].

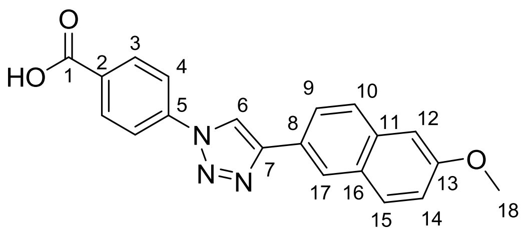

4-[4-(6-Methoxynaphthalen-2-yl)-1*H*-1,2,3-triazol-1-yl]benzoic acid (12n):

The synthesis of the resin-bound molecule **12n** followed **GP 2**. The 4-azidobenzoic acid functionalized Wang resin **9** (100 mg, 79 µmol, 1.0 equiv) was treated with L-ascorbic acid (6.9 mg, 39 µmol, 0.5 equiv), copper(II) sulfate pentahydrate (2.0 mg, 8 µmol, 0.1 equiv) and 2-ethynyl-6-methoxynaphthalene (**10s**, 57 mg, 314 µmol, 4.0 equiv). After the cleavage according to **GP 4**, the crude product was pre-purified using a SPE tube and then washed twice with 2 mL of a 1:1 mixture of dichloromethane/methanol, yielding compound **12n** (7 mg, 20 µmol, 26%) as a red solid.

^{1}H NMR (400 MHz, DMSO-d_6, HSQC, HMBC) δ (ppm) 3.91 (s, 3 H, H-18), 7.23 (dd, $^{3}J_{HH}$ = 8.9 Hz, $^{4}J_{HH}$ = 2.6 Hz, 1 H, H-14), 7.39 (d, $^{4}J_{HH}$ = 2.4 Hz, 1 H, H-12), 7.58 (d, $^{3}J_{HH}$ = 9.1 Hz, 1 H, H-15), 8.04 (d, $^{3}J_{HH}$ = 8.5 Hz, $^{4}J_{HH}$ = 1.7 Hz, 1 H, H-9), 8.10 (d, $^{3}J_{HH}$ = 8.8 Hz, 1 H, H-10), 8.14 (d, $^{3}J_{HH}$ = 8.8 Hz, 2 H, H-4), 8.20 (d, $^{3}J_{HH}$ = 8.8 Hz, 2 H, H-3), 8.40 (d, $^{4}J_{HH}$ = 1.6 Hz, 1 H, H-17), 9.51 (s, 1 H, H-6), 13.20 (bs, 1 H, COOH); IR (cm^{-1}) $\tilde{\nu}$: 2838 (w), 2525 (bw), 2110 (m), 1682 (m), 1603 (s), 1510 (m), 1266 (s), 1175 (m), 1026 (m), 981 (s), 856 (m), 786 (s); MS (ESI+, TOF) m/z (%) 346.1 (100) [MH^{+}].

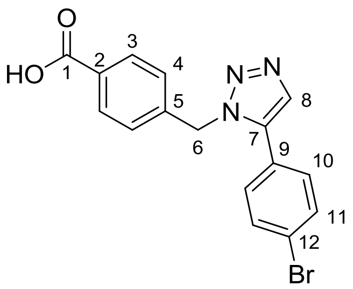

4-{[5-(4-Bromophenyl)-1*H*-1,2,3-triazol-1-yl]methyl}benzoic acid (13f):

Compound **13f** was synthesized according to **GP 3**, using 4-(azidomethyl)-benzoic acid functionalized Wang resin **7** (100 mg, 78 µmol, 1.0 equiv), Cp × RuCl(PPh_3)$_2$ (3 mg, 4 µmol, 0.05 equiv) and 1-bromo-4-ethynylbenzene (**10k**, 56 mg, 311 µmol, 4.0 equiv), followed by **GP 4**. The cleaved crude product, was pre-purified using a SPE tube before the purification

was done by preparative TLC (ethyl acetate/petroleum ether 4:1, R_f 0.72) yielding compound **13f** (13 mg, 38 µmol, 48%) as a colorless solid.

^{1}H NMR (400 MHz, DMSO-d_6) δ (ppm) 5.72 (s, 2 H, H-6), 6.98 (d, $^3J_{HH}$ = 8.2 Hz, 2 H, H-4), 7.41 (d, $^3J_{HH}$ = 8.5 Hz, 2 H, H-11), 7.67 (d, $^3J_{HH}$ = 8.5 Hz, 2 H, H-10), 7.84 (d, $^3J_{HH}$ = 8.2 Hz, 2 H, H-3), 8.00 (s, 1 H, H-8); HRMS (ESI+) (m/z): [$MH^{+\cdot}$]: calcd for $C_{16}H_{13}BrN_3O_2$, 358.0186; found, 358.0197; MF: $C_{16}H_{12}BrN_3O_2$; MW: 358.19

Copies of spectra of new compounds

^{1}H-NMR (300 MHz, $CDCl_3$)

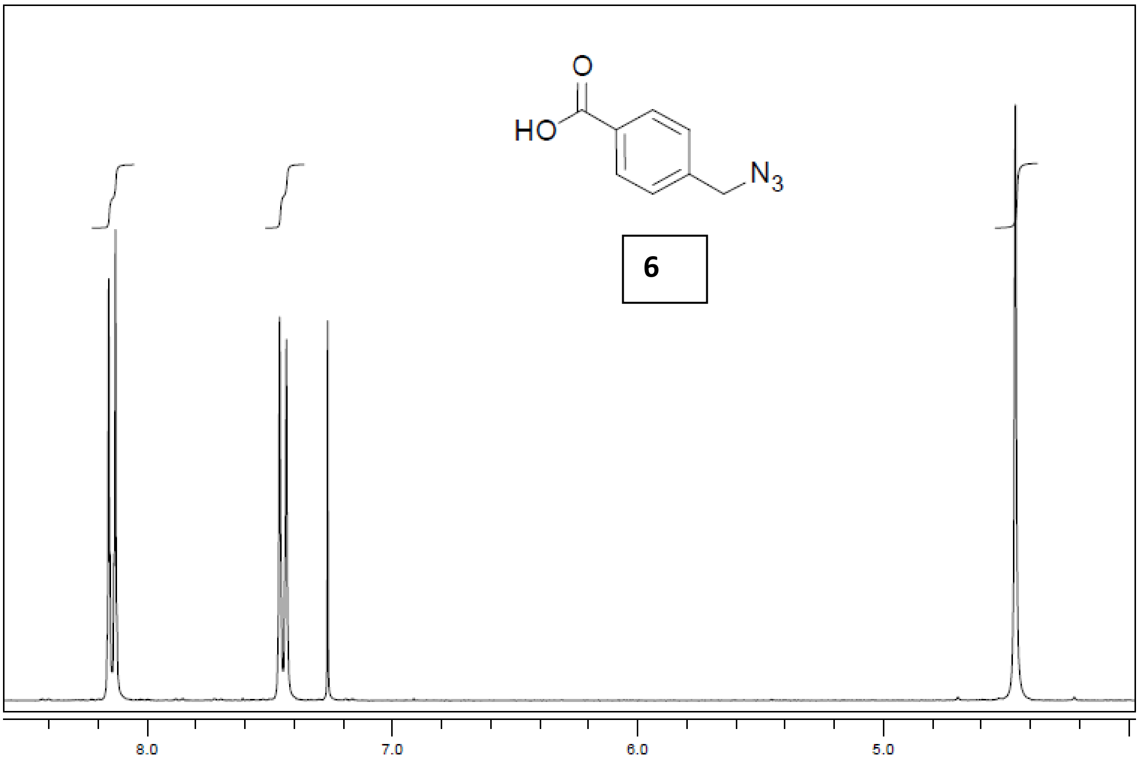

^{13}C-NMR (75 MHz, $CDCl_3$)

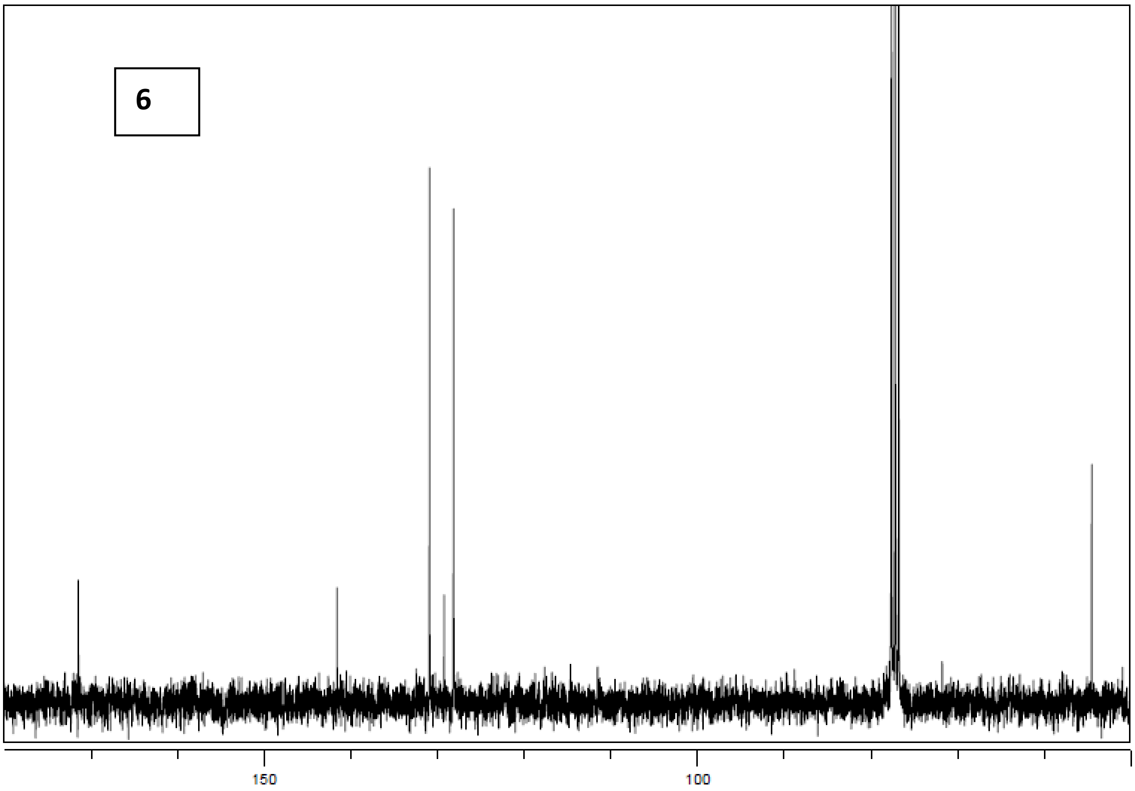

^{1}H-NMR (400 MHz, DMSO-d_6)

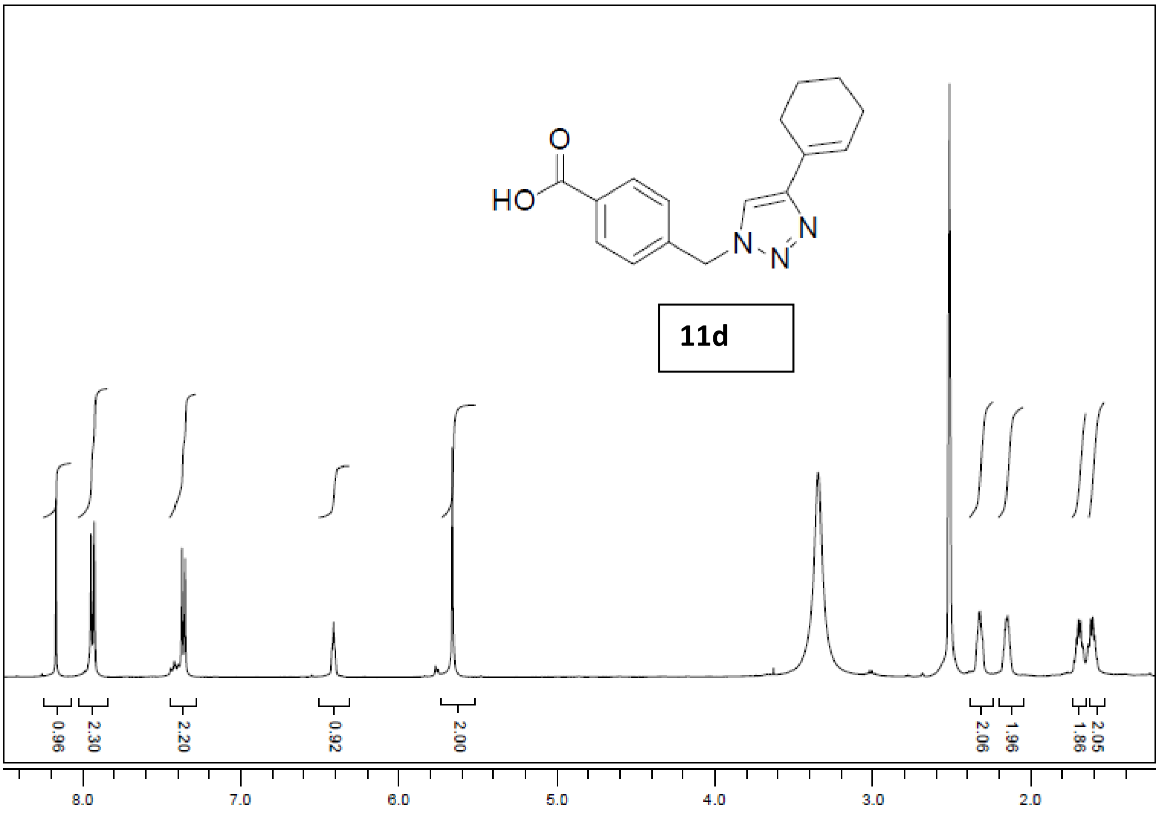

^{13}C-NMR (100 MHz, DMSO-d_6)

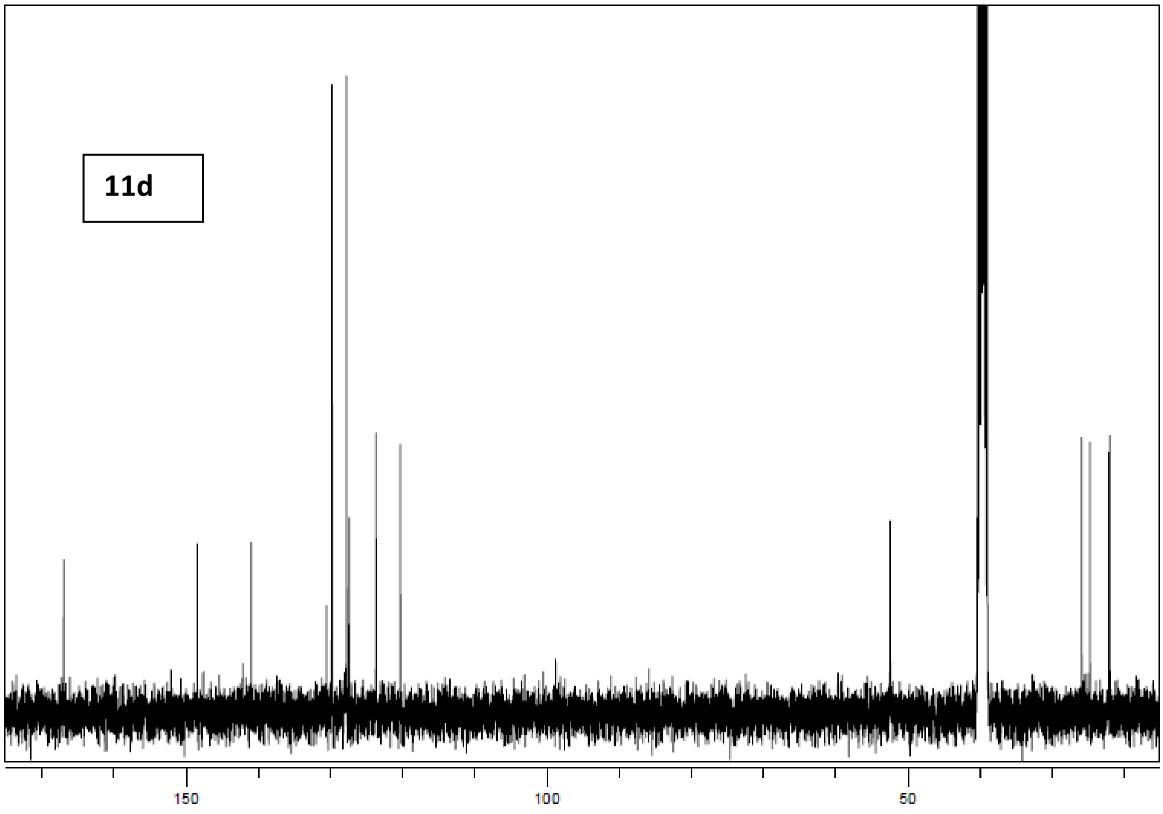

^{1}H NMR (400 MHz, acetone-d_6)

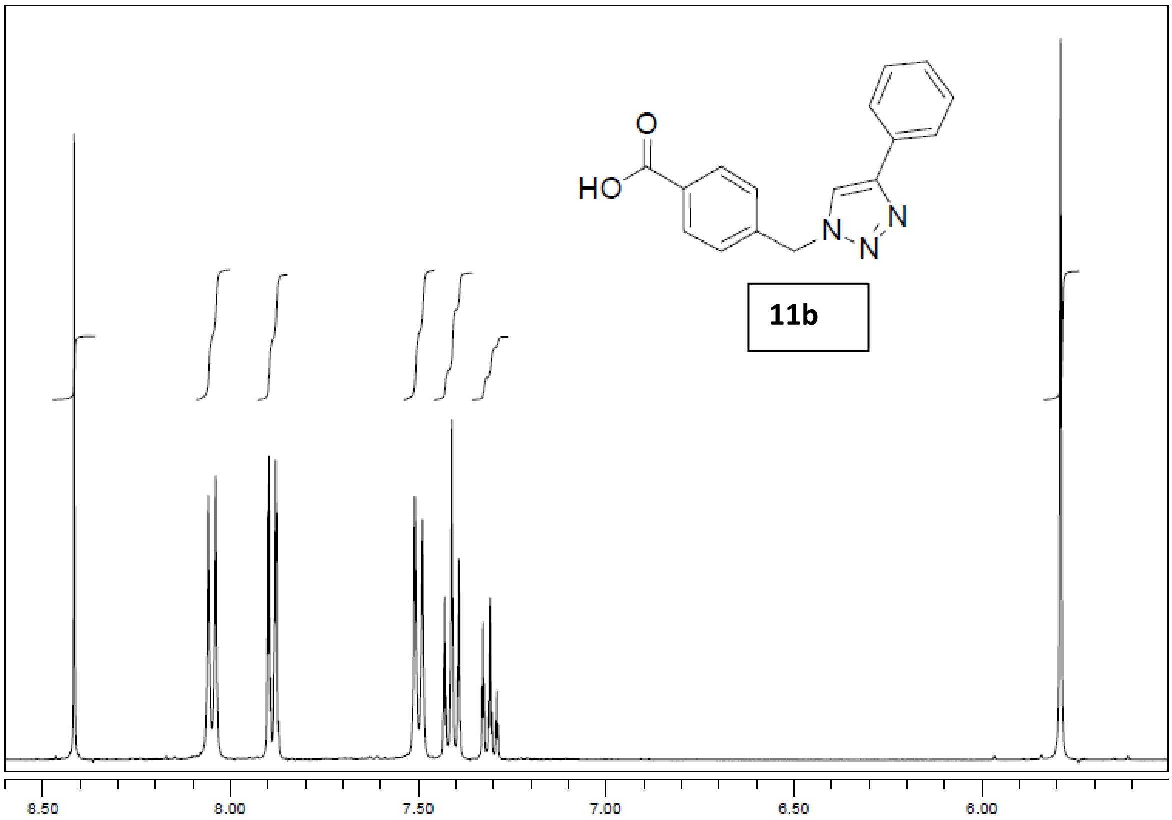

^{13}C NMR (100 MHz, acetone-d_6)

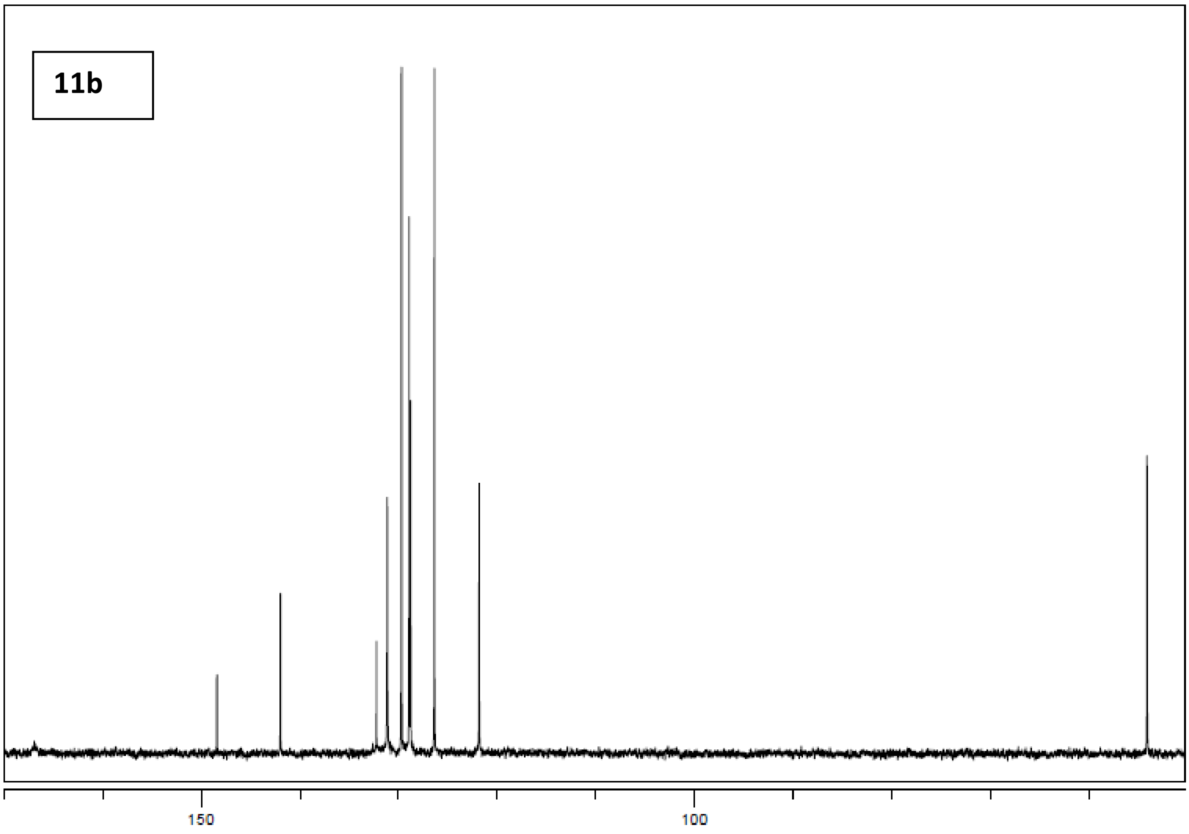

^{1}H NMR (600 MHz, methanol-$d4$)

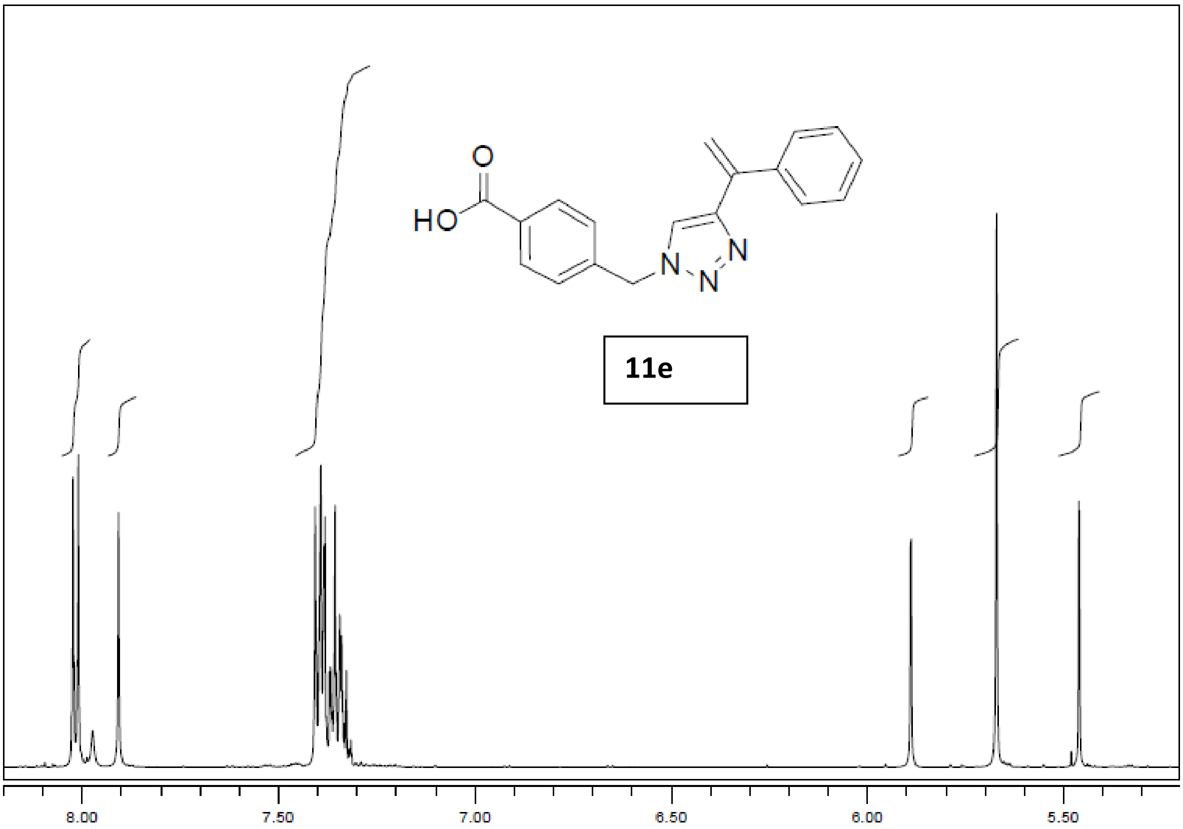

^{13}C NMR (150 MHz, methanol-$d4$)

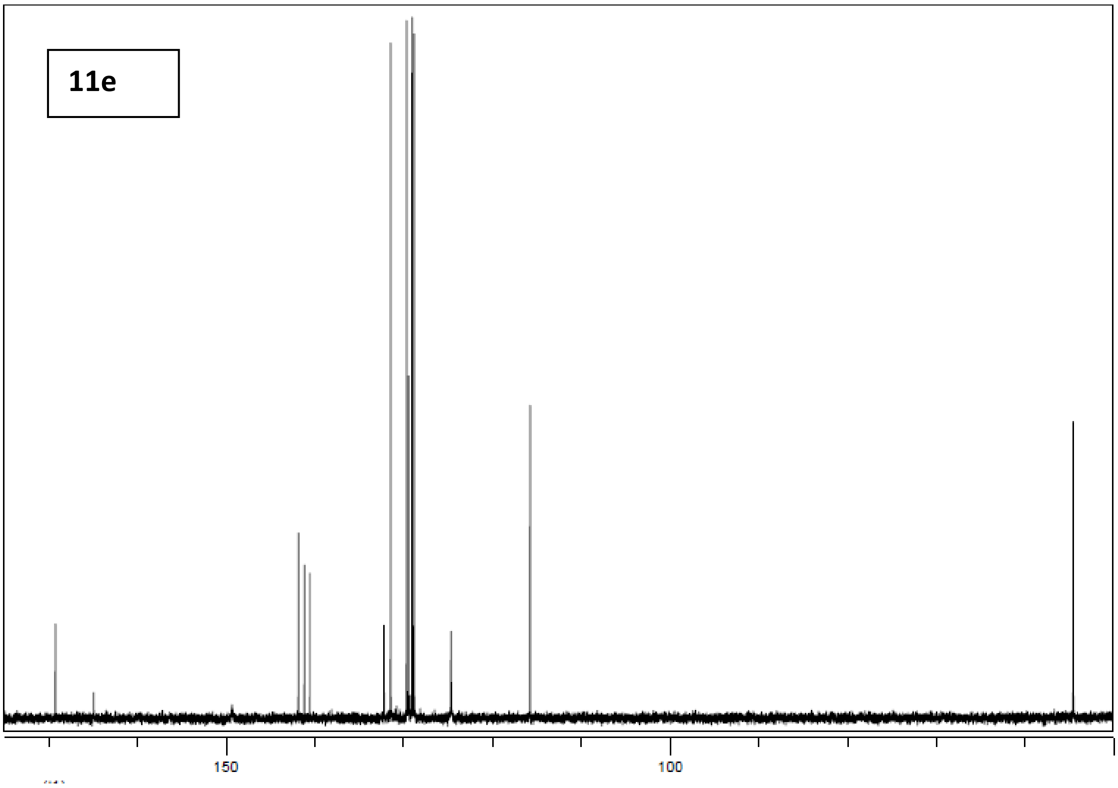

^{1}H-NMR (400 MHz, DMSO-d_6)

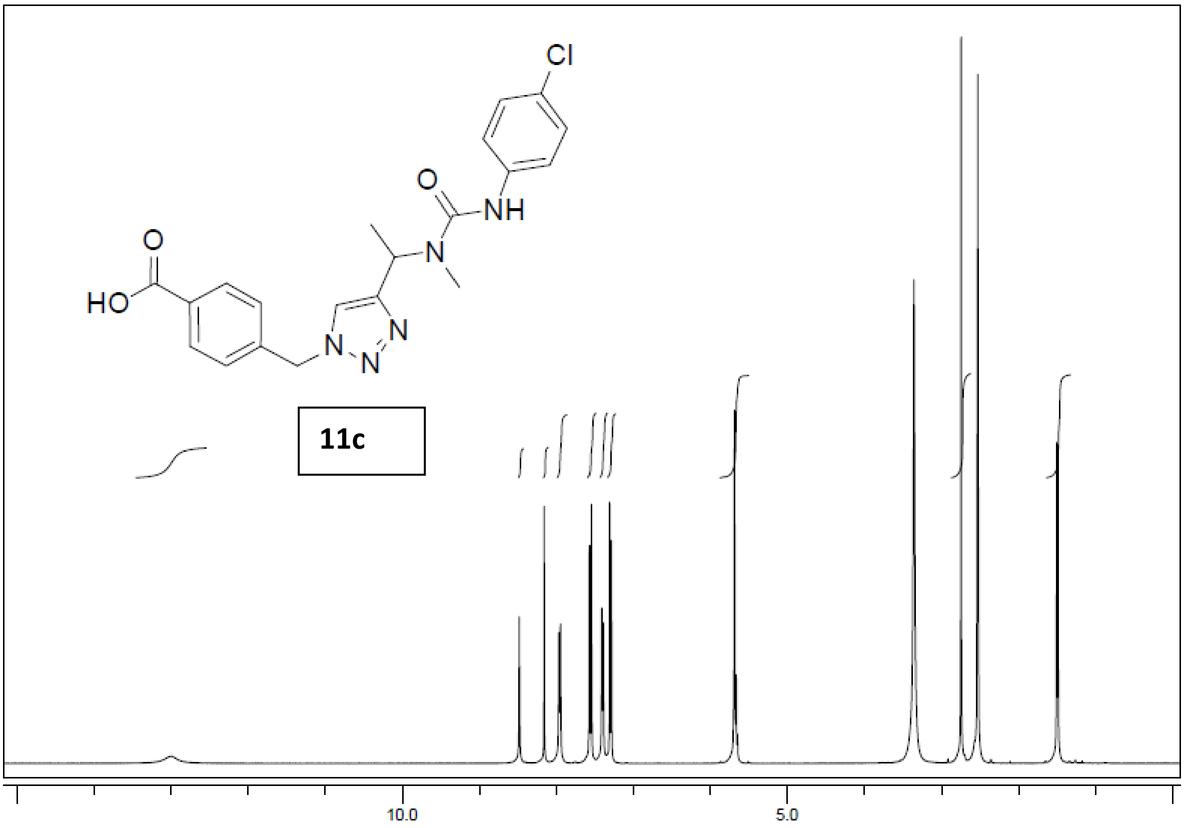

^{13}C-NMR (100 MHz, DMSO-d_6)

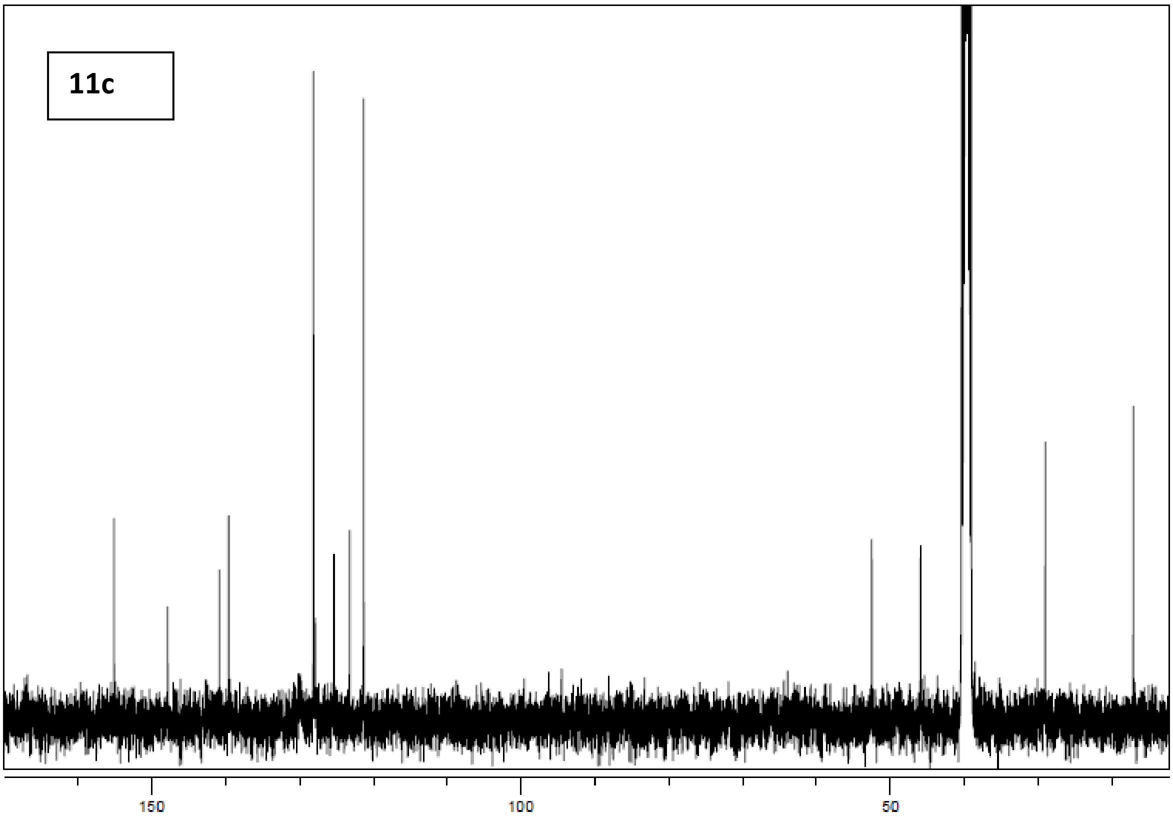

^{1}H-NMR (400 MHz, DMSO-d_6)

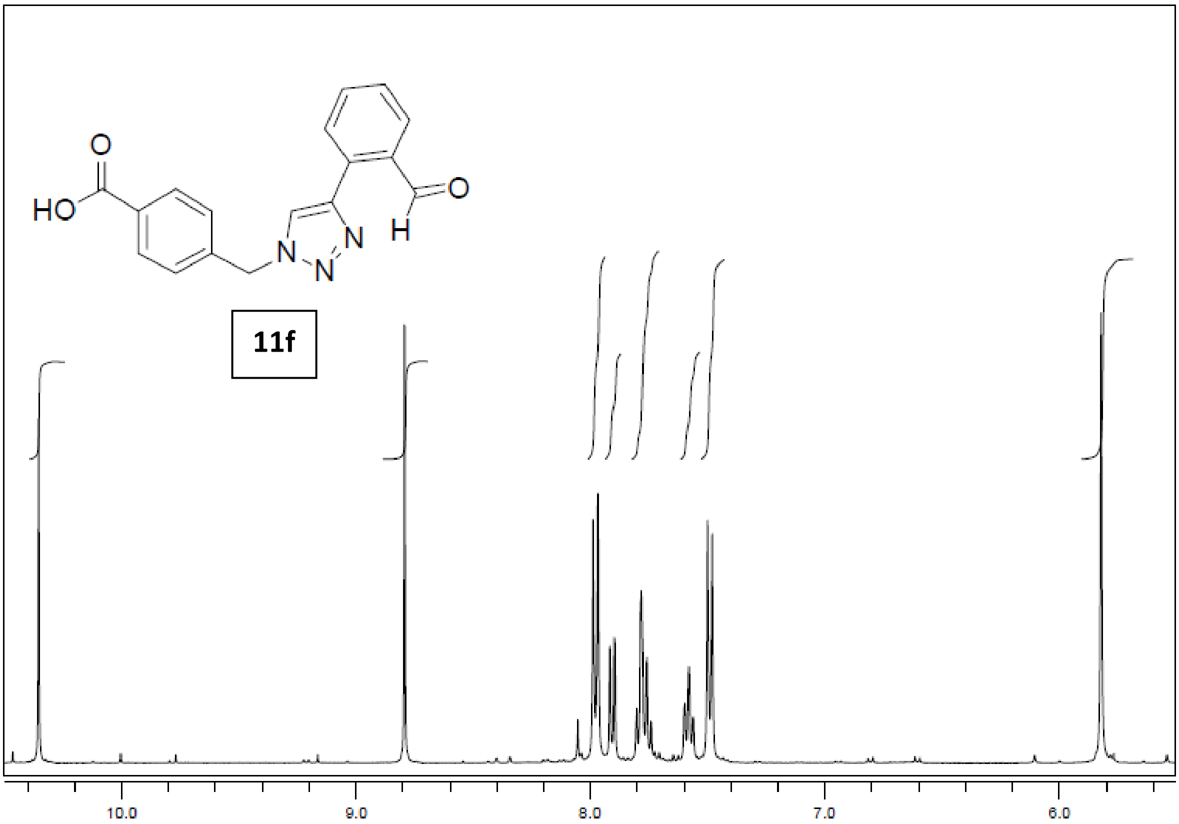

^{13}C-NMR (100 MHz, DMSO-d_6)

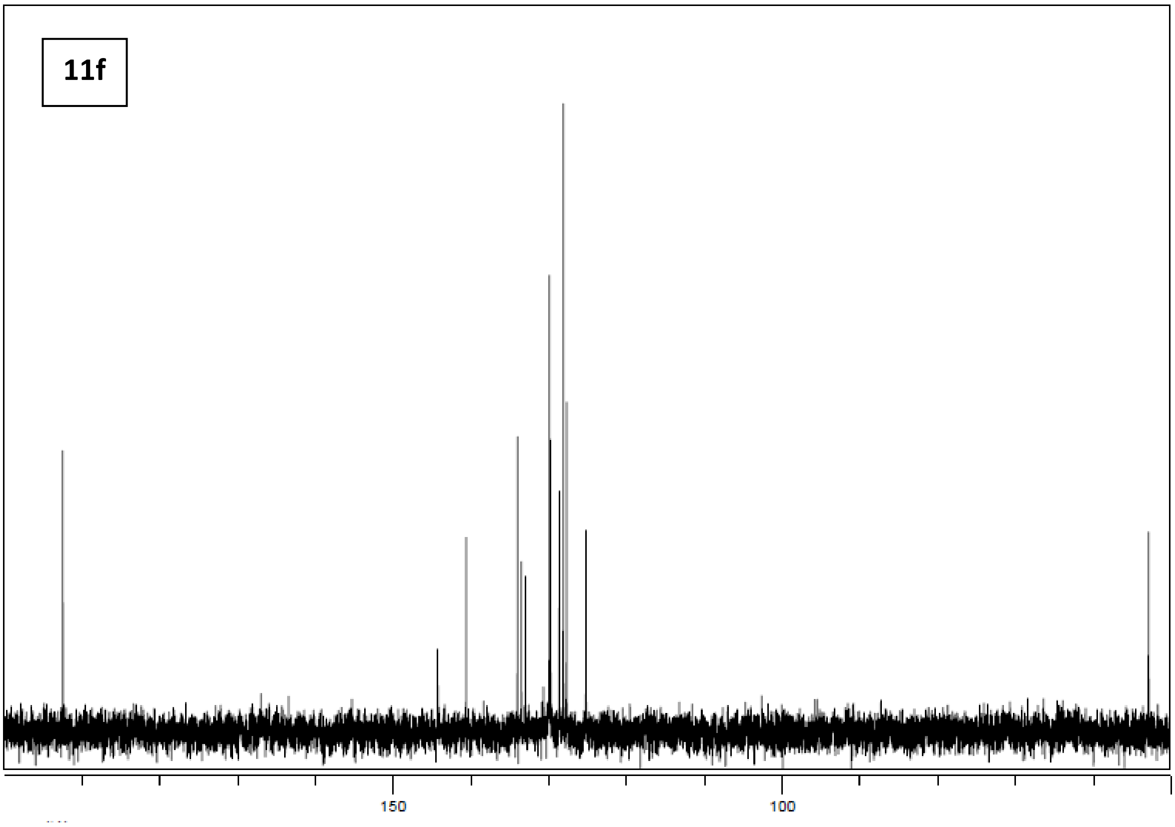

^{1}H-NMR (400 MHz, DMSO-d$_6$)

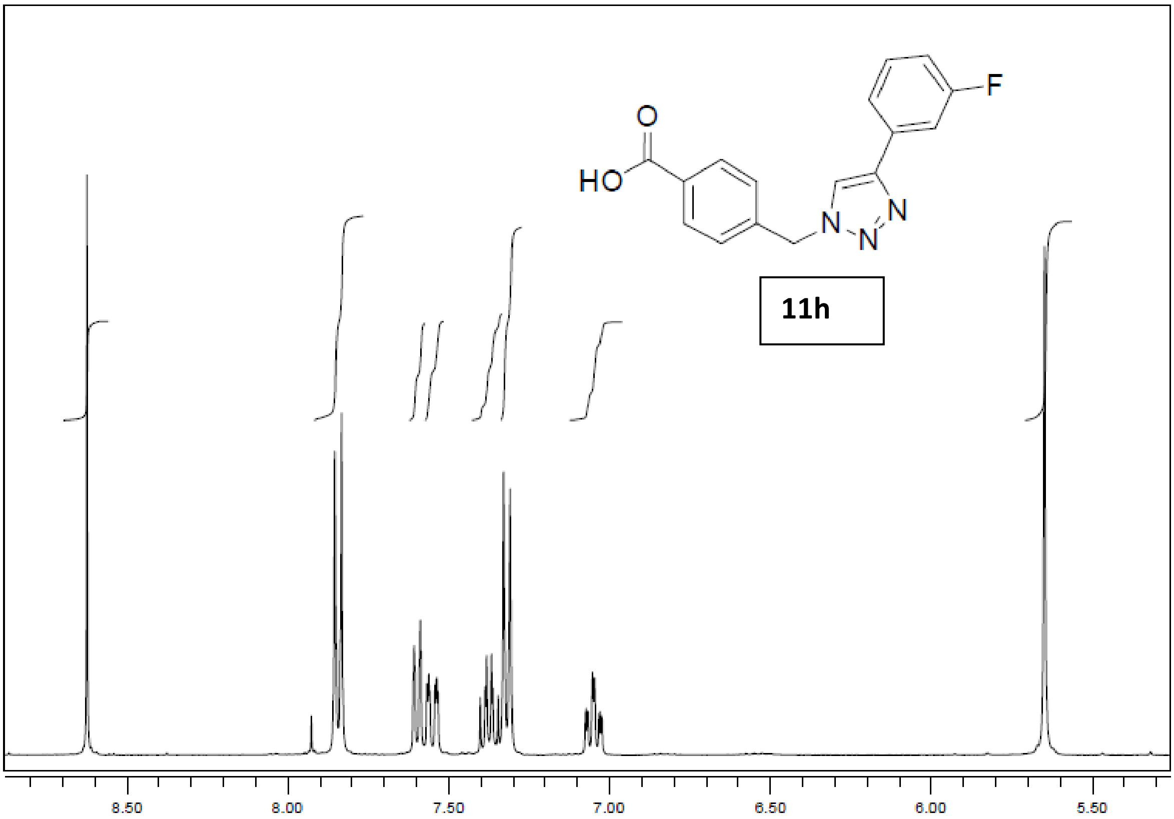

^{13}C-NMR (100 MHz, DMSO-d$_6$)

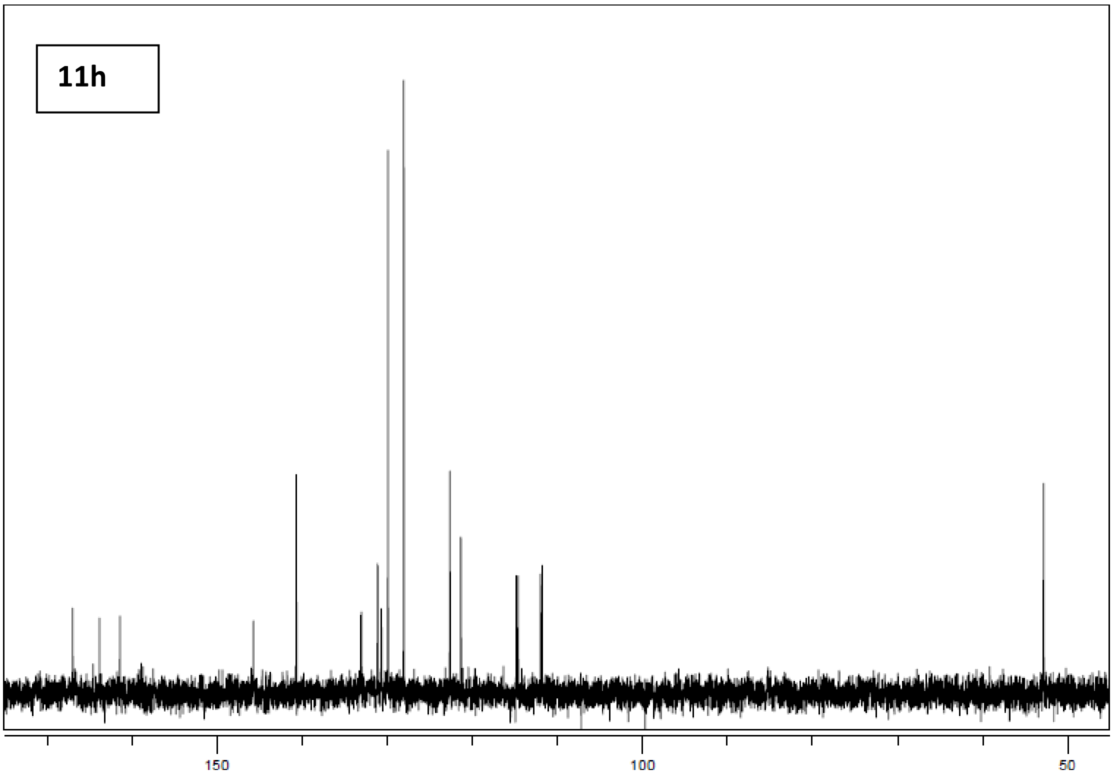

^{1}H-NMR (400 MHz, DMSO-d_6)

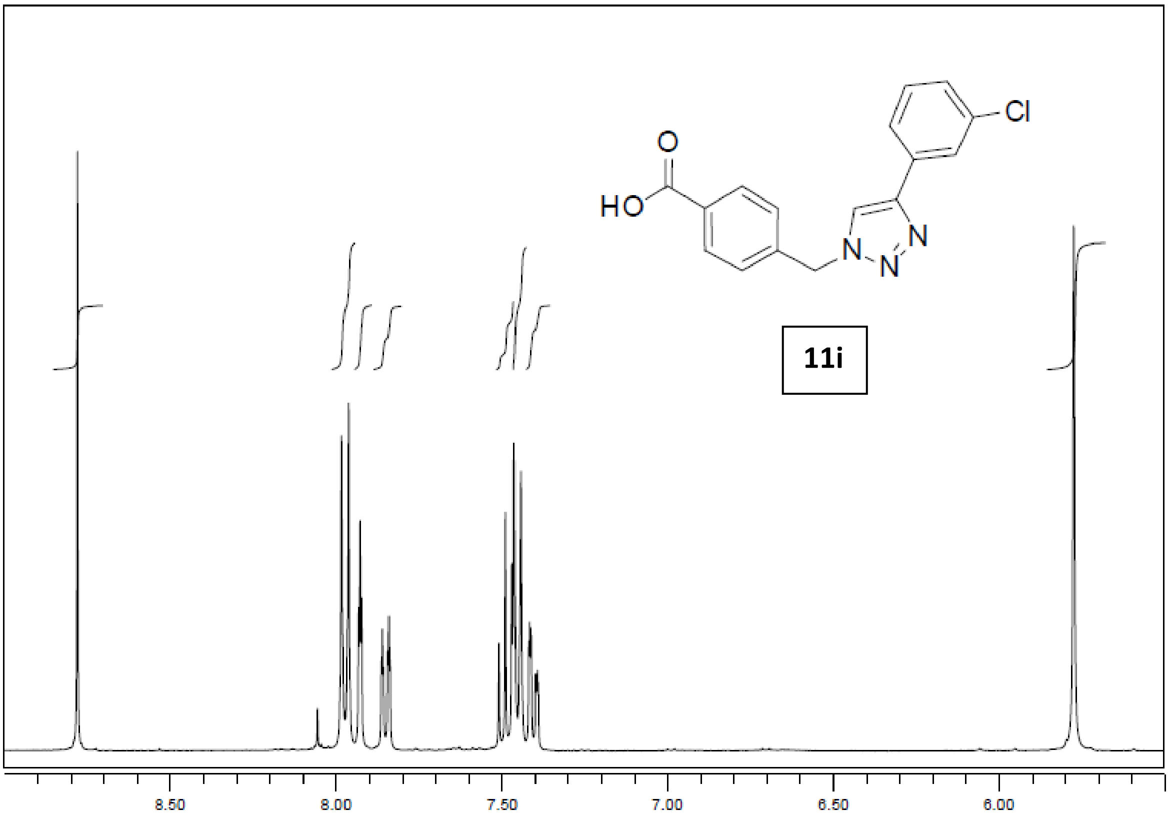

^{13}C-NMR (100 MHz, DMSO-d_6)

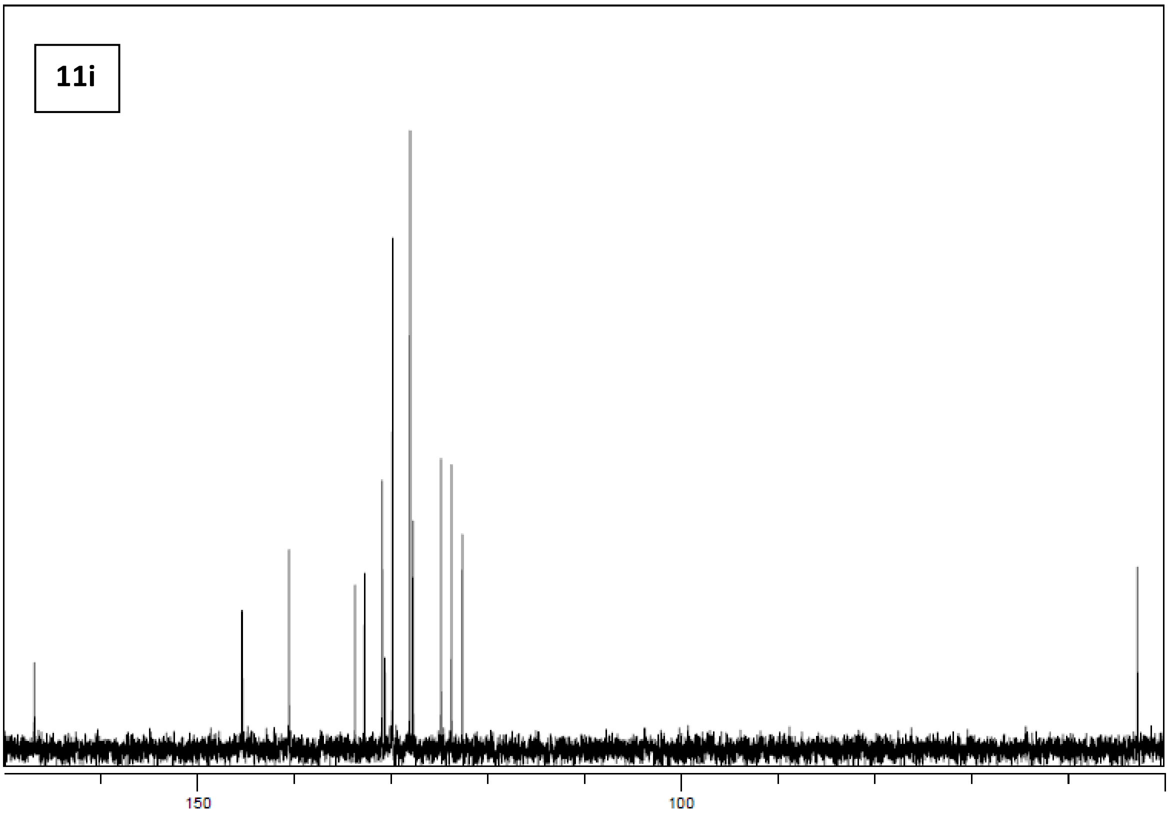

^{1}H-NMR (400 MHz, DMSO-d_6)

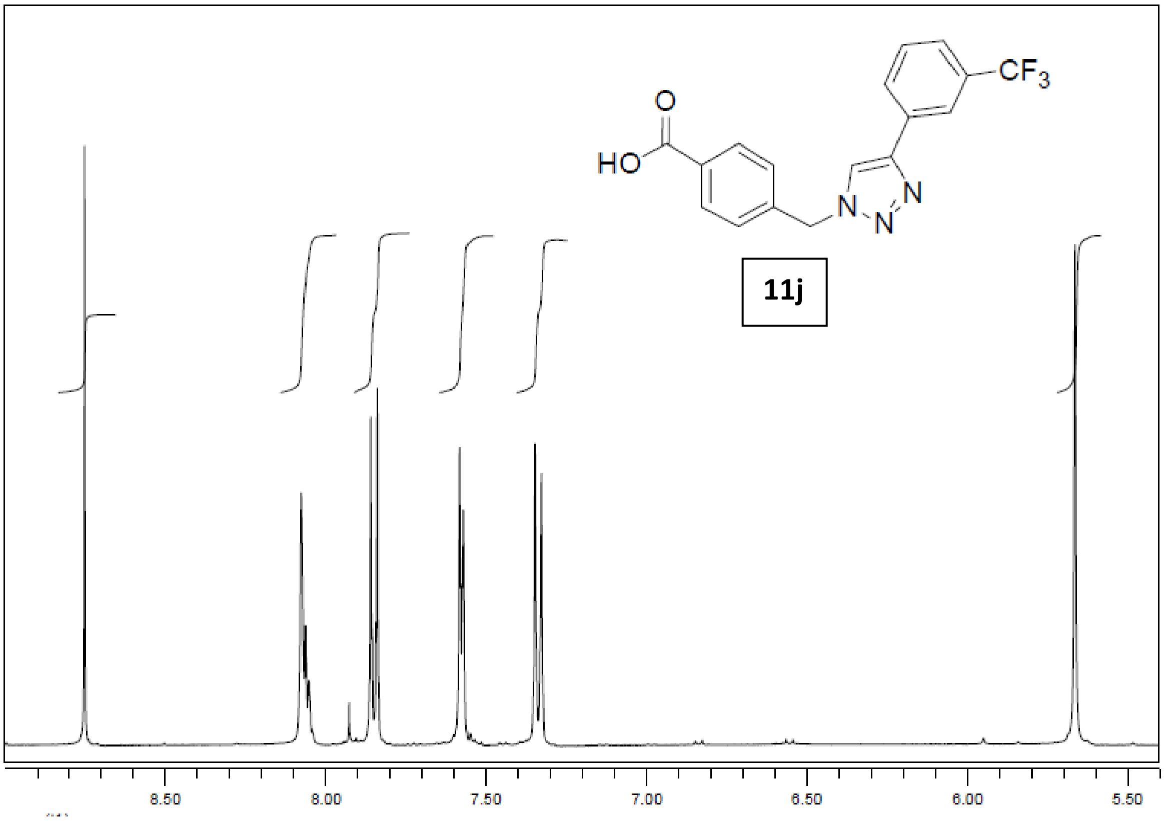

^{13}C-NMR (100 MHz, DMSO-d_6)

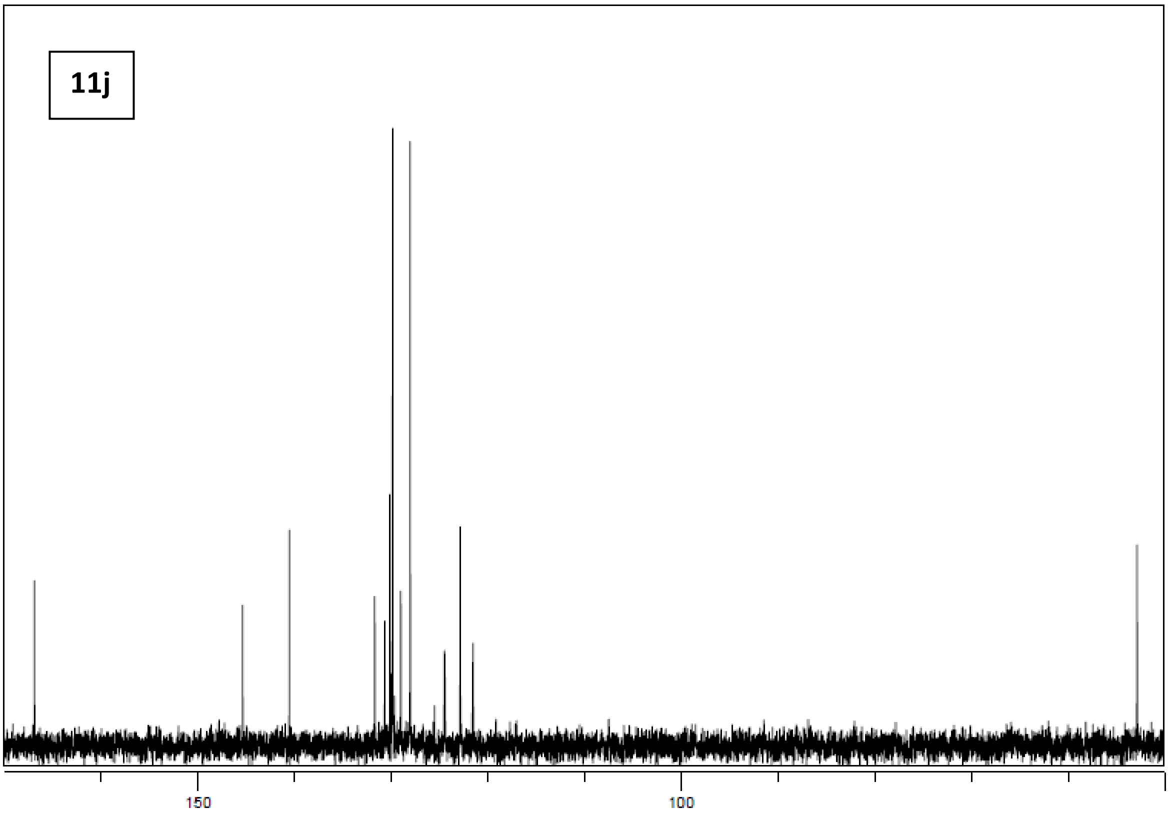

^{1}H-NMR (400 MHz, DMSO-d_6)

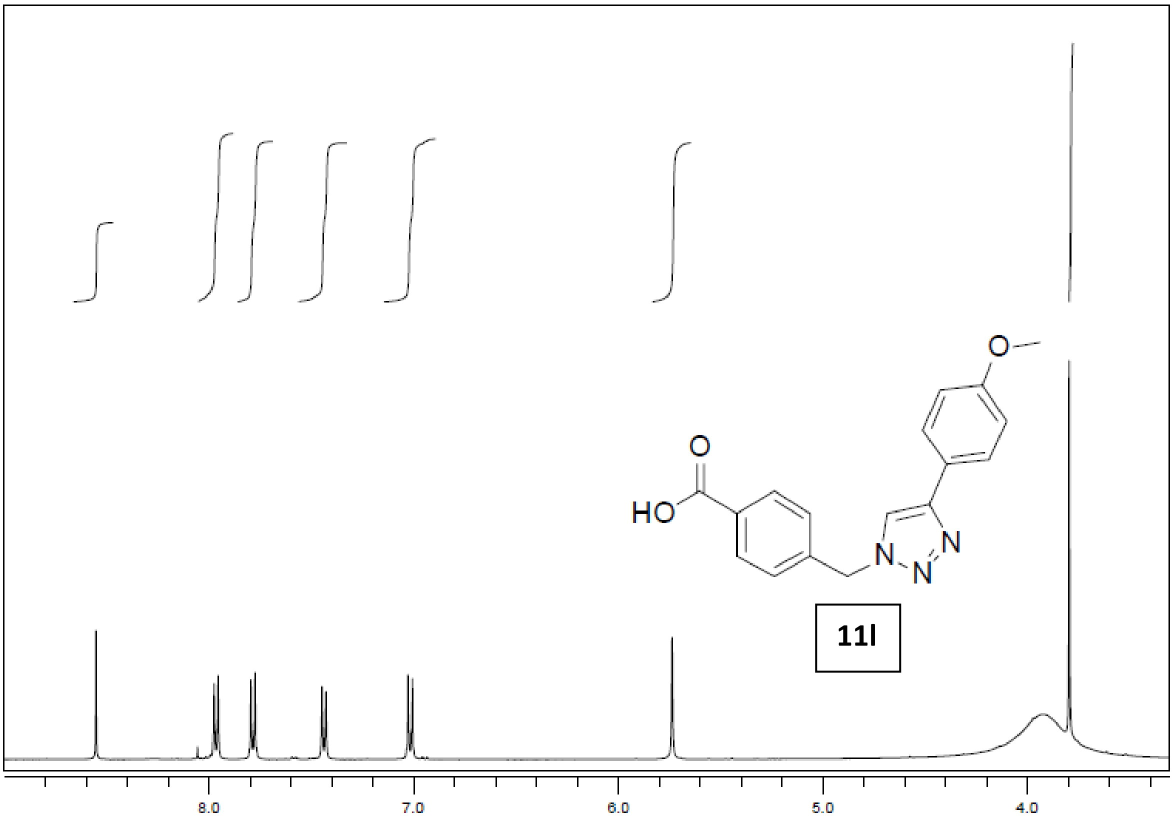

^{13}C-NMR (100 MHz, DMSO-d_6)

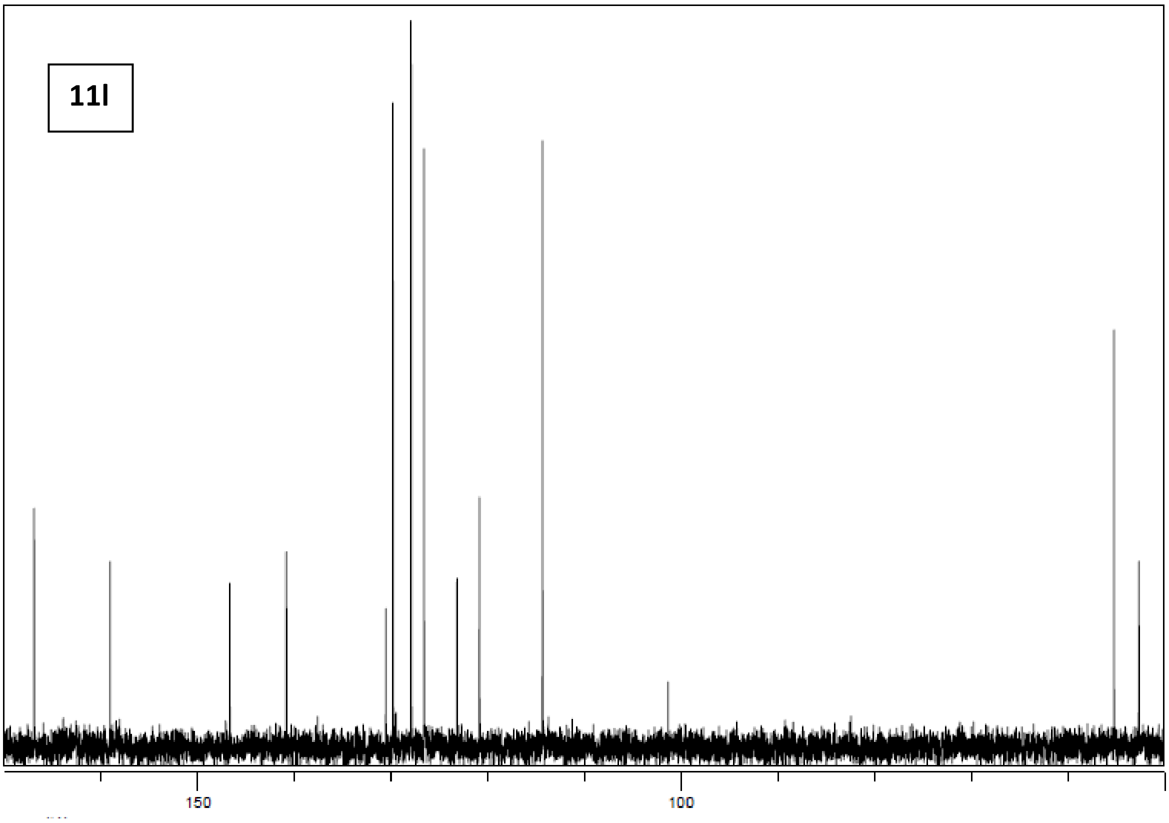

^{1}H-NMR (400 MHz, DMSO-d_6)

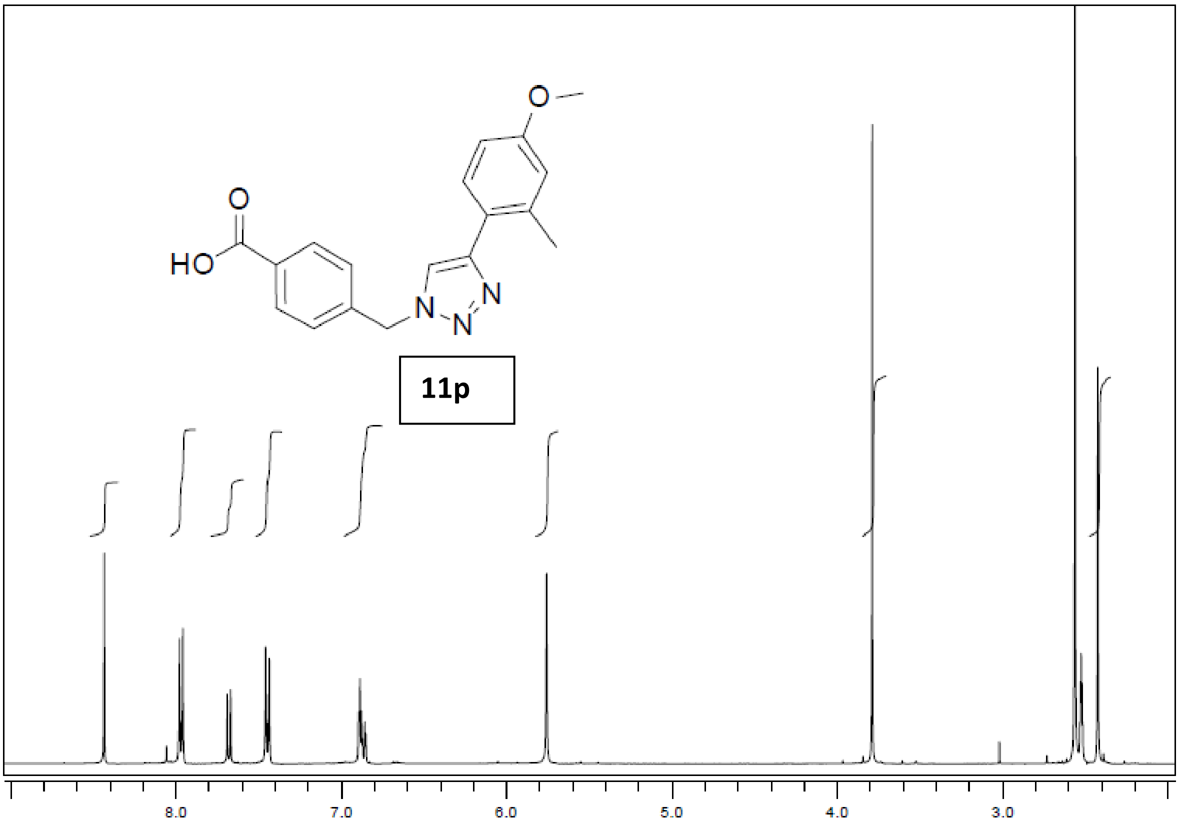

^{13}C-NMR (100 MHz, DMSO-d_6)

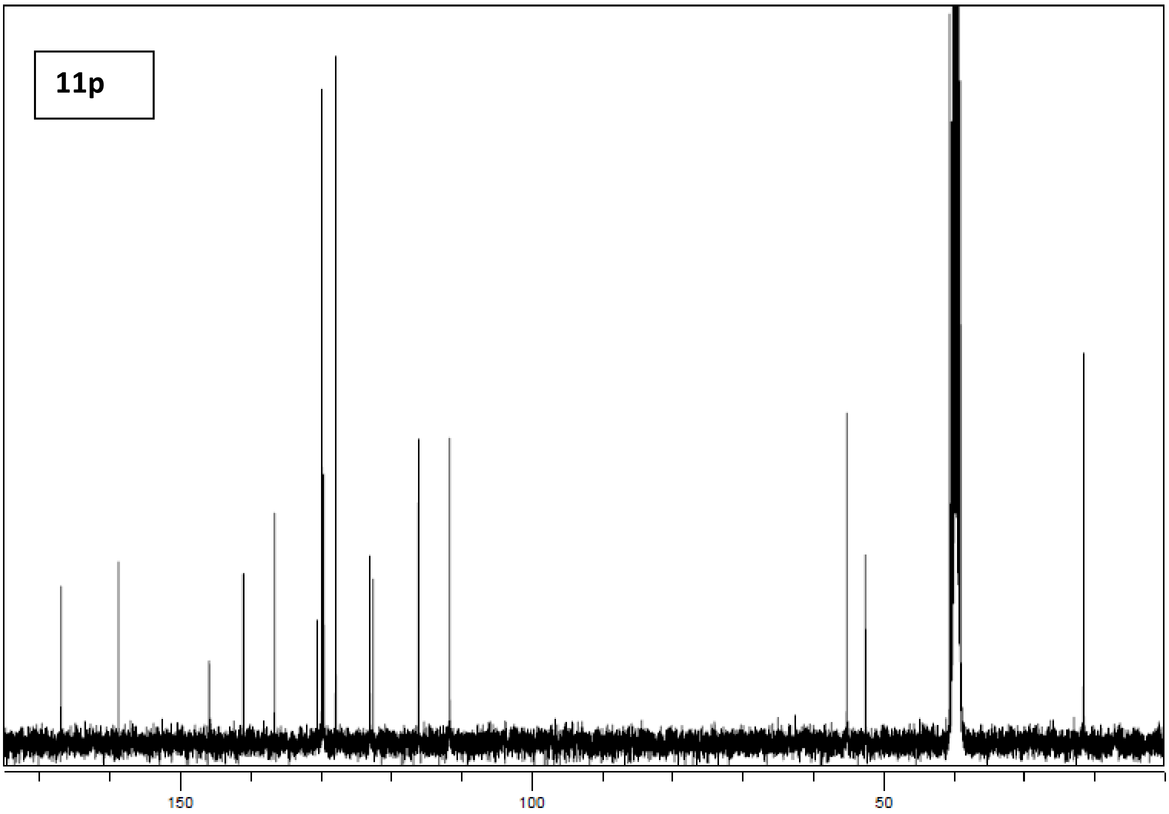

^{1}H-NMR (400 MHz, DMSO-d_6)

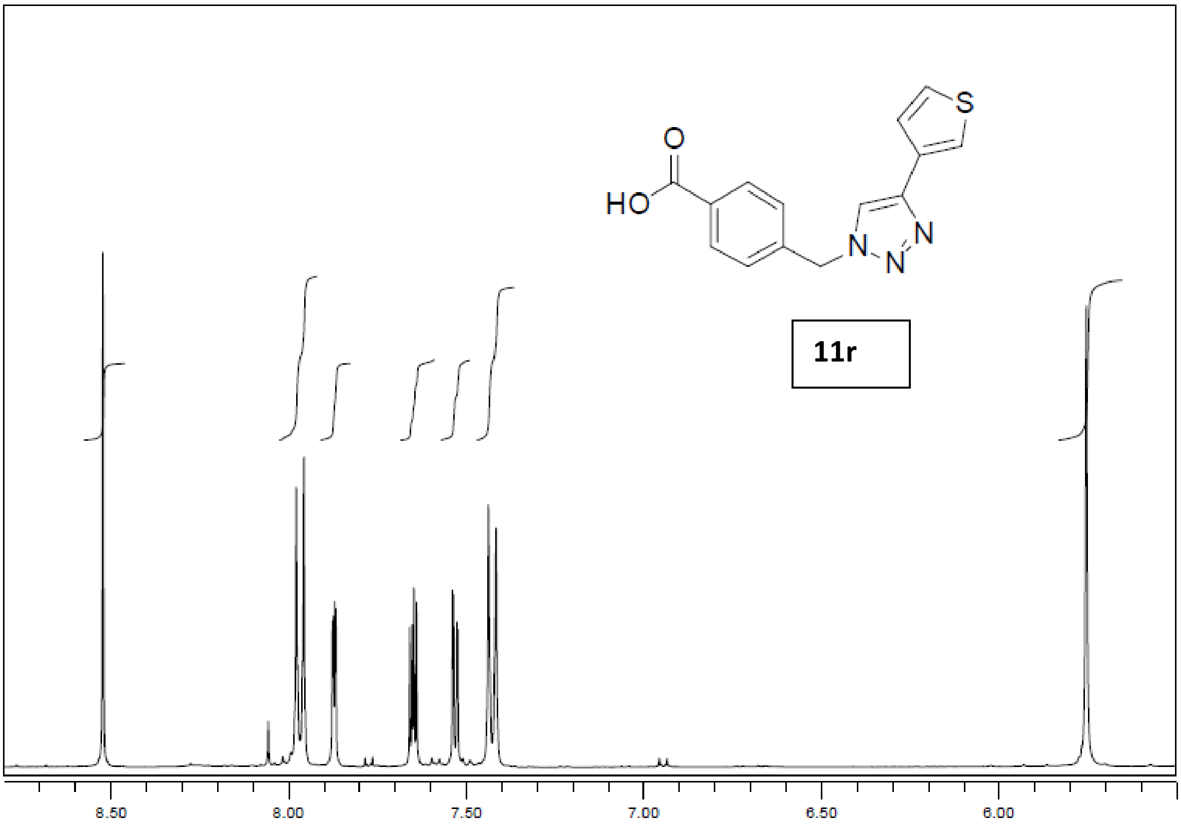

^{13}C-NMR (100 MHz, DMSO-d_6)

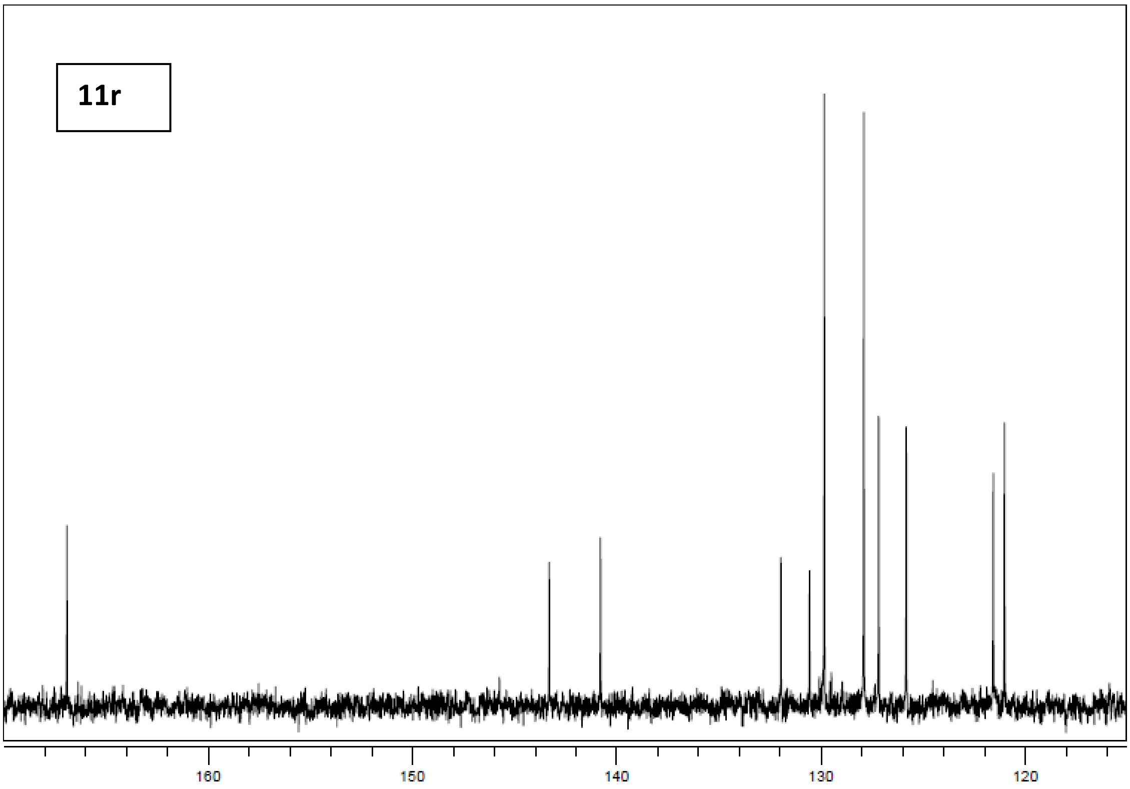

^{1}H-NMR (400 MHz, DMSO-d_6)

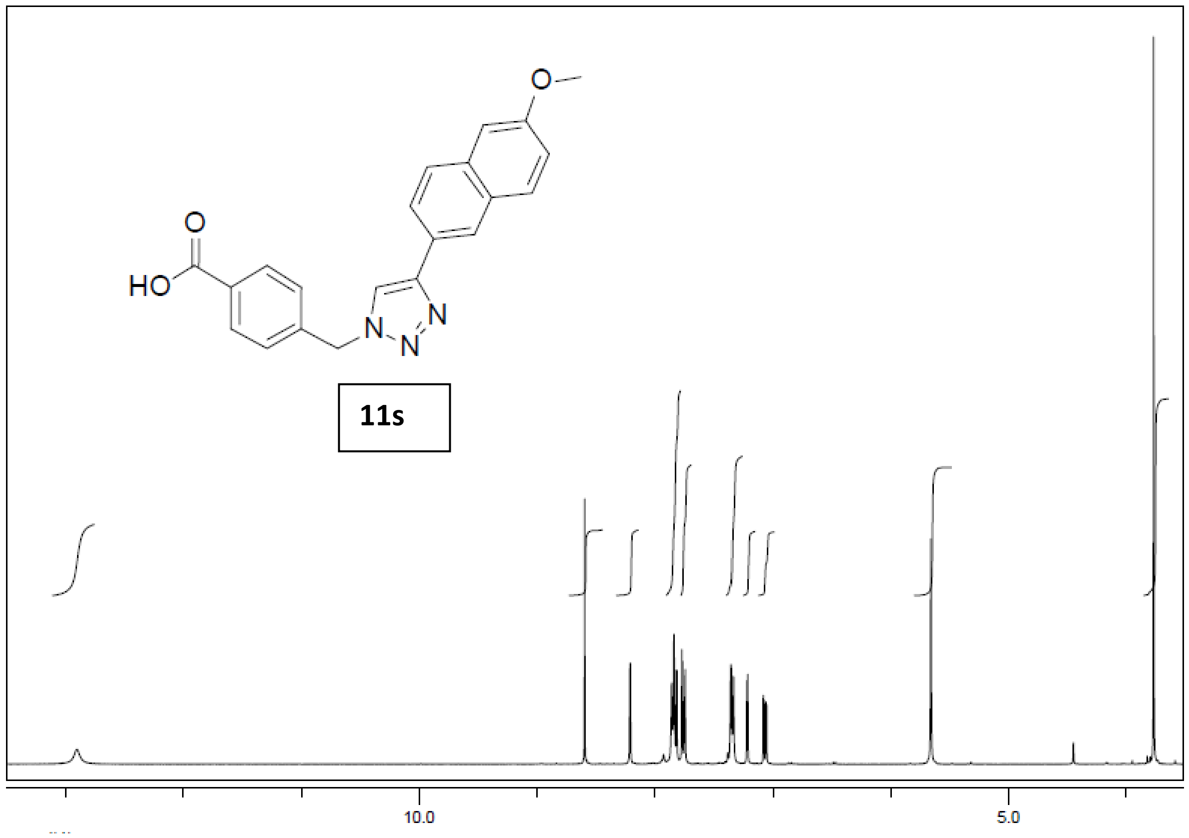

^{13}C-NMR (100 MHz, DMSO-d_6)

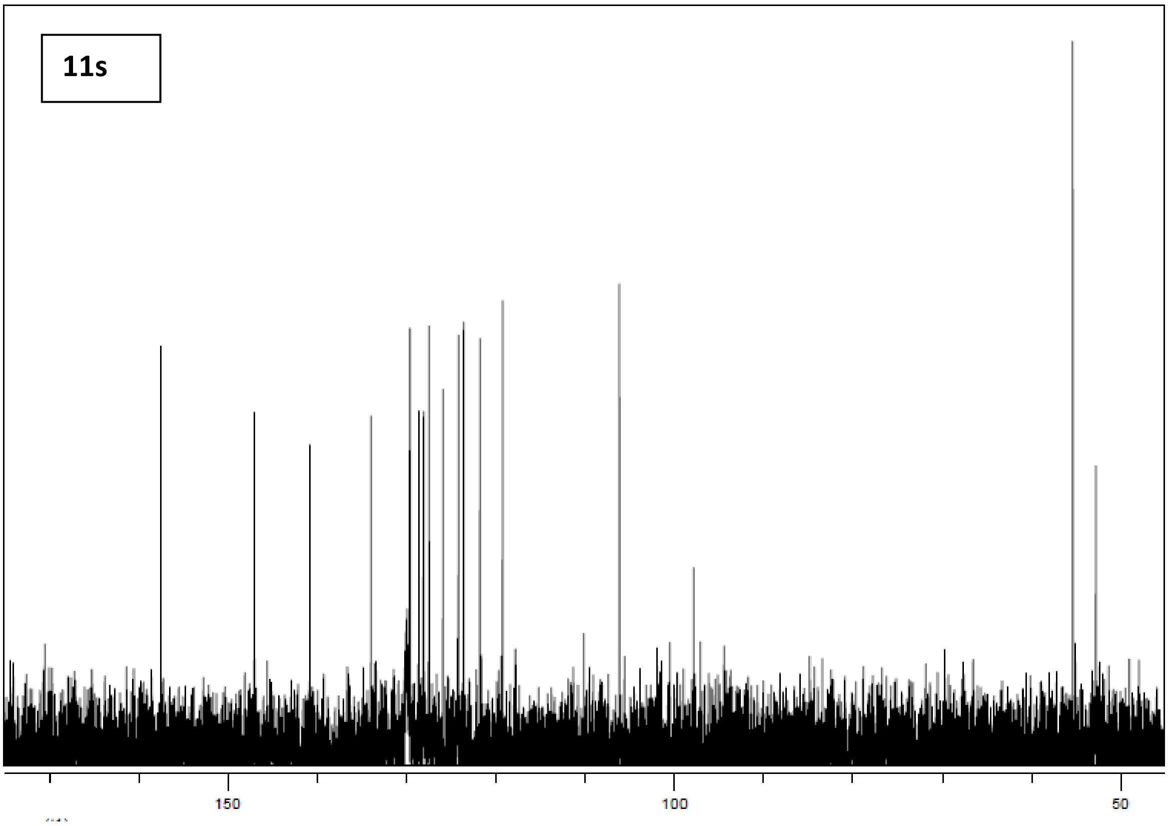

Data on purity of crude material after resin cleavage

Structure	Formula	Calculated exact mass	Crude weight	Crude purity (UV area percent)
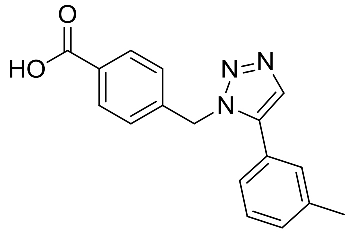	C17H15N3O2	293.1164267	14.7 mg	43.3%
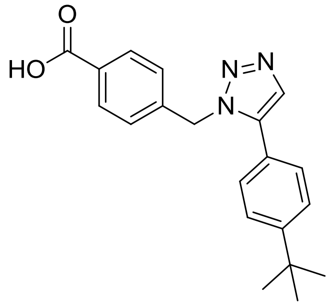	C20H21N3O2	335.1633769	17.6 mg	80.2%
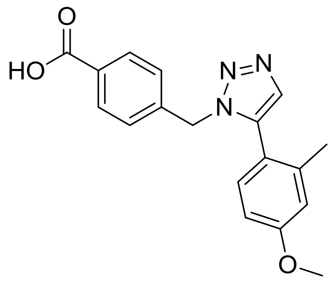	C_{18}H17N3O3	323.1269914	16.1 mg	81.2%
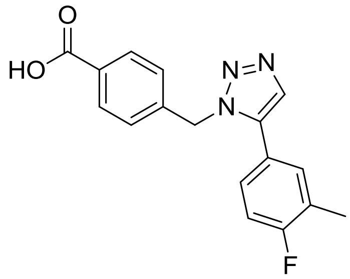	$C_{17}H_{14}FN_3O_2$	311.1070049	23.0 mg	88.8%

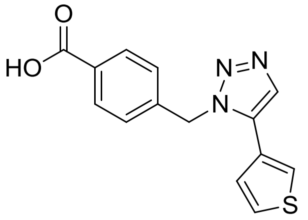	$C_{14}H_{11}N_3O_2S$	285.0571973	22.6 mg	92.3%
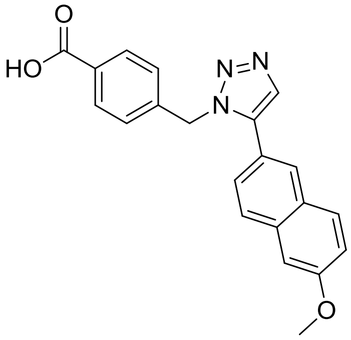	$C_{21}H_{17}N_3O_3$	359.1269914	21.2 mg	95.0%
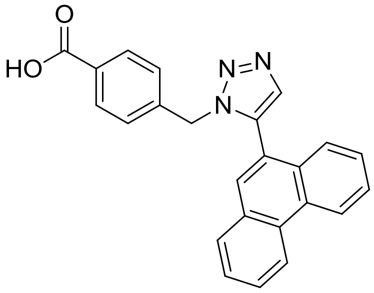	$C_{24}H_{17}N_3O_2$	379.1320768	17.9 mg	74.3%
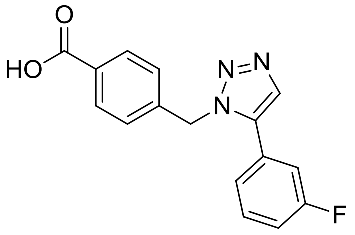	$C_{16}H_{12}FN_3O_2$	297.0913548	25.7 mg	88.8%
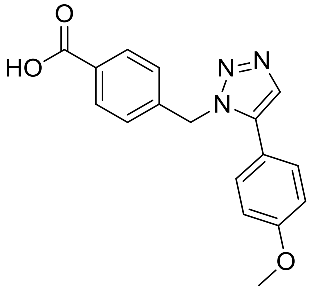	$C_{17}H_{15}N_3O_3$	309.1113414	23.6 mg	94.2%

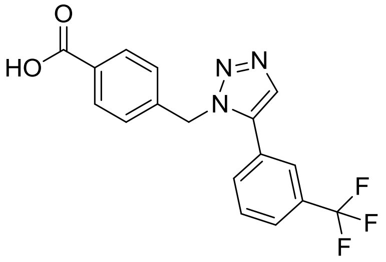	$C_{17}H_{12}F_3N_3O_2$	347.0881612	26.0 mg	90.3%
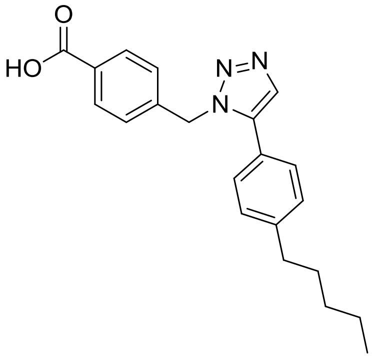	$C_{21}H_{23}N_3O_2$	349.179027	19.2 mg	82.3%
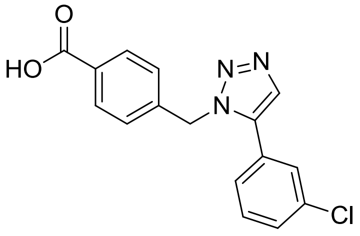	$C_{16}H_{12}ClN_3O_2$	313.0618044	24.1 mg	89.1%
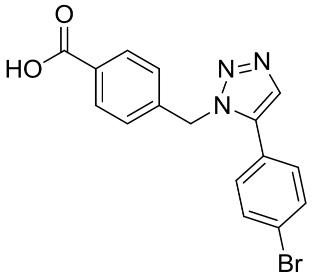	$C_{16}H_{12}BrN_3O_2$	357.0112892	28.6 mg	91.5%
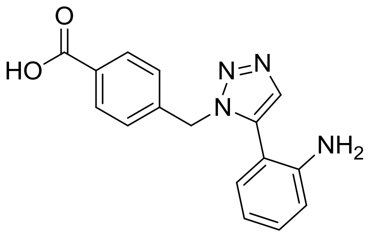	$C_{16}H_{14}N_4O_2$	294.1116757	40.4 mg	64.7%

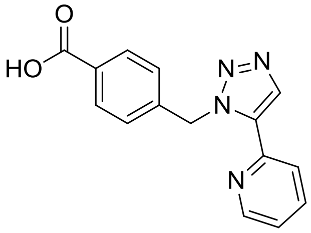	$C_{15}H_{12}N_4O_2$	280.0960257	15.2 mg	91.5%
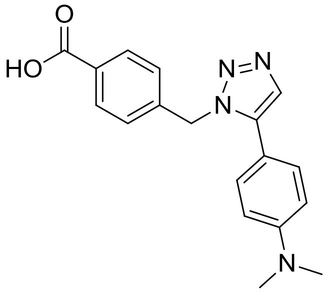	$C_{18}H_{18}N_4O_2$	322.1429758	34.1 mg	96.3%
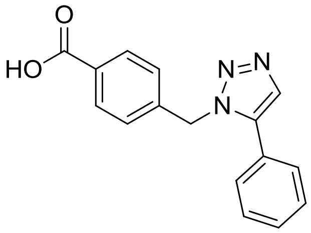	$C_{16}H_{13}N_3O_2$	279.1007767	29.2 mg	91.0%
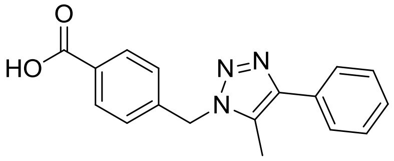	$C_{17}H_{15}N_3O_2$	293.1164267	17.4 mg	78.1%
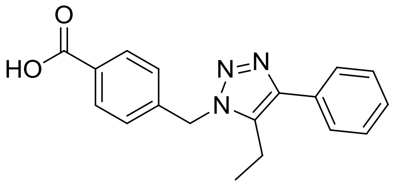	$C_{18}H_{17}N_3O_2$	307.1320768	20.0 mg	83.7%
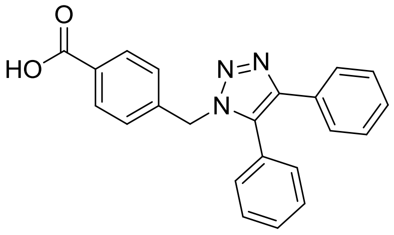	$C_{22}H_{17}N_3O_2$	355.1320768	27.2 mg	97.9%

References

1. Sonawane, N. D.; Verkman, A. S. *Bioorg. Med. Chem.* **2008,** *16,* 8187–8195. doi:10.1016/j.bmc.2008.07.044
2. Almstead, N.; Karp, G. M.; Wild, R.; Welch, E.; Campbell, J. A.; Ren, H.; Chen, G. Preparation of diaryl substituted pyrazoles and analogs for nonsense suppression. WO 2006044502, June 27, 2006.
3. Wang, M.; Das, M. R.; Li, M.; Boukherroub, R.; Szunerits, S. *J. Phys. Chem. C* **2009,** *113,* 17082–17086. doi:10.1021/jp904501q
4. Coelho, A.; Diz, P.; Caameno, O.; Sotelo, E. *Adv. Synth. Catal.* **2010,** *352,* 1179–1192. doi:10.1002/adsc.200900680
